# Endo-SOFT: study protocol for a national multicentre randomised controlled trial – first-line surgery versus first-line assisted reproductive technologies in patients with advanced endometriosis

**DOI:** 10.1136/bmjopen-2025-111928

**Published:** 2026-03-25

**Authors:** Anna Marklund, Julia Wängberg Nordborg, Christine Asciutto, Evangelia Elenis, Jynfiaf Francis, Ligita Jokubkiene, Kristin Wennmo Zuk, Hanna Åmark, Kenny A Rodriguez-Wallberg, Karin Sundfeldt, Maria Forslund, Malin Brunes

**Affiliations:** 1 Department of Reproductive Medicine, Karolinska University Hospital, Stockholm, Sweden; 2 Department of Oncology and Pathology, Karolinska Institutet, Stockholm, Sweden; 3 Department of Obstetrics and Gynecology, Institute of Clinical Sciences, Sahlgrenska Academy, University of Gothenburg, Gothenburg, Sweden; 4 Department of Gynecology and Reproductive Medicine, Sahlgrenska University Hospital, Gothenburg, Sweden; 5 Department of Obstetrics and Gynecology, Uppsala University Hospital, Uppsala, Sweden; 6 Women's and Children’s Health, Uppsala Universitet, Uppsala, Sweden; 7 Obstetric, Gynaecological and Prenatal Ultrasound Research, Department of Clinical Sciences, Lund University, Malmo, Sweden; 8 Department of Obstetrics and Gynecology, Skåne University Hospital, Malmö, Sweden; 9 Department of Obstetrics and Gynecology, Södersjukhuset, Stockholm, Sweden; 10 Departement of Clinical Science and Education Södersjukhuset, Karolinska Institutet, Stockholm, Sweden; 11 Department of Reproductive Medicine, Division of Gynecology and Reproduction, Karolinska Universitetssjukhuset, Stockholm, Sweden; 12 Department of Obstetrics and Gynecology, Sahlgrenska University Hospital, Goteborg, Sweden

**Keywords:** endometriosis, fertility, surgery, ART, IVF

## Abstract

**Introduction:**

Endometriosis is a chronic, inflammatory and oestrogen-dependent disease frequently causing infertility. Assisted reproductive technologies (ART) are often required to achieve pregnancy, especially in women with advanced endometriosis. To date, there is no randomised controlled trial (RCT) exploring reproductive outcomes after first-line surgery followed by ART versus first-line ART in women with stage III-IV endometriosis. The aim in this study is to investigate if endometriosis surgery prior to ART in women with endometriosis stage III-IV and infertility results in improved reproductive outcomes compared with first-line ART.

**Methods and analysis:**

A national multicentre prospective open label RCT including 350 subjects with stage III-IV endometriosis and infertility. Women aged 18–38 will be randomised 1:1 to endometriosis surgery prior to ART or first-line ART. Follow-up until 3 years from the first treatment.

The primary outcome is the cumulative live birth rate 3 years after inclusion. Secondary outcomes are cumulative pregnancy rate, reproductive outcomes per in vitro fertilisation cycle, miscarriage rate, time to pregnancy and live birth, plasma progesterone levels on the day of frozen embryo transfer in hormone replacement cycles, obstetrical outcomes and complications, infections after oocyte retrieval, intraoperative and postoperative complications, pain, quality of life and cost-effectiveness.

**Ethics and dissemination:**

The study protocol, patient information and informed consent are approved by Swedish Ethical Review Authority (dnr: 2024–04293-01, approved 7 October 2024, amendment, dnr: 2025–03699-02, approved 17 June 2025).

An interim analysis is scheduled to ensure the safety of the interventions.

The results of Endo-SOFT (Endometriosis: Surgery Or Fertility Treatment) will be published in scientific journals and are expected to influence clinical guidelines.

**Trial registration number:**

ClinicalTrials.gov, NCT07240363.

**STRENGTHS AND LIMITATIONS OF THIS STUDY**

- Randomisation minimises selection bias and balances both known and unknown confounders.
- The multicentre design and the centralisation of advanced endometriosis surgery to highly specialised centres ensures condensed knowledge about screening and diagnosing advanced endometrioses as well as advanced operative skills, and the multicentre design will ensure adequate population size.
- The study includes comprehensive outcome measures, including reproductive outcomes, safety, quality of life and cost-effectiveness, with patient involvement incorporated during trial planning, including a qualitative study capturing patients’ perspectives.
- Limitations include the open-label design, potential centre-related effects inherent to a complex multicentre intervention and the exclusion of women with previous endometriosis surgery or use of assisted reproductive technologies, which may limit generalisability.
- Patient treatment preferences and potential crossover between strategies may influence adherence and dilute treatment effects, although intention-to-treat analyses are planned.

## Introduction

 Endometriosis is a chronic, inflammatory and oestrogen-dependent disease in which endometrial cells are located outside the uterine cavity. Common symptoms include severe menstrual pain, dyspareunia, chronic pelvic pain, gastrointestinal or urinary symptoms and infertility. The disease affects about 10% of women and for many, it has a major impact on both physical and mental health.^1 2^ In addition to personal suffering, endometriosis also imposes an economic burden on both the individual and society.^3^

Women with endometriosis have reduced chances of conceiving spontaneously, with a twofold increased risk of infertility compared with women without the disease.^4 5^ Endometriosis may impair fertility through several mechanisms: adhesions and structural abnormalities disrupting reproductive organ function; diminished ovarian reserve; and impaired embryo implantation through immunological and inflammatory pathways.^6^ Among infertile women, deep endometriosis has been identified in 17% of cases,^7^ and when peritoneal endometriosis is also considered, the prevalence may reach up to 40%. About 50% of women with mild endometriosis achieve spontaneous pregnancy, compared with 25% of those with moderate disease, while spontaneous pregnancy is rare in cases of severe endometriosis.^8^ Thus, assisted reproductive technologies (ART) are often required to achieve pregnancy in women with advanced endometriosis. However, a systematic review and meta-analysis showed that, even with fertility treatment, these women have lower pregnancy and live birth rates compared with those without the disease.^9^

Whether surgery prior to ART may improve reproductive outcomes in this population remains unclear. Current guidelines from the European Society of Human Reproductive and Embryology (ESHRE) recommend that surgery prior to ART in women with advanced endometriosis may be considered to alleviate endometriosis-associated pain or improve follicle accessibility, but not solely to enhance reproductive outcomes.^1^ In a 2024 systematic review and meta-analysis, Liang *et al* concluded that first-line surgery and first-line ART in women with deep infiltrating endometriosis result in similar fertility outcomes.^10^ This review included six cohort studies but no randomised controlled trials (RCTs). The authors also highlighted that excluding a single study substantially altered the results, favouring first-line surgery. Other important outcomes such as complications, quality of life and pain were not analysed.

To our knowledge, no RCT has yet explored reproductive outcomes after first-line surgery followed by ART versus first-line ART in women with stage III-IV endometriosis according to the revised American Society of Reproductive Medicine.^11^ Some retrospective register studies have reported higher pregnancy and live birth rates after first-line surgery.^12 13^ Conversely, two systematic reviews and meta-analyses found no significant impact of endometrioma surgery on ART outcomes.^14 15^ Moreover, surgery has been associated with diminished ovarian reserve, reflected in lower antral follicle counts, higher gonadotropin requirements and fewer oocytes retrieved from the operated ovary compared with the contralateral normal ovary.^14^

### Study rationale

Although the role of surgery in managing infertility related to advanced endometriosis has been debated for many years, high-quality evidence on this issue remains lacking. Existing studies are largely retrospective or single-arm, often confounded by disease severity, adenomyosis and prior surgeries.

There is biological plausibility that surgery could benefit ART outcomes by reducing inflammatory burden, removing endometriomas that may impair oocyte retrieval and restoring pelvic anatomy. Conversely, surgery may harm reproductive potential by diminishing ovarian reserve, inducing pelvic adhesions, delaying time to ART or exposing patients to perioperative risks. This clinical equipoise, together with wide practice variation, underscores the need for a randomised trial. Our aim is to address this gap in a national collaboration between Swedish highly specialised centres for endometriosis surgery and ART clinics.

Here we report a summary of our protocol for a multicentre, prospective RCT—Endo-SOFT (Endometriosis: Surgery Or Fertility Treatment)—that will explore the impact of endometriosis surgery prior to ART on reproductive outcomes in women with infertility and stage III-IV endometriosis. The trial will also evaluate key secondary outcomes, including pregnancy and perinatal complications, quality of life, pain relief and health economic parameters. We adhere to the International Council for Harmonisation—Good Clinical Practice (GCP) and Standard Protocol Items: Recommendations for Interventional Trials (SPIRIT) guidelines. The complete protocol is available as online supplemental material, and any substantial amendments will be posted on ClinicalTrials.gov or made available on request from the corresponding author. The SPIRIT checklist refers to the full protocol.

## Methods and analysis

### Hypothesis and aim

We hypothesise that endometriosis surgery prior to ART in women with endometriosis stage III-IV results in improved reproductive outcomes compared with first-line ART. Our primary objective is to evaluate the cumulative live birth rate in women with stage III–IV endometriosis following ART, with or without prior surgical treatment. A full list of study objectives is presented in table 1.

**Table 1:** Primary and secondary objectives

Primary objective	
	Cumulative live birth rate within 3 years from first treatment (surgery or ART treatment)
Secondary objectives	
Key secondary objective	Quality of life and pain
	Cumulative pregnancy rate
	Time to pregnancy and live birth
	Spontaneous pregnancy rate
	Miscarriage rate and/or extrauterine pregnancies
	Reproductive outcomes per IVF cycle
	Rate of recurrent implantation failure, evaluated at the end of the study period
	Infections after oocyte pick-up
	Perioperative and 2 months postoperative complications classified by Clavien-Dindo and classic systems
	Healthcare costs
	Obstetrical outcomes and complications
	Progesterone levels on day of embryo transfer at first HRT frozen-thawed artificial embryo transfer cycle

ART, assisted reproductive technologies; HRT, hormone replacement therapy; IVF, in vitro fertilisation.

### Study design

This study is designed as a multicentre, prospective, open-label RCT. The trial will compare endometriosis surgery prior to ART with first-line ART in women with stage III-IV endometriosis and infertility, classified according to the American Association of Gynecological Laparoscopists (AAGL) endometriosis system.

### Study setting and recruitment

All women with stage III-IV AAGL endometriosis and infertility referred or eligible for advanced endometriosis surgery at the national highly specialised medical care services (Nationell Högspecialiserad Vård (NHV) centres) and/or their first fertility treatment such as in vitro fertilisation (IVF) or intracytoplasmic sperm injection (ICSI) will undergo screening for this trial.

All advanced endometriosis surgeries in Sweden are centralised to four NHV centres (Stockholm, Uppsala, Göteborg, Malmö). After oral and written informed consent, patients will be registered and randomised 1:1. Registration data will be entered into an electronic case report form (eCRF). Participant recruitment is planned from September 2025 until 2028. The required number of participants, based on power calculation, is 350.

Inclusion criteria are: women with severe endometriosis (AAGL stage III-IV) referred for or eligible to undergo their first ART treatment (IVF or ICSI) or their first endometriosis surgery; age 18–38 years; body mass index 18–35 kg/m^2^.

Exclusion criteria are: previous surgery for endometriosis (except diagnostic laparoscopy); previous IVF/ICSI cycles; haematosalpinx or hydrosalpinx; clear indication for organ saving surgery such as ureteric stenosis or intestinal subocclusion; suspicion of ovarian malignancy; submucosal fibroids of any size (according to the classification of International Federation of Gynaecology and Obstetrics (FIGO) 0–1) or intramural fibroids (FIGO 2–5) >4 cm; uterine malformations (U 1–6 according to the ESHRE classification); or ART treatment with donated oocytes.

The study will open for inclusion in Q3 2025 and has a planned 3-year inclusion period.

### Randomisation

After verification of eligibility, signed informed consent, participants will be randomly assigned to undergo either first-line ART or first-line surgery, followed by ART, by equal allocation (1:1). The randomisation procedure will be stratified for participating centre using a permuted block design. Randomisation will be performed at each site using the web-based instrument Research Electronic Data Capture (REDCap).

All inclusion criteria and no exclusion criteria must be met. One month before first treatment (IVF or surgery), criteria will be entered into the randomisation/registration application in REDCap. Access requires username and password; each investigator authorised to register patients will have a personal. If all criteria are met, patients are registered and the allocated study number is recorded in the medical file. Patients withdrawn from the study after randomisation but before treatment will be substituted by newly enrolled patients. Patients withdrawn from the study after receiving the first treatment will not be substituted.

### Study treatment

Surgery will be performed at one of four nationally specialised centres for advanced endometriosis surgery (Södersjukhuset, Stockholm; Sahlgrenska University Hospital, Gothenburg; Skåne University Hospital, Malmö/Lund; Uppsala University Hospital). Intraoperative AAGL staging, #ENZIAN classification and Endometriosis Fertility Index will be recorded in the study protocol.^16^ Surgery will follow the ESHRE recommendations for endometriomas and deep endometriosis.^17 18^ Procedures will be performed by experienced endometriosis surgeons, with multidisciplinary collaboration (colorectal surgeons and urologists) when required. Endometriomas will primarily be treated by gentle stripping of the cyst wall, with haemostasis achieved using bipolar coagulation or suturing if needed. Alternative techniques such as ethanol sclerotherapy, CO_₂_ laser, argon plasma coagulation or plasma jet may be applied depending on lesion characteristics and surgeon preference. Colorectal lesions will be managed by shaving, discoid excision or segmental resection depending on lesion depth and size.^19^

ART treatments will be carried out at Swedish reproductive units authorised to provide publicly funded fertility care. Most treatments are expected to take place at the university-affiliated reproductive clinics in Stockholm, Gothenburg, Skåne and Uppsala. For participants residing in other regions, local treatment will be possible to reduce the logistical and emotional burden of long-distance travel.

In Sweden, ART treatment costs for women under 40 years without children in the current relationship are covered by the tax-funded healthcare system, with up to three IVF/ICSI treatments, provided if considered medically appropriate and initiated before the woman turns 40.^20^

Further details on treatment regimens are provided in the full protocol (online supplemental material).

Time from randomisation to first treatment (surgery or ART cycle) should be minimised, ideally within 3 months and not exceeding 6 months. Patients undergoing surgery will need to delay ART treatment for 2–3 months to recover. Complications from either treatment (IVF or surgery) will be registered in the eCRF and managed according to routine clinical care.

### Study interventions

All women with stage III-IV AAGL endometriosis and infertility referred for, or eligible for, surgery and/or ART will undergo screening. The results will be documented in a screening log. After informed consent, patients will be registered and randomised, with data entered into the eCRF.

At enrolment, all patients will undergo transvaginal ultrasound, supplemented with MRI if required.

Participants randomised to first-line surgery will undergo the procedure at one of the four specialised centres. Following recovery, IVF/ICSI will be initiated according to the routine protocol at the reproductive units.

**Figure 1:**
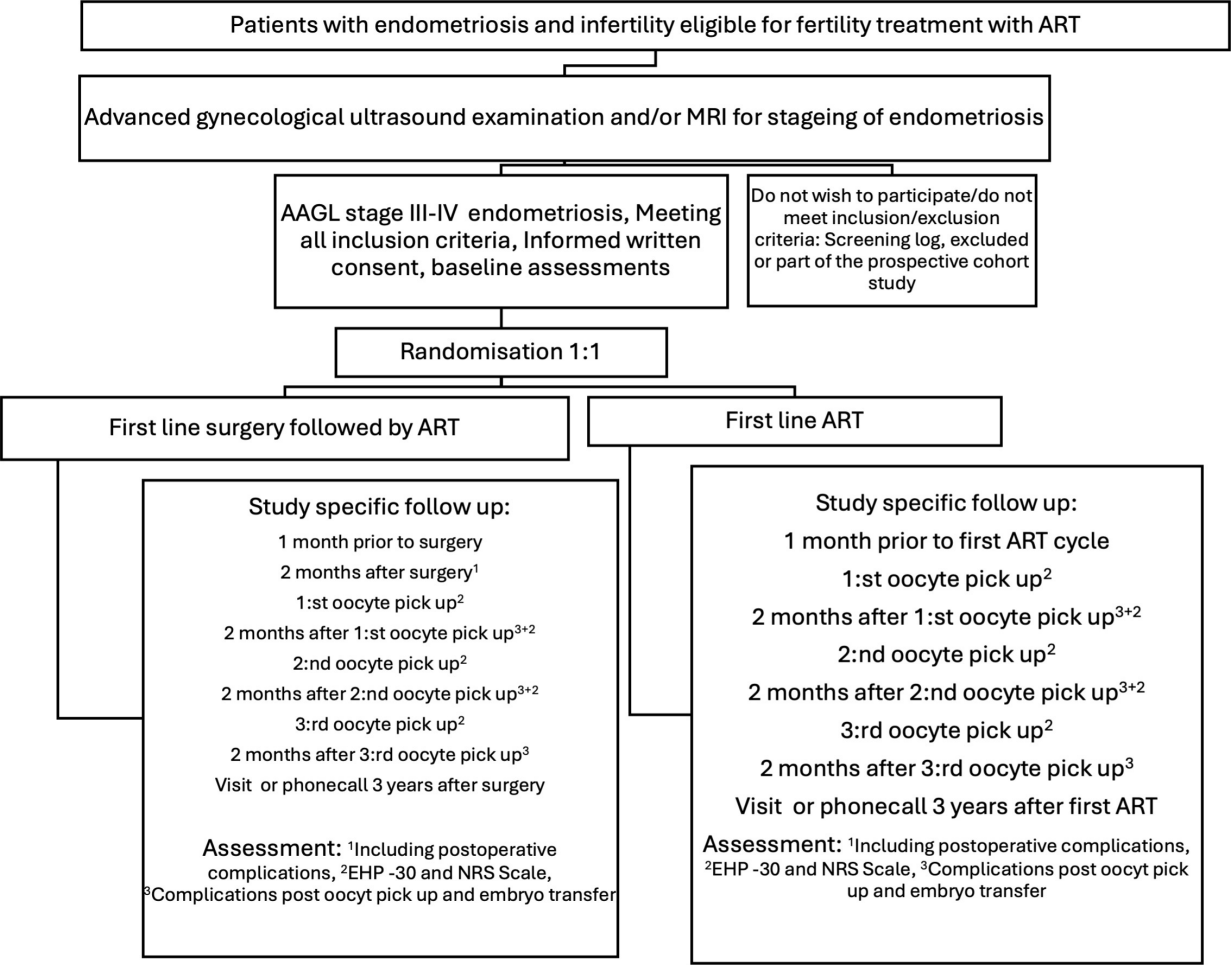
Study flowchart. AAGL, American Association of Gynecological Laparoscopists; ART, assisted reproductive technologies; EHP, Endometriosis Health Profile; NRS, Numeric Rating Scale.

Participants randomised to the control arm will proceed directly to IVF/ICSI without prior endometriosis surgery (figure 1).

Data regarding baseline characteristics, potential covariates and outcomes will be collected throughout the study period from medical records and validated questionnaires.

A full list of study procedures and interventions at each study visit is available in online supplemental table S1 and in the full protocol (online supplemental material).

### Quality assurance of surgery and fertility treatment

All participating centres are nationally designated highly specialised endometriosis centres, approved by the Swedish National Board of Health and Welfare following a formal application and quality assessment process. This includes documentation of institutional experience, annual surgical volume, infrastructure and the ability to perform standardised endometriosis staging.

All participating surgeons are approved by the coordinating investigators and steering committee and must have prior experience of at least 20 advanced endometriosis surgeries. Surgeon experience and annual caseload are documented prior to trial initiation, and only approved surgeons are permitted to act as lead surgeons. For each surgical procedure, data on surgeon volume and centre volume are prospectively collected.

Randomisation is stratified by centre to account for potential centre-related differences in operative volume and routine. Surgical and fertility outcomes are continuously monitored, and centres or individual surgeons may be temporarily or permanently suspended from further recruitment in case of quality concerns, following consultation with the Data Safety Monitoring Board.

### Outcome measures and endpoints

The primary outcome is the cumulative live birth rate (CLBR) after up to three complete ART cycles, defined as the proportion of participants achieving at least one delivery of a live born infant beyond 22 weeks of gestation within 3 years following the initiation of

the allocated fertility treatment strategy. Live births resulting from spontaneous conception occurring after treatment initiation are included in the CLBR.

Participants who achieve a spontaneous pregnancy before initiation of the allocated treatment are not included in the primary analysis and will be replaced to preserve the integrity of the randomised comparison.

Secondary outcomes include: cumulative pregnancy rate, miscarriage and ectopic pregnancy, time to live birth or clinical pregnancy, spontaneous pregnancy rate, reproductive outcomes per IVF cycle (oocytes retrieved, fertilisation rate, fresh embryo transfer characteristics, number of blastocysts cryopreserved, pregnancy and live birth outcomes per embryo transfer and per initiated IVF/ICSI cycle), recurrent implantation failure, infections after oocyte retrieval, perioperative and postoperative surgical complications (classic and Clavien–Dindo classifications), health-related quality of life and pain (Endometriosis Health Profile-30 (EHP-30), Euro-Qol 5-Dimension questionnaire (EQ-5D) and Numeric Rating Scale (NRS)), cost-effectiveness (including quality-adjusted life years (QALYs)), pregnancy outcomes, obstetric and postpartum outcomes and serum progesterone levels on the day of embryo transfer in hormone-replacement therapy frozen embryo transfer (HRT-FET) cycles.

A full list of secondary endpoints and detailed definitions is provided in the study protocol (online supplemental material) and is consistent with the trial registry.

### End of study

The study will end when all patients enrolled in the trial have been followed for 3 years after first treatment, withdrawn consent or been lost to follow-up. Data from participants lost to follow-up will be included in the final analysis. The Trial Steering Committee may end enrolment at any time if it is deemed in the best interests of patients.

### Data management and monitoring

Study data will be collected from medical records, ultrasound and surgical reports, IVF documentation and blood test results, with (online supplemental information) obtained from participants when required. Quality-of-life questionnaires will be distributed through REDCap, which also serves as the eCRF. Registry data (eg, Q-IVF, Gynop, Swedish Medical Birth Register) will be linked using the unique Swedish personal identification number for long-term follow-up.

Source data will be patients’ medical records, with information transcribed into the eCRF. Data will be managed and stored by the Clinical Trials Office (CTO), Karolinska University Hospital, in compliance with Good Clinical Practice and General Data Protection Regulation (GDPR). All information will be handled confidentially and participants will be identified only by coded numbers. Study records and essential documents will be stored securely for at least 10 years after study completion, with access restricted to the investigators and authorised personnel.

Trial monitoring will be overseen by the CTO, Karolinska University Hospital, with verification of eCRF entries against source data. A monitor from the CTO team will be responsible for coordinating the monitoring activities of the study and ensuring adherence to GCP guidelines. The monitor will have access to medical records and source data after a secrecy agreement has been signed by the responsible party at the site as well as by the monitor. The investigator must ensure that all source documents are accessible for monitoring and other quality control activities.

### Sample size calculation and statistical analyses

A sample size calculation for a superiority trial was conducted to compare the CLBR between two treatment groups. The calculation was based on a two-sided comparison of proportions, assuming a type I error (α) of 0.05 and 80% power (1 – β).

Expected success rates were informed by two observational comparative studies,^12 13^ which reported a CLBR of approximately 55% after three IVF cycles in the first-line ART group and 71% in the group receiving surgery followed by ART. Based on these proportions, the estimated total required sample size was approximately 284 participants. To account for an anticipated dropout rate of 20%, the final planned sample size is 350 participants.

All primary analyses will be performed according to the intention-to-treat principle. An interim analysis is planned at the halfway point of recruitment to primarily address recruitment feasibility and participant safety, including comparison of complication rates between study centres. The primary outcome, CLBR, will first be assessed through a simple comparison of proportions between the two treatment groups, presenting crude relative risks with 95% CIs.

Subgroups of special interest will include type of ART (IVF or ICSI), age (<35 vs ≥35 years), ovarian reserve (Anti-Müllerian Hormone (AMH)<0.5; 0.5–3.5; >3.5 ng/mL), adnexal surgery (yes/no) and surgery for deep endometriosis in the anterior (yes/no) and posterior (yes/no) compartment, defined using Swedish procedure classification codes.

Secondary and exploratory analyses will include multivariable modelling using log-binomial regression to investigate the association between treatment and CLBR while adjusting for potential confounding factors. Covariates will be selected based on clinical relevance and a directed acyclic graph.

Time-to-event outcomes (eg, time to live birth or clinical pregnancy) will be analysed using Kaplan-Meier survival curves and compared using stratified log-rank tests. A Cox proportional hazards model will be applied for adjusted analysis of time-to-event outcomes.

Health economic evaluation will include calculation of incremental cost-effectiveness ratios, using QALYs as the effect measure. A probabilistic sensitivity analysis will be performed to evaluate uncertainty in the economic model.

Adverse events will be categorised and summarised by treatment group, with severity and suspected relation to treatment noted. Missing data will be handled using appropriate methods, including multiple imputation where appropriate.

### Safety and adverse events

Participants in the study may face several physical risks. Women randomised to surgery before IVF are at risk of complications associated with endometriosis surgery, such as bleeding, bowel or bladder injury and postoperative infection. ART treatment, with or without prior surgery, also carries risks, including infection and pain during oocyte retrieval. The Swedish patient injury insurance will apply as usual.

Since both treatment strategies are already in clinical use, randomisation is considered both justified and feasible. Adverse events will be categorised and summarised by treatment group, with severity and suspected relation to treatment noted. An independent data safety and monitoring board (DSMB) will conduct one interim analysis 1.5 years after the randomisation of the first patient or when 175 patients have completed surgery or first IVF treatment, whichever occurs first. The analysis will primarily address recruitment feasibility and participant safety, including comparison of complication rates between study centres. The DSMB will monitor adverse events in both arms. A substantially higher-than-expected complication rate in either arm, major discrepancies between centres or other safety concerns may prompt the DSMB to recommend protocol modifications or early termination. Recommendations from the DSMB will exclusively be communicated to the Trial Steering Committee. Further details regarding the DSMB can be found in the DSMB charter (online supplemental material 1).

Additionally, there may be psychological risks related to the emotional stress of undergoing medical procedures, the uncertainty of treatment outcomes and the emotionally charged nature of fertility-related decisions. All the participants will have access to psychological support or counselling through their fertility clinic or endometriosis unit, as a part of the standard care. Risks related to the collection and storage of sensitive medical data will be mitigated through strict data protection measures.

### Patient and public involvement

The Swedish Endometriosis Patient Association has been involved in planning the Endo-SOFT trial and has encouraged the study group, since the research question is important to many endometriosis patients. We confirmed through patient feedback that most women with endometriosis are willing to participate in research projects of this kind. A qualitative interview study is currently being conducted to explore the patients’ thoughts, concerns, fears and hopes regarding their endometriosis, infertility and potential surgery. The preliminary results from this qualitative study show an overwhelming willingness to contribute to endometriosis research.

## Ethics and dissemination

The study protocol, patient information and informed consent are approved by Swedish Ethical Review Authority (dnr: 2024–04293-01, approved 7 October 2024, amendment, dnr: 2025–03699-02, approved 17 June 2025).

This study will be designed, conducted and reported in accordance with the study protocol; the principles outlined in the Declaration of Helsinki, and/or relevant Swedish or National laws and regulations, whichever offers the highest level of protection for the patient. Participants will be clearly informed that the data collected in the study will adhere to the GDPR (EU 2016/679), ensuring that no subject participating in the study will be identifiable. Women participating in the study will receive treatment in alignment with the international guidelines on GCP as defined by the European Parliament (EG596/200).

Endometriosis is a highly prevalent condition (affecting around 22% of infertile women), and this research project will have implications for many patients, regardless of the results. The results of the Endo-SOFT trial will be published in open access peer-reviewed scientific journal. They are likely to influence guidelines and care for women with severe endometriosis and infertility and may also provide insights into the management of women with less severe disease. Other aspects, such as the concept of centralisation of advanced surgery, will also be evaluated.

### Data availability statement

Data from this study will be stored and managed by the CTO at Karolinska University Hospital. Individual participant data will not be publicly available but may be shared on reasonable request, subject to applicable ethical and legal regulations.

## Supplementary material

10.1136/bmjopen-2025-111928 online supplemental file 1

10.1136/bmjopen-2025-111928 online supplemental file 2
