# Supplementary material for: Endo-SOFT: study protocol for a national multicentre randomised controlled trial – first-line surgery versus first-line assisted reproductive technologies in patients with advanced endometriosis
Source: BMJ Open. 2026 Mar 25;16(3):e111928. doi: 10.1136/bmjopen-2025-111928 (PMC13034312; doi:10.1136/bmjopen-2025-111928)
Supplement: online supplemental file 1 [file bmjopen-16-3-s001.docx]

**THE ENDO-SOFT TRIAL**

First line surgery or first line fertility treatment using assisted reproductive technologies in patients with advanced endometriosis: A national multicenter randomized-controlled trial.

**Protocol identification number:**

**Coordinating and principal Investigator**

Malin Brunes MD, PhD

Department of Obstetrics and Gynecology, Södersjukhuset, Stockholm, Sweden

**Principal Investigator**

Anna Marklund MD, PhD, Department of Reproductive Medicine, Karolinska University Hospital, Huddinge, Stockholm, Sweden

**Co-investigators**

Maria Forslund MD, PhD, Associate professor

Christine Asciutto MD, PhD

Ligita Jokubkiene MD, PhD, Associate professor

Evangelia Elenis MD, PhD, Associate professor

Jynfiaf Francis MD

Hanna Åmark MD, PhD

**Date:**

**Clinical Trials.gov:**

**Authors:** Malin Brunes, Anna Marklund, Maria Forslund, Christine Asciutto, Ligita Jokubkiene, Evangelia Elenis, Jynfiaf Francis

TABLE OF CONTENTS

[CONTACT INFORMATION 3](#_Toc169963693)

[SYNOPSIS 6](#_Toc169963694)

[LIST OF ABBREVIATIONS 9](#_Toc169963695)

[WHY THIS TRIAL IS NEEDED 11](#_Toc169963696)

[STUDY OBJECTIVES 13](#_Toc169963697)

[STUDY DESIGN 20](#_Toc169963698)

[STUDY FLOW CHART 21](#_Toc169963699)

[SCHEDULE OF EVENTS TABLE 22](#_Toc169963700)

[TIMELINE 23](#_Toc169963701)

[STUDY ENROLLMENT 24](#_Toc169963702)

[STUDY TREATMENT 26](#_Toc169963703)

[QUALITY ASSURANCE OF SURGERY AND FERTILITY TREATMENT 28](#_Toc169963704)

[PATIENT REPORTED OUTCOMES (PROMS) 29](#_Toc169963705)

[HEALTH ECONOMICS 31](#_Toc169963706)

[ADENOMYOSIS 31](#_Toc169963707)

[OBSTETRICAL OUTCOMES 32](#_Toc169963708)

[PROSPECTIVE COHORT STUDY 33](#_Toc169963709)

[LONG-TERM FOLLOW-UP 33](#_Toc169963710)

[THE REPRODUCTIVE OUTCOMES RELATED TO P-PROGSTERONE ON THE DAY OF TRANSFER OF THE CRYOPRESERVED EMBRYO WITH HORMONE REPLACEMENT THERAPY-CYCLES 33](#_Toc169963711)

[STATISTICAL CONSIDERATIONS 34](#_Toc169963712)

[INTERIM ANALYSIS AND STOPPING RULES 38](#_Toc169963713)

[ETHICAL CONSIDERATIONS 38](#_Toc169963714)

[GENDER PERSPECTIVE 40](#_Toc169963715)

[SIGNIFICANCE OF STUDY 40](#_Toc169963716)

[ADMINISTRATIVE CONSIDERATIONS 41](#_Toc169963717)

[DATA MANAGEMENT AND QUALITY CONTROL 43](#_Toc169963718)

[REFERENCES 46](#_Toc169963719)

[APPENDIX 50](#_Toc169963720)

# CONTACT INFORMATION

| Coordinating investigator | Malin Brunes  Södersjukhuset  118 83 Stockholm, Sweden  malin.brunes@regionstockholm.se  +86812362729 |
| --- | --- |
| Co-investigator | **Anna Marklund**  Karolinska University Hospital Huddinge  17176 Stockholm, Sweden  anna.marklund@regionstockholm.se  +46 737273133 |
| Co-investigators | **Maria Forslund** MD, PhD, Associate professor Sahlgrenska University Hospital, Gothenburg  **Christine Asciutto** MD, PhD, Uppsala akademiska Hospital  **Ligita Jokubkiene** MD, PhD, Associate professor, Skåne university hospital, Malmö  **Evangelia Elenis** MD, PhD, Associate professor Uppsala University Hospital, Uppsala  **Jynfiaf Francis** MD, Sahlgrenska University Hospital, Gothenburg |
| Doctoral student | **Julia Wangberg** MD, Sahlgrenska University Hospital, Gothenburg  **Kristin Wennmo Zuk** MD, Södersjukhuset, Stockholm |
| Professors | **Kenny Rodriguez Wallberg** Karolinska  University Hospital, Huddinge, Stockholm  **Karin Sundfeldt** Sahlgrenska University Hospital, Gothenburg |
| Trial Steering Committee | **Malin Brunes**, MD, PhD,  Södersjukhuset, Stockholm  **Anna Marklund**, MD, PhD, Karolinska  University Hospital, Huddinge, Stockholm  **Maria Forslund** MD, PhD, Associate professor, Sahlgrenska University Hospital, Gothenburg  **Christine Asciutto** MD, PhD, Uppsala University Hospital, Uppsala  **Ligita Jokubkiene** MD, PhD, Associate professor, Department of obstetrics and gynecology, Skåne university hospital, Malmö |
| Statistician | **Anna Olofsson**, Biostatistician, Institute of Environmental Medicine, Karolinska Institutet |
| Sub-Committee Quality of Life | **Maria Forslund** MD, PhD, Associate professor Sahlgrenska University Hospital, Gothenburg |
| Sub-Committee Health Economy | **Maria Forslund** MD, PhD, Associate professor, Sahlgrenska University Hospital, Gothenburg |
| Sub-Committee Adenomyosis | **Anna Marklund** MD, PhD, Karolinska  University Hospital, Huddinge, Stockholm |
| Sub-Committee Obstetrical Outcomes | **Ligita Jokubkiene** MD, PhD, Associate professor, Department of obstetrics and gynecology, Skåne university hospital, Malmö  **Hanna Åmark** MD, PhD, Södersjukhuset, Stockholm |
| Sub-Committee Prospective cohort study and Long-term follow up | **Malin Brunes**, MD, PhD,  Södersjukhuset, Stockholm,  Sweden, Department of Clinical Science and Education, Södersjukhuset, Karolinska Institutet  **Anna Marklund**, MD, PhD, Karolinska  University Hospital, Huddinge, Stockholm, Sweden  **Ligita Jokubkiene** MD, PhD, Associate professor, Department of Clinical sciences, Malmö, Lund University/Department of obstetrics and gynecology, Skåne university hospital, Malmö |
| Sub-Committee P-Progesterone and reproductive outcome | **Jynfiaf Francis** MD, Sahlgrenska University Hospital, Gothenburg |
| Data safety and monitoring board  (DSMB  ) | **Sophia Brismar Wendel**, Associate professor, Department of Clinical scienses, Danderyd Hospital, Karolinska Institutet  **George Dimakopopulos**, Biostatistician, Science and Technology Park of Epirus, Ioannina  Associate professor (to be decided) |
| Trial Coordinating Centre | **Department of Obstetrics and Gynecology,** Södersjukhuset, Stockholm, Sweden |
| Data manager and monitor | Clinical Trials Office (CTO), Center for Clinical Cancer Studies, Karolinska University Hospital |

# SYNOPSIS

| **Protocol Title** | First line surgery versus first line fertility treatment using assisted reproductive technologies in patients with advanced endometriosis: A national multicenter randomized controlled trial. |
| --- | --- |
| **Indication** | Patients with endometriosis AAGL stage III-IV eligible for their first fertility treatment |
| **Primary objective** | To assess whether surgery conducted prior to treatment with assisted reproductive technologies (ART) in patients with endometriosis AAGL stage III-IV increases cumulative live birth (CLBR) as compared to first line ART. |
| **Secondary**  **objectives** | To assess if surgery prior to ART treatments in women with endometriosis AAGL stage III-IV result in improved, Cumulative Pregnancy Rate (CPR) and lower miscarriage rate as compared to first line ART.  To compare time to CLBR and CPR  To evaluate intra- and postoperative complications after oocyte pick-up and surgery.  To compare quality of life and pain at follow up and do a health economic evaluation.  To examine obstetrical outcomes of the resulting pregnancies  To investigate differences in plasma progesterone levels (P-progesterone) at the day of frozen embryo transfer in hormone replacement therapy (HRT) cycles |
| **Study Design** | Randomized controlled trial |
| **Planned sample**  **Size** | 350 |
| **Inclusion criteria** | - Age 18 - 38 years. - Endometriosis AAGL stage III-IV - Eligible for ART-treatment such as in vitro fertilization (IVF) or intracytoplasmic sperm injection (ICSI) - Body mass index 18 – 35 kg/m^2^ |
| **Exclusion criteria** | - Previous surgery for endometriosis except diagnostic laparoscopy. - Previous IVF/ICSI cycles - Hemato- /hydrosalpinx - Clear indication for surgery such as ureteral stenosis or intestinal sub-occlusive symptoms - Suspicion of ovarian malignancy - Submucosal fibroids of any size (FIGO 0-1) or intramural fibroids (FIGO 2-5) > 4 cm - Uterine malformations (U 1-6 according to ESHRE classification) - Contraindications to surgery - ART treatment with donated oocytes |
| **Primary outcome** | Cumulative live-birth rate (CLBR) within three years from first treatment (Surgery or ART) |
| **Secondary**  **outcomes** | - Cumulative Pregnancy Rate (CPR) - Time to pregnancy and live birth - Miscarriage rate - Reproductive outcomes per IVF cycle, - Rate of recurrent implantation failure (RIF) - Intra- and postoperative complications after surgery - Infections after oocyte pick-up - Quality of life - Pain - Health costs - Obstetrical outcomes and complications - P-progesterone at the day of frozen embryo transfer in HRT cycles |
| **Standard treatment** | First line ART |
| **Experimental**  **Treatment** | First line surgery followed by ART |
| **Duration of study**  **including follow up** | 6 years (inclusion 3 years and follow-up 3 years) |

# LIST OF ABBREVIATIONS

AAGL American Association of Gynecologic Laparoscopists

ADL activity of daily life

AMH anti-mullerian hormone

ART assisted reproductive technologies

ASA-class The American Society of Anesthesiologists physical status classification system

BMI body mass index

COS controlled ovarian stimulation

CPR cumulative pregnancy rate

CLBR cumulative live birth rate

DSMB Data Safety Monitoring Board

DE deep endometriosis

DET double embryo transfer

eCRF electronic Case Report Form

EHP-30 Endometriosis Health Profile

ESHRE European Society of Human Reproduction and Embryology

ET embryo tranfer

FET frozen embryo transfer

rFSH recombinant follicle stimulating hormone

FIGO The International Federation of Gynecology and Obstetrics

GCP Good Clinical Practice

GnRH Gonadotropin hormone-releasing hormone

GV – germinal vesicle stage oocyte

hMG human menopausal gonadotropin

HRQoL Health related quality of life

HRT – Hormone Replacement Therapy

ICSI intracytoplasmic sperm injection

IDEA International Deep Endometriosis Analysis group

IVF in vitro fertilization

KVÅ klassifikation av vårdåtgärder

LBR live birth rate

MI metaphase I oocyte

MII metaphase II (mature) oocyte

MFR Swedish Medical Birth Register

MUSA Morphological Uterus Sonographic Assessment

MRI magnetic resonance imaging

NC natural cycle

NRS Numeric Rating Scale

OHSS ovarian hyperstimulation syndrome

OPU oocyte pick up

PR pregnancy rate

QALYs quality-adjusted life years

Q-IVF National Quality Registry for Assisted Reproduction

rASRM revised American Society for Reproductive Medicine classification of endometriosis

RCTs randomized controlled trials

RedCap Research Electronic Data Capture system

RIF Recurrent Implantation Failure

SET single embryo transfer

# WHY THIS TRIAL IS NEEDED

BACKGROUND

Endometriosis

Endometriosis is a prevalent condition affecting 10% of female population of reproductive age, leading to pain and subfertility (1). An earlier systematic literature review and meta-analysis suggests that women with advanced endometriosis (revised American Society for Reproductive Medicine classification of endometriosis (rASRM) stage III-IV) have a lower likelihood of achieving clinical pregnancy/live birth compared to women without endometriosis, while this difference has not been observed for mild disease (stage I-II) (2).

Gynecological Ultrasound and staging

The prevalence of endometrioma and deep endometriosis found through systematic transvaginal ultrasound on women referred for ART treatment in Sweden has been estimated at 21.8% (3). Additionally, 75.8% of those with endometriosis were unaware of their condition prior to diagnosis (3). In recent years, two staging systems, namely the American Association of Gynecologic Laparoscopists (AAGL) Endometriosis classification (4, 5) and #ENZIAN (6) have gained prominence owing to their accuracy when juxtaposed with surgical findings.

Surgical Treatment

Women with endometriosis seem to experience pain relief after bowel surgery due to endometriosis (7, 8). Consensus from the European Society of Human Reproduction and Embryology (ESHRE) indicates that surgery can be performed prior to treatment with assisted reproductive technologies (ART) (7). Additionally, the number of spontaneous pregnancies is high after surgery on patients with deep endometriosis (10). Furthermore, in patients with endometriosis stage I-II or in infertile patients with endometriomas, surgery can in some cases be considered to improve fertility (7-9). However, the risk of severe complications in patients undergoing rectal surgery is up to 10% including anastomosis leakage and fistulas (10, 11).

Fertility treatment

An earlier meta-analysis found that the number of oocytes and fertilization rates are lower in women with endometriosis as compared to those without, although not necessarily impacting the live birth rate (12). Still, some previous studies have shown lower likelihood of live birth and pregnancy after in vitro fertilization (IVF) in women with endometriosis compared to women with unexplained or tubal infertility (13, 14). Even though there is some controversy when it comes to the evidence of association between endometriosis and adverse reproductive outcome after IVF, there is biological plausibility for this type of association, especially in women with advanced endometriosis (chronic inflammation affecting folliculogenesis, technical difficulties at oocyte retrieval due to endometriomas or adhesions, increased risk of pelvic infection after oocyte pick-up) (15, 16).

RATIONALE FOR STUDY

Only a few comparative retrospective studies on first-line surgery versus first-line ART have been performed, with no available randomized controlled trials (RCTs) (17). In two retrospective register studies, first-line surgery in patients with deep endometriosis (DE) in the anterior compartment or colorectal DE showed improved live birth rates (LBR) and pregnancy rates (PR) compared to first-line ART (18, 19). An important limitation in the previous studies on patients with endometriosis has been the challenge of staging and characterizing the disease consistently using preoperative imaging techniques. Currently, the decision regarding the choice between surgery and IVF is collaborative and individualized, considering various factors such as the patient's medical and surgical history, presence of pain symptoms, age, results of ovarian reserve testing, and semen analysis. Lack of randomized RCTs or at least robust prospective cohort studies comparing these two approaches is noteworthy. Hence, there is an obvious need for a randomized controlled trial to address the impact of surgery prior to ART on the reproductive outcomes in women with endometriosis AAGL stage III-IV, to help patients and health-care workers with decision-making, with the overall aim to improve patient-outcomes.

# STUDY OBJECTIVES

HYPOTHESIS

First line surgery (prior to ART treatment) in women with endometriosis AAGL stage III-IV will result in improved cumulative live birth rate (CLBR) as compared to first line ART.

PRIMARY OBJECTIVE

To assess whether surgery conducted prior to ART in women with endometriosis AAGL stage III-IV increases chances of CLBR as compared to first line ART.

.

SECONDARY OBJECTIVES

To evaluate whether surgery prior to ART treatments in women with endometriosis AAGL stage III-IV will result in improved cumulative pregnancy rate (CPR) and lower miscarriage rate as compared to first line ART.

To compare whether surgery prior to ART treatments in women with endometriosis AAGL stage III-IV will result in shorter time to live birth or pregnancy as compared to first line ART.

To investigate if surgery prior to ART treatments in women with endometriosis AAGL stage III-IV will result in better reproductive outcomes per IVF cycle and a lower rate of recurrent implantation failure (RIF).

To assess if surgery prior to ART treatments in women with endometriosis AAGL stage III-IV will result in lower rate of infections after oocyte pick-up requiring treatment with antibiotics as compared to first line ART?

To evaluate if surgery prior to ART treatments in women with endometriosis AAGL stage III-IV result in higher Quality of life and less pain measured with Endometriosis Health Profile (EHP-30) and Numeric Rating Scale (NRS) before ART, i) at the time of oocyte retrieval, ii) two months after ART and iii) at the three-year follow-up visit as compared to first line ART.

To explore whether surgery prior to ART treatments in women with endometriosis AAGL stage III-IV will be cost effective compared with first line ART?

To examine if surgery prior to ART treatments in women with endometriosis AAGL stage III-IV will result in reduced pregnancy and delivery complications, as well as fewer complications within 8 weeks postpartum, compared to first line ART.

To investigate whether serum progesterone level on the day of frozen embryotransfer in hormone replacement cycles in patients with severe endometriosis is associated with reproductive outcomes (LBR, pregnancy rate, miscarriage rate) and to determine if there is any difference in p-progesterone level between the two different study groups first line ART vs first line surgery in women with endometriosis AAGL stage III-IV?

PRIMARY OUTCOME MEASURE

Cumulative live-birth rate within three years from first treatment (surgery or ART treatment).

Definition of primary outcome measure

CLBR is defined as any live births from first treatment until end of follow up (three years from first ART treatment or surgery or drop out from the study, whichever comes first).

Primary outcome will be calculated considering also the following pre-specified subgroups: study center,  type of ART-treatment (IVF or ICSI), age (< and > 35 years), ovarian reserve ( anti-mullerian hormone (AMH) <0.5ng/L, 0.5-3.5 ng/L and AMH >3.5ng/L ) (35) adnexal surgery (yes/no), surgery on deep endometriosis anterior compartment (yes/no) or deep endometriosis posterior compartment (yes/no) (defined by Classification of surgery, “Klassifikation av vårdåtgärder” (KVÅ codes), see appendix).

SECONDARY OUTCOME MEASURE

- Cumulative Pregnancy Rate (CPR)
- Time to pregnancy and live birth
- Spontaneous Pregnancy Rate
- Miscarriage rate and/or extrauterine pregnancies
- Reproductive outcomes per IVF cycle
- Rate of recurrent implantation failure (RIF), (20) evaluated at the end of the study period
- Infections after oocyte pick-up
- Quality of life and pain
- Peri- and two months postoperative complications classified by Clavien-Dindo and Classic systems
- Health care costs
- Obstetrical outcomes and complications
- P-progesterone levels on day of embryo transfer at first HRT frozen-thawed artificial embryo transfer cycle

Definition of secondary outcome measures

Cumulative Pregnancy Rate (CPR) is defined as all pregnancies confirmed with ultrasound, from first treatment (surgery or ART) until three years of follow up.

Time to pregnancy is defined as time from first treatment (surgery or ART) to first pregnancy confirmed with ultrasound. Time to live birth defined as time from first treatment (surgery or ART) to first livebirth.

Spontaneous pregnancy rate is defined as any spontaneous pregnancy occurring between first treatment and end of follow-up.

Miscarriage rate defined as frequency and proportion of spontaneous loss of any clinical and biochemical pregnancy before week 22 and/or extrauterine pregnancy defined as a pregnancy not located in the uterine cavity, occurring between first treatment and end of follow-up.

Reproductive outcome per IVF/ICSI-cycle: number of oocytes retrieved (mature and/or total), fertilization rate, Fresh embryo transfer (ET) (day 2, day 3, day 5), number of blastocysts cryopreserved, pregnancy rate per ET, live birth rate per ET, miscarriage rate per ET, cumulative pregnancy rate per cycle, cumulative miscarriage rate per cycle, cumulative live birth rate per cycle.

RIF (recurrent implantation failure) evaluated at the end of the study period. RIF is defined here as no pregnancy after three ETs in women <35 years and four ETs in women 35-39 years old (20).

Infections after oocyte pick-up will be registered yes/no and further classified as requiring i) treatment with antibiotics, ii) readmission or iii) surgical intervention.

Health related quality of life (HRQoL) and pain will be assessed by questionnaires, Endometriosis Health Profile (EHP-30), EQ5D and Numeric Rating Scale (NRS), completed by study participants preferably as electronical patient reported outcome measures or during the clinic visit (by manually filling in forms, by accessible computer or by mail). The assessments are performed maximum one month before the first intervention, at the time of oocyte retrieval, two months after ART or surgery and at three-year follow-up.

Intraoperative complications will be classified by Classic systems (21).

Two months postoperative complications will be defined according to the Clavien-Dindo (22) system.

Health care costs from randomization to end of trial: Direct costs will be used by assessing internal accounting and billing systems within the hospitals, cost per patient (CPP). We will also measure the quality-adjusted life years (QALYs) gained with the intervention and use this to undertake a cost-utility analysis. The QALY calculations will be based on fertility status measures for trial participants, with valuations of changes in fertility status and quality of life based on the EQ-5D.

Obstetrical outcomes and complications defined as:

- pregnancy- related complications: premature delivery, preeclampsia, gestational diabetes, placenta previa, placenta ablation, placenta spectrum disorders
- delivery-related complications: emergency caesarean section, pelvic floor trauma, delivery related bleeding, manual placenta removal
- postpartum complications (defined as within 8 weeks postpartum): infections (endometritis, pelvic inflammatory disease, urinary tract infection, wound infection, sepsis), remaining conceptual tissue with and without surgical intervention, use of analgesics

COVARIATES

Potential covariates will be collected from patients’ medical records.

*Baseline characteristics:*

- Age (continuous and dichotomized as < and > 35 years)
- Infertility duration (months)
- Infertility due to social factors (yes/no)
- Infertility due to pain from endometriosis (yes/no)
- Infertility due to male factor (yes/no)
- Smoking (yes/no)
- Snuffing (yes/no)
- BMI (continuous)
- Hormonal endometriosis treatment (yes/no, if yes subtype of drug, duration of hormonal treatment prior to first surgery or ART)
- Parity (0, 1-2 or >2)
- previous caesarian sections (yes/no)
- previous abdominal surgery (yes/no)
- previous adnexal surgery (yes/no)
- previous uterine or cervical surgery (yes/no, type of surgery)
- smoking habits (yes/no)
- ovarian reserve (AMH<0.5ng/L, 0.5-3.5 ng/L and AMH >3.5ng/L)
- myoma (yes/no)
- time from randomization to first oocyte pick-up or surgery (depending on which treatment arm the patient is randomized to)
- ASA-class (1-2 or 3-4)
- Other diseases affecting the outcome: hypothyroidism, diabetes, bleeding disorders or thrombogenic diseases, hypertension, hyperprolactinemia

*Imaging specific data (Ultrasound MRI)*:

- #ENZIAN-score
- AAGL-score
- Adenomyosis (yes/no)
- antral follicle count (AFC)

*Perioperative data:*

- #ENZIAN score
- AAGL score
- EFI score
- Surgical procedure defined by KVÅ- codes: adnexal surgery (yes/no), surgery on deep endometriosis anterior compartment (yes/no) or deep endometriosis posterior compartment (yes/no)
- Colorectal surgery (yes/no, if yes rectal shaving, discoid resection or segmental resection)
- Urological surgery (yes/no, if yes bladder resection, reimplantation of ureter, end to end anastomose of ureter, shaving of ureter)
- Perioperative data: estimated blood loss, operative time, conversion rate
- Annual surgeon volume (number of advanced endometriosis surgeries per year)
- Cumulative surgeon volume (number of advance endometriosis surgeries in total at surgery)
- Consultant surgeon (yes/no, if yes which specialty)
- Reoperation (yes/no) until 2 months post-surgery
- Readmissions (yes/no) until 2 months post-surgery
- Hospital stays (days)
- Time to normal activity of daily life (ADL) (days)

*Assisted reproductive technologies data (for each IVF/ICSI-cycle, max 3):*

- Complication – yes/no
- Type of complication (infection, ovarian hyperstimulation syndrome (OHSS), bleeding)
- Time from surgery to first oocyte pick-up
- Type of IVF protocols (GnRH agonist protocol/GnRH antagonist protocol)
- Total dose/mean daily dose of follicle stimulating hormone (rFSH) /human menopausal gonadotropin (hMG)
- Duration of IVF stimulation (days)
- Cancelled cycle (before or after oocyte pick up (OPU)) (excluding freeze all cycles)
- Reason of cancelled cycle
- Type of ovulation triggering [GnRH agonist (Gonapeptyl/Suprecur/Suprefact/Synarela) / Ovitrelle]
- Date of OPU
- Freeze all cycle (yes/no) and reason of that
- Number of retrieved oocytes (GV/MI/MII)
- Performed fertilisation method (ICSI/IVF)
- Sperm – fresh vs frozen
- Donor sperm – yes/no
- Origin of sperm – ejaculated/ epidydimal / testicular
- Oocytes – fresh vs frozen
- Number of inseminated oocytes
- Number of fertilised oocytes
- Number of good quality embryos defined according to Gardner criteria as AA, AB and BB for blastocysts (23) or top and good quality based on the number of blastomeres, the fragmentation rate and the multinucleation of blastomeres for cleavage stage embryos (24).
- Number of frozen blastocysts (D5 or D6)
- Date for fresh embryo transfer
- Embryo age at fresh embryo transfer (D2, D3, D4, D5 or D6)
- Number of embryos transferred (SET/DET)
- Type of embryo transfer (Fresh/Frozen)
- Type of protocols for Frozen-thawed ET (Natural Cycle (NC), HRT, Letrozol-stimulated, gonadotropinstimulated)
- Use of GnRH downregulation in Frozen-thawed HRT cycle (yes/no, number of days)
- Total estrogen dose and type of estrogen preparations in HRT FET-cycle
- Endometrial thickness prior to embryo transfer in HRT-FET
- Cycle day of endometrial thickness measurement in HRT-FET
- P progesterone level on the day of ET in HRT-FET
- Date of frozen ET
- Urinary pregnancy test (positive/negative)
- Gestational age at pregnancy ultrasound
- Fetal heartbeat at ultrasound (present/absent)
- Number of foetuses
- Number of gestational sacs
- Result foetus 1 (miscarriage, abortion, ectopic pregnancy, delivery)
- Date for delivery / end of pregnancy foetus 1
- Result foetus 2 (miscarriage, abortion, ectopic pregnancy, delivery)
- Date for delivery / end of pregnancy foetus 2
- Result foetus 3 (miscarriage, abortion, ectopic pregnancy, delivery)
- Date for delivery / end of pregnancy foetus 3

# STUDY DESIGN

An open label randomized controlled trial.

# STUDY FLOW CHART

# SCHEDULE OF EVENTS TABLE

| Procedures | **Baseline** | **Enrolment** | **1 month prior to surgery or start of first ART cycle^1^** | **Surgery^2^** | **2 months postoperative visit^2^** | **1:st oocyte retrieval** | **2 months after 1:st oocyte retrieval** | **2:nd oocyte retrieval** | **2 months after 2:nd oocyte retrieval** | **3:rd oocyte retrieval** | **2 months after 3:rd oocyte retrieval** | **Visit, phone call or questionnaire 3 years after first treatment (surgery or first ART cykle)** |
| --- | --- | --- | --- | --- | --- | --- | --- | --- | --- | --- | --- | --- |
| **Informed consent oral and written** | x |  |  |  |  |  |  |  |  |  |  |  |
| **Demographics** |  |  | x |  |  |  |  |  |  |  |  |  |
| **Record ultrasound and/or MRI staging according to AAGL and #ENZIAN staging system** | x |  |  |  |  |  |  |  |  |  |  |  |
| **Randomization** |  | x |  |  |  |  |  |  |  |  |  |  |
| **Record surgical procedure according to AAGL and #ENZIAN staging system** |  |  |  | x |  |  |  |  |  |  |  |  |
| **Record intraoperative complications^3^** |  |  |  | x |  |  |  |  |  |  |  |  |
| **Hospital stay** |  |  |  | x |  |  |  |  |  |  |  |  |
| **Record 8 weeks postoperative complications^4^** |  |  |  |  | x |  |  |  |  |  |  |  |
| **EHP-30, EQ5D and NRS scale^5^** |  |  | x |  | x | x | x | x | x | x | x | x |
| **Primary outcome (CLBR)** |  |  |  |  |  |  |  |  |  |  |  | x |
| **Secondary outcome/IVF cycle^6^** |  |  |  |  |  |  | x | x | x | x | x | x |

^1^ start of FSH injections ^2^only first line surgery group ^3^According to Classic ^4^According to Clavien Dindo ^5^Endometriosis Health Profile and Numeric Rating Scale ^6^LBR, PR, number of oocytes retrieved, fertilization grade, number of blastocysts

# TIMELINE


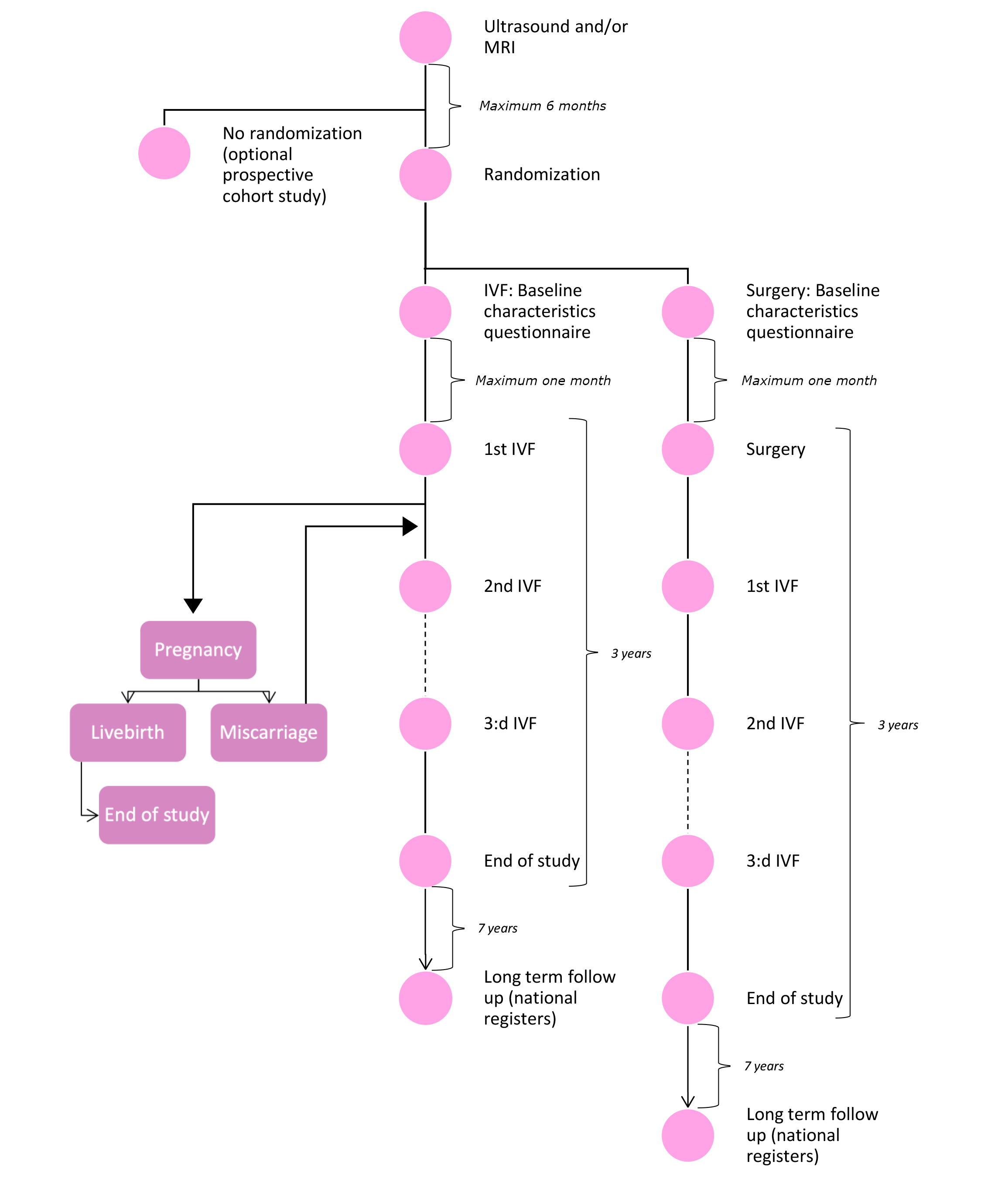


# STUDY ENROLLMENT

SCREENING PROCEDURE AND PARTICIPANT IDENTIFICATION

All women with stage III-IV AAGL endometriosis with infertility referred and/or eligible for surgery and/or ART-treatment such as IVF or ICSI will undergo screening for this trial. The results of this screening will be documented in a screening log. After obtaining oral and written informed consent, patients will be registered and randomized. Registration data will be entered to an electronic Case Report Form (eCRF).

STAGING

At baseline, a specialized transvaginal and abdominal ultrasound examination, supplemented with transabdominal ultrasound if indicated will be performed according to the International Deep Endometriosis Analysis (IDEA) group recommendations (25) to identify endometriosis lesions. Ultrasound and, if needed, magnetic resonance imaging (MRI) findings will be classified using the #ENZIAN and AAGL classification system (4, 6)**.**

The presence of adenomyosis will also be documented and characterized according to the Morphological Uterus Sonographic Assessment (MUSA) group recommendation (26, 27) . Women with and without suspected adenomyosis will receive the same treatment protocols. Presence of adenomyosis will not influence allocation but will be considered during the discussion of the final analysis of study results.

The use of hormonal treatment for endometriosis, prior to the surgery/ first ART-treatment or between the surgery and ART-treatment, will be documented and considered in the discussion of the outcomes.

INCLUSION CRITERIA

- Age 18 - 38 years old
- Endometriosis AAGL stage III-IV
- Referred or eligible for ART-treatment such as IVF or ICSI, independent of infertility diagnosis (28) (including sperm donation cycles for social reasons) and/or infertility due to dyspareunia/dysmenorrhea caused by endometriosis
- Body mass index 18-35 kg/m^2^
- Patients who have signed an approved Informed Consent

EXCLUSION CRITERIA

- Previous surgery for endometriosis except diagnostic laparoscopy.
- Previous IVF/ICSI-cycles (including prior fertility preservation cycles)
- Hemato- and/or hydrosalpinx
- Clear indication for surgery such as ureteral stenosis or intestinal sub-occlusive symptoms
- Suspicion of malignancy
- Submucosal fibroids (The International Federation of Gynecology and Obstetrics (FIGO) 0-1, any size) or intramural fibroids (FIGO 2-5, > 4 cm largest diameter of the largest myoma) (29)
- Uterine malformations (class U1-U6 according to ESHRE/ESGE-classification) (30)
- Patients with contraindications to surgery
- Patients undergoing ART with donated oocytes

RANDOMIZATION

After verification of eligibility, signed informed written consent participants will be randomly assigned to either undergo first-line ART or first-line surgery, followed by ART by equal allocation, 1:1. The randomization procedure will be stratified for participating center (permuted block design). Randomization will be performed at each site using the web-based instrument Red Cap. All inclusion criteria and no exclusion criteria must be met. One month before first treatment (IVF or surgery), inclusion and exclusion criteria are entered into the randomization/registration application RedCap. Patients withdrawn from the study after randomization but before treatment will be substituted by newly enrolled patients. Patients withdrawn from the study after first treatment will not be substituted. Username and password are required to log in; each investigator authorized to register patients has a personal login username and password. If all criteria are met, patients are registered, and the allocated patient number is recorded in the patients’ medical file.

There could be some uncertainty surrounding participants' willingness to engage in a Randomized Controlled Trial (RCT) comparing two fundamentally distinct approaches. Therefore, we intend to offer women who fulfill eligibility criteria but decline randomization the opportunity to take part in a parallel study – a prospective cohort study, that would provide valuable real-world outcomes of two different approaches to treatment of endometriosis -associated infertility. This parallel study will evaluate identical outcomes to those in the randomized trial. However, instead of random assignment, participation decisions will be made by the patients themselves, resulting in what is commonly referred to as a patient preference trial.

DEFINITION START OF TRIAL

Start of trial will be defined as start of first treatment which will be either surgery or first ART cycle (e.g start of FSH injections).

DEFINITION END OF TRIAL

The study will end when all patients enrolled in trial have been followed for 3 years after first treatment, withdrawn consent, or are lost to follow-up. Data from study participants that are lost to follow will be included in the final analysis. The trial steering committee may end enrolment at any time if it is deemed that this is in the best interest of the patients.

# STUDY TREATMENT

Time from randomization to first treatment (surgery or first ART cycle) should be minimized with a goal of three months and should preferably not exceed six months. Complications due to treatments (IVF or surgery) will be register in the eCRF and treated according to clinical routine at each hospital.

EXPERIMENTAL TREATMENT

First line endometriosis surgery followed by ART such as IVF or ICSI.

STANDARD/CONTROL TREATMENT

First line ART such as IVF or ICSI.

SURGICAL PROCEDURE

Surgery will be performed at one of four nationally specialized centers in advanced endometriosis surgery (Södersjukhuset Stockholm, Sahlgrenska University Hospital Göteborg, Skånes University Hospital, Malmö/Lund, Uppsala University Hospital). Intraoperative AAGL endometriosis staging, #ENZIAN classification and Endometriosis Fertility Index (EFI) (31) will be recorded in the study protocol. Surgery will be conducted according to the ESHRE recommendations of surgery in endometriomas and deep endometriosis (32, 33). Surgery should be multidisciplinary when needed (colorectal surgeons and urologists). The goal of the procedure is to remove as many endometriotic lesions as possible while also minimizing negative impact on organ function, since endometriosis is a benign disease. Rectal endometriosis will be removed with rectal shaving, discoid or segmental resection depending on the size and placement of the lesion in the rectal wall. Laparoscopic cyst enucleation is considered the "golden standard" as it is associated with a low recurrence rate, significant pain relief, and increased chances of spontaneous conception (34). In cases where ovarian reserve is already diminished, more gentle methods such as ablation therapy with CO2 Laser/Plasma Jet or sclerotherapy could be employed (35-37). These methods can also be used in combination with conventional surgical cyst enucleation. The choice of surgical technique should be tailored to the individual patient's needs.

In Sweden in 2023, 9.9% of patients undergoing surgery for endometriosis within the framework of national highly specialized care experienced mild complications Clavien Dindo 1-2 (e.g. infection and hematoma at the surgical site), and 3.3% experienced serious complications Clavien Dindo 3 (e.g. bowel leakage, unexpected bladder injury, ureteral injury, or intra-abdominal bleeding requiring reoperation) (38). Serious complications may, in rare cases, necessitate further surgical interventions. The risk of complications varies slightly depending on the location of the endometriosis (38).

Patients undergoing surgery will need to delay ART treatment by two to three months in order to fully recover.

FERTILITY TREATMENT WITH ART

ART treatments will be carried out at Swedish reproductive units authorized to provide publicly funded fertility care. The majority of treatments are expected to take place at the university-affiliated reproductive clinics in Stockholm, Gothenburg, Skåne, and Uppsala. However, for participants residing in other regions of Sweden, undergoing treatment locally offers a significant advantage by reducing the logistical and emotional burden of long-distance travel to these central clinics. To ensure equitable access and minimize patient stress, regional ART centers will be considered when appropriate.

One IVF/ICSI-cycle is defined as the transfer of all the embryos created via that particular oocyte pick-up until pregnancy confirmation or until the failure of the last embryo transfer. In case of a cancelled cycle prior to oocyte pick-up it will not be counted. The duration of one IVF-cycle depends on the number of embryo transfers and typically ranges between two and nine months.

ART treatment: Women allocated in the ART arm will undergo up to three cycles of controlled ovarian stimulation (COS) followed by oocyte retrievals and embryo transfers. In Sweden, ART-treatment costs for women under the age of forty and without having children in current relationship are covered by the tax-funded healthcare system, with up to 3 IVF/ICSI treatments, given that they are considered medically meaningful, and that each stimulation is started before the woman turns 40 years of age (39). In case the treatment results in cryopreserved embryos, it is planned that they should be transferred prior to the start of any new COS. Women will be managed according to a routine clinical protocol at each reproductive unit. Stimulation protocols will be chosen individually by clinicians, taking into consideration age and ovarian reserve of each woman, as it is a standard routine in the collaborating Reproductive Units. No aspiration of endometrioma prior to oocyte pick-up will be performed. Antibiotic prophylaxis at the day of oocyte pick-up will be given according to local guidelines.

# QUALITY ASSURANCE OF SURGERY AND FERTILITY TREATMENT

PARTICIPATING CENTERS

All participating surgical centers have been selected by the National Board of Health and Welfare following an application process to perform highly specialized advanced endometriosis surgery at a national level. A quality assessment form including institutional experience with endometriosis surgery and ART, annual volume of benign gynecological complex cases must be completed. Moreover, surgical variables (e.g. operation time, blood loss) and complications within 30 days after surgery according to Clavien Dindo (22) must be reported . Furthermore, the infrastructure to participate in the trial must be satisfactory. In addition, the institution’s ability to perform staging of endometriosis is considered. All ART-clinics authorized to provide publicly funded fertility care report their results continuously to the National Quality Registry for Assisted Reproduction (Q-IVF).

During the study, it is at the discretion of the coordinating investigators and Trial steering committee to close centers with a higher-than-average rate of postoperative major

complications or poor quality of surgery or ART, from further accrual, temporarily or irrevocably after consultation with the Data Safety Monitoring Board.

PARTICIPATING SURGEONS

All included surgeons must be approved by the coordination investigators or/and trial steering committee ensuring adherence to protocol. In the site identification and quality assessment form the participating surgeons experience and annual caseload will be reported for review. It is at the discretion of the coordinating investigators or/and steering committee to select or deselect individual surgeons from participating in the trial. Only surgeons stated in the Quality assessment form are allowed being lead surgeons, amendments during the trial can be made.

Robot-assisted laparoscopic or traditional laparoscopic endometriosis surgery

All included surgeons must have a previous experience of at least 20 advanced endometriosis surgeries.

# PATIENT REPORTED OUTCOMES (PROMS)

When evaluating quality of life among women with endometriosis many different scales have been used, mostly of generic character that are not specific to the disease. One of these is 30-item Endometriosis Health Profile (EHP30), a scale that perform well in clinical practice in the routine evaluation of Health-Related Quality of life (HRQoL) The use of EHP30 is recommended by the National Board and Welfare in Sweden, the ASRM and ESHRE (40). Using EHP-30, a small study has shown an improvement in HRQoL in the follow-up after endometriosis surgery, an effect that may last for up to 6.8 years (41). Two recent studies show postoperative improvement in HQQoL measured by EHP-30 10 weeks and 3 months after surgery, respectively (42, 43). Especially women with deep endometriosis (DE) showed a more significant benefit from surgery (43). However, HRQoL can also be affected by infertility, and in this trial, infertility treatment will be delayed, thus, HRQoL is an important measurement.

POINTS OF ASSESSMENT

Baseline assessment will be obtained after randomization and maximum 1 month prior to surgery or start of first ART cycle

Thereafter assessment will be done prospectively at:

- 2 months after surgery (for the group randomized to first line surgery)
- 1:st oocyte pick up (for both randomization groups)
- 2 months after 1:st oocyte pick up
- 2:nd oocyte pick up
- 2 months after 2:nd oocyte pick up
- 3:rd oocyte pick up
- 2 months after 3:rd oocyte pick up
- Visit, phone call or questionnaire 3 years after randomization.

Compliance with completing the questionnaires will be investigated at each time point to

evaluate the procedure for data collection and the feasibility of the questionnaires.

INSTRUMENTS

The core questionnaire in EHP -30 includes 30 questions in 5 health-related areas: pain, control and powerlessness, emotional well-being, social support, and self-image. In addition, there are six supplementary modules covering work, relationship with child/children, sexual relationship, feelings about the medical profession, feelings about treatment and feelings about infertility.

The Numeric Rating Scale (NRS) is a frequently utilized pain assessment tool commonly employed in postoperative care. It allows for the momentary evaluation of pain on a scale from 0 to 10, with 0 representing "no pain" and 10 indicating "the worst pain imaginable”.

EQ-5D is a standardized non-disease specific instrument for describing and

valuing HRQoL, developed by the EuroQoL group. It includes five dimensions (mobility,

self-care, usual activities, pain/discomfort and anxiety/depression) with three levels of

responses each (no problems, some problems or extreme problems). The EQ-5D also

comprises a 20cm visual analogue scale (VAS) ranging from 0 (worst imaginable

state) to 100 (best imaginable health state) on which the respondent rates the current health.

The index-based score is interpreted along a continuum where 1 represents best possible health and 0 represents dead. Some health states are given a figure below zero (worse than

death).

PROCEDURE

The first questionnaire kit (EHP-30, NRS and EQ-5D) will be administered after the patient is informed about their randomized study arm. This timing has been chosen to ensure that the baseline assessment is collected a similar length of time before the planned intervention in both study arms, since the date of surgery or ART treatment can only be scheduled after randomization. This approach minimizes bias related to varying waiting times between randomization and treatment initiation., The patient will receive both oral and written information about the Health-Related Quality of Life (HRQoL) assessment. Subsequent questionnaires will be collected during clinic visits, electronically, or via conventional mail at predefined time-points (please see above). Treating physicians will not have access to the HRQoL study forms. Completed questionnaires will always be considered source documents and must be filed accordingly.

# HEALTH ECONOMICS

Health economics will be analyzed with respect to Swedish conditions first and with an international perspective.

DIRECT COSTS

To evaluate healthcare costs, the internal accounting and billing systems within hospitals will be utilized to estimate direct costs based on Cost Per Patient (CPP) principles for individual treatments and/or Diagnosis-Related Groups (DRG) for surgery and fertility treatment. We will also obtain CPP and/or DRG-based cost data from the hospitals where the included women receive care in connection with surgery, fertility treatments, pregnancy, childbirth, and postpartum follow-up. In addition, data on employment status at baseline will be collected. Quality-Adjusted Life Years (QALY) will be computed using data from the EQ-5D instrument. Variations in changes to health status and quality of life will be utilized for a cost-utility analysis, with the results presented as an Incremental Cost-Effectiveness Ratio (ICER). This analysis will provide insights into the additional cost per year of full health achieved with first line surgery compared to first line ART.

INDIRECT COSTS

To assess indirect costs related to the disease, both study arms will be examined with regard to estimating productivity costs, specifically the level of decline in production. Methods such as the friction cost method may be employed for this purpose.

# ADENOMYOSIS

The presence of suspected adenomyosis, on ultrasound and/or MRI, will be recorded and described at baseline according to Morphological Uterus Sonographic Assessment (MUSA) (26, 27). The presence of adenomyosis will not affect allocation but will be used at the end of the study in the interpretation of the findings, as a sub-group analysis.

# OBSTETRICAL OUTCOMES

Obstetrical outcomes and complications will be collected from patient journals and from the National Pregnancy register: Obstetrical outcomes (gestational age at the delivery, birth weight, umbilical artery and vein blood analysis, neonatal complications within 7 days after delivery, and complications (pregnancy-related complications (premature delivery, preeclampsia, gestational diabetes, placenta previa, placenta abruptio, placenta spectrum disorders, bleeding complications during pregnancy); delivery-related complications (instrumental delivery, emergency caesarean section, pelvic floor trauma, delivery related bleeding, manual placenta removal); complications within 8 weeks postpartum (infection (endometritis, pelvic inflammatory disease, urinary tract infection, wound infection, sepsis), remaining conceptual tissue with and without surgical intervention, use of analgesics). In case of missing information women will be contacted by phone for additional information.

The power calculation was conducted for the primary outcome, hence, there may be insufficient power to compare pregnancy complications due to the small proportion of complications and the fact that all participants will not experience a complete pregnancy. To better assess pregnancy complications, we will compare the study population with the broader population of pregnant women. In addition to the comparison between the two randomized groups the whole study population will be compared with women giving birth during the time the study is running with a diagnosis of endometriosis and no surgical treatment. To identify the control group, data from the Patient Register (including outpatient and inpatient diagnoses) and the Swedish National Quality Register of Gynecological Surgery (Gynop) will be linked with the National Pregnancy Register (2025 to the study's end). Pregnancies complicated by endometriosis stage III-IV and surgery treatment will be compared with those with endometriosis and no surgical treatment as well as a comparison with women without endometriosis. Although undiagnosed endometriosis in the pregnant population poses a challenge in creating a control group free of endometriosis or with a specific stage of endometriosis, linking the gynaecological operation and register with diagnosis will allow the creation of control groups with largely correct status of endometriosis. Each control group is planned to include 1000 persons.

# PROSPECTIVE COHORT STUDY

For patients who decline participation in randomization or are excluded after randomization, an option to be included in an observational prospective cohort study will be provided. Participants in this study will adhere to the same study protocol as in the RCT, except for the randomization itself. Analyses for this cohort will adjust for confounding factors using regression models, as described in the statistical methods section.

# LONG-TERM FOLLOW-UP

There will be an opportunity for inclusion in a long-term follow-up, where participants from both the randomized controlled study and the cohort study will be eligible if they consent. In the long-term follow-up, conducted 10 years post-inclusion, we will utilize the unique Swedish personal identification number and gather data after study conclusion on infertility treatments from the National Register of ART in Sweden (Q-IVF) live births from the Swedish Medical Birth Register (MFR) and The National Pregnancy Register and number of recurrent endometriosis surgeries from Gynop. For details about variables collected from each register see appendix.

# THE REPRODUCTIVE OUTCOMES RELATED TO P-PROGESTERONE ON THE DAY OF TRANSFER OF THE CRYOPRESERVED EMBRYO WITH HORMONE REPLACEMENT THERAPY-CYCLES

Patients suffering of endometriosis may differ in their response to exogenous progesterone when undergoing frozen embryo transfer cycles(FET) with standard hormone replacement therapy, HRT (44). The aim of this observational sub-study is to investigate possible association between P- progesterone levels on the day of FET in patients with endometriosis undergoing HRT cycles and their reproductive outcomes. Primary outcome will be live birth rate and secondary outcomes will include clinical pregnancy rate and miscarriage rate. Potential difference in P-progesterone levels between the two different study groups (surgery first vs IVF first) will also be investigated.

Blood samples to analyze P-progesterone will be collected on the day of FET, and the test result will be recorded in the eCRF, as a sub-group analysis. The subgroup analysis includes only HRT-FET cycles in patients with endometriosis AAGL stage III-IV diagnosed by either ultrasound or surgery. The standard HRT protocol for endometrial preparation is usually performed by using Progynon (4-8 mg/d) per os with the start on the first, second or third day of menstruation with a possible addition of transdermal estrogen according to local routine. Cyclogest (400 mg/12 h) or other vaginal progesterone preparation of similar efficacy should be commenced when the endometrium is regarded as appropriate by the treating physician (as a rule 7 mm or thicker). Blastocyst transfer should be scheduled on the sixth day of progesterone supplementation (45).

# STATISTICAL CONSIDERATIONS

The primary exposure is the randomized allocation (surgery prior to ART vs ART alone). All analyses will follow the intention-to-treat (ITT) principle, meaning participants are analyzed according to their randomized group regardless of adherence. Per-protocol and as-treated analyses will be conducted as sensitivity analyses to assess robustness. An interim analysis at the halfway point is planned to monitor potential adverse effects and ensure the safety of the interventions.

No formal adjustment for multiplicity will be made; the primary endpoint will be tested at a two-sided α=0.05, while secondary endpoints are considered supportive and exploratory. Sensitivity analyses will address protocol deviations, handling of missing data, and robustness to alternative modelling approaches.

DATA ANALYSES

Baseline demographic and patient’s characteristics will be summarized by treatment group. Continuous variables will be described with mean, standard deviation, median, interquartile range, minimum and maximum, and categorical variables with counts and percentages.

Primary endpoint

The primary endpoint of the study is the Cumulative Live Birth Rate (CLBR), defined as the proportion of participants achieving at least one live birth within three years following the initiation of treatment.

**Primary analysis**

The primary analysis will compare proportions between treatment groups and report the crude relative risk (RR) with 95% confidence intervals. Adjusted analyses will use log-binomial regression (or modified Poisson with robust variance if convergence fails), including prespecified covariates (age, BMI, AMH, ART type, center). A two-sided α=0.05 will be used.

**Exploratory subgroup analyses**

Exploratory subgroup analyses will be conducted using treatment-by-subgroup interaction terms in regression models. Subgroups of interest include type of ART (IVF vs ICSI), age (<35 vs ≥35 years), ovarian reserve (AMH categories), and surgical characteristics (adnexal surgery yes/no; anterior vs posterior deep endometriosis, KVÅ codes). These analyses are exploratory and not adjusted for multiplicity.

Secondary endpoints

**Time-to-Event outcomes**

Cumulative Live Birth Rate (CLBR) and Cumulative Pregnancy Rate (CPR) will be analyzed as time-to-event outcomes, defined as the proportion of participants achieving a live birth or pregnancy within three years following initiation of treatment. Kaplan–Meier curves will be used to display cumulative incidence, and groups will be compared with stratified log-rank tests. Treatment effects will be estimated using Cox proportional hazards models, stratified by center and adjusted for prespecified covariates. Hazard ratios with 95% confidence intervals will be reported.

**Cycle-Specific Outcomes**

Live birth and pregnancy rates per IVF/ICSI cycle will be analyzed using log-binomial regression or modified Poisson regression with robust variance to account for within-patient correlation. Other cycle-specific outcomes (e.g. number of oocytes retrieved, fertilization rate, embryo transfer characteristics, miscarriage rates) will be summarized descriptively and compared between groups using appropriate regression models.

**Quality of Life and Health Status Measures:**

**EHP-30 (continuous, 0–100 scale):**
Analyzed using a linear mixed-effects model (LMM) with random intercepts for participants. Fixed effects will include treatment group, time, and the treatment×time interaction, adjusting for prespecified covariates (age, BMI, AMH, center).

**EQ-5D (continuous index 0–1):**Analyzed using the same LMM framework as EHP-30 (random intercepts, treatment, time, treatment×time, prespecified covariates).

**NRS pain (ordinal: mild 1–3, moderate 4–6, severe 7–10):**
Analyzed using a generalized linear mixed model (GLMM) with cumulative logit link (ordinal logistic regression), including random intercepts for participants, fixed effects for treatment, time, and treatment×time, and prespecified covariates (age, BMI, AMH, center).

**Health economics**
A cost-effectiveness analysis will be conducted from the healthcare perspective. Incremental cost-effectiveness ratios (ICER) will be calculated based on QALYs, and probabilistic sensitivity analyses will be performed to address parameter uncertainty.

**Additional analysis**

– Exploratory subgroup analyses will not be adjusted for multiplicity.

– Safety outcomes will be summarized descriptively by treatment group (frequency, severity, relation to treatment).
– All available data will be used, including outliers.
– Missing outcome data will be handled under the missing-at-random assumption via maximum likelihood in mixed models; for regression models requiring complete covariates, complete-case analysis will be applied.

Covariates

Primary and secondary outcomes will be adjusted for prespecified covariates (age, BMI, AMH, ART type, center), chosen based on clinical relevance and prior evidence.

POWER CALCULATION AND SAMPLE SIZE

The sample size calculation was based on a two-sample comparison of proportions with a two-sided α=0.05 and 80% power. Based on observational data (18, 19), the cumulative live birth rate (CLBR) after three IVF cycles was assumed to be 55% in the ART-only group and 71% in the surgery+ART group (relative risk ≈1.29). This yields a required sample size of 142 participants per arm (284 total). Allowing for 20% attrition, the planned total sample size is 350 participants.

The necessary sample size for this study is estimated to 323 patients to accommodate a statistical analysis method. This estimation uses the 'pwr' package in R, specifically employing the power.prop.test method, which is designed to compare two proportions. This method is more aligned with contemporary statistical practices and provides a clear, transparent methodology for determining the required sample size based on expected success rates.

This means inclusion of 29 patients from/each center/year and 15 surgeries/year. In a Swedish study published in 2022 the prevalence of endometriosis in patients referred for ART-treatment was 21.8% and 17.2% of women had DE (14). In 2021 almost 10 500 IVF cycles were performed in Sweden, while more than 5 500 of those were first-time IVF/ICSI with autologous gametes ((46) Figure 22).

# INTERIM ANALYSIS AND STOPPING RULES

An independent safety and monitoring board (DSMB) will conduct one interim analysis when 175 patients have been enrolled in the study and completed either their surgery or first IVF treatment, whichever occurs first. Because each participant has a three-year follow-up and recruitment will be completed within three years, only a limited proportion of primary outcomes will be available at the interim analysis. The analysis will therefore primarily address recruitment feasibility, participant safety including comparison of complication rates between study centers.  The DSMB will monitor adverse events in both arms. For surgery, expected complication rates have been described previously in this protocol (9.9% Clavien–Dindo grade 1–2, 3.3% grade ≥3), while ART carries a different spectrum of risks (e.g. ovarian hyperstimulation, infection, or bleeding). A substantially higher-than-expected complication rate in either arm, major discrepancies between centers or other safety concerns may prompt the DSMB to recommend protocol modifications or early termination.

Recommendations from the DSMB will exclusively be communicated to the Trial Steering Committee. Further details regarding the DSMB can be found in the DSMB charter (Appendix).

# ETHICAL CONSIDERATIONS

RISK-BENEFIT CONSIDERATION

By addressing the research questions, we aim to provide evidence to improve patient outcomes in a population that currently faces challenges in fertility treatment due to endometriosis. The research has the potential to benefit participants by improving their chances of successful conception and live birth rates.

Participants in the study may face several direct risks, including physical risks associated with surgery and assisted reproductive technologies, such as infection, or pre- and postoperative complications. Additionally, there may be psychological risks related to the emotional stress of undergoing medical procedures and the uncertainty of treatment outcomes. Furthermore, there are also risks associated with the collection and storage of sensitive medical data, which must be mitigated through robust data protection measures.

In the long term, the research may contribute to improving the understanding and management of endometriosis-related infertility, thereby potentially benefiting future patients.

INSTITUTIONAL REVIEW BOARD/ETHICS COMMITTEE

The study protocol, patient information, and informed consent are approved by Swedish Ethical Review Authority ( dnr: 2024-04293-01, approved 7^th^ October 2024, amendment, dnr: 2025-03699-02, approved 17^th^ June 2025).

Any significant protocol modifications must be submitted to the relevant Independent Ethics Committee or Institutional Review Board for review and approval before implementation. Upon approval from the appropriate committee or board, the investigator will proceed with implementing such protocol modifications. However, in cases of urgent safety concerns, protocol modifications will be promptly implemented without prior approval.

INFORMED CONSENT AND WITHDRAWAL

Before being enrolled in the study, patients will receive both oral and written information on the study objectives, all treatment procedures, and the anticipated and potential adverse events. They will be informed about the strict confidentiality measures regarding their patient data, ensuring that only their treating physician and authorized study personnel will have access to their medical records. Patients will have the freedom to withdraw their consent for study participation at any time, with or without providing a reason, and this decision will not impact their subsequent treatment options or care.

Written informed consent must be obtained from all participants before they are enrolled in the study. The investigator who provided the written and verbal information should also sign the Informed Consent Form during the same encounter. The signed Informed Consent Form should be retained in the Investigator's File, and a copy should be provided to the study participant.

Participants will consent to the following:

- Participation in the study
- Regulatory authorities and the sponsor's representatives (e.g., monitor) gaining full access to hospital records.
- The control and collection of data for the study
- The recording, collection, processing, and storage of data in a database
- Using of data and images for education, lectures, scientific presentations and publications

PATIENT PROTECTION AND GOOD CLINICAL PRACTICE

The responsible investigator will ensure that the study is conducted in accordance with the principles outlined in the Declaration of Helsinki and/or relevant Swedish or National laws and regulations, whichever offers the highest level of protection for the patient. Participants will be clearly informed that the data collected in the study will adhere to the General Data Protection Regulation (GDPR) (EU 2016/679), ensuring that no subject participating in the study will be identifiable. Women participating in the study will receive treatment in alignment with the international guidelines on Good Clinical Practice (GCP) as defined by the European Parliament (EG596/200).

SUBJECT IDENTIFICATION

Participating patients will be assigned a study-specific code, comprising a two to six-digit number. This code will be utilized for patient registration in the study database. The woman's social identification number will not be included in the database. The key to decipher the code will be accessible solely to the investigator.

# GENDER PERSPECTIVE

Endometriosis is a prevalent condition, yet it is rarely acknowledged as a public health concern, despite its pervasive impact across patients' lifecycles. National guidelines were not issued until 2018, and many women with endometriosis report a lack of awareness and understanding of the disease within society (47). Diagnostic delays are documented in some studies, indicating that patients can suffer from symptoms of this disease up to ten years before a diagnosis of endometriosis is made and treatment is started (48).

A Swedish epidemiological study conducted in 2019 revealed that 45% of teenage girls experience frequent school absences due to dysmenorrhea, yet only 7% of these girls were given the advice to seek doctor consultation (49). Subfertility often arises as a consequence of endometriosis. In another Swedish study involving women seeking assistance for fertility issues, 75.8% of patients were unaware of their endometriosis diagnosis upon initial consultation at the reproductive medicine center (3). This suggests that society commonly perceives menstrual pain as a normal discomfort that women are expected to endure.

# SIGNIFICANCE OF STUDY

Clinical significance

Endometriosis is a prevalent disease among women with infertility, with negative impact on psychological and physical well-being. The management of infertility in women with severe endometriosis is a subject of ongoing debate, with a lack of robust evidence for optimal treatment strategies. Both surgery and ART-treatments in this population may lead to serious complications and are associated with significant costs to society. To date, there are no results from RCTs evaluating potential benefits of endometriosis surgery prior to ART on subsequent reproductive outcomes. This study has the potential to significantly impact the clinical approach to infertility treatment in women with advance endometriosis. Despite the challenges and the coordinated effort required for this RCT, the results promise to be valuable for the scientific and clinical communities.

Implementation

Endometriosis is a very prevalent condition (22 % of infertile women) and this research project will indeed affect many patients, regardless of the results.

The research group is presently drafting national guidelines for the treatment of infertility in patients with advanced endometriosis. The findings of this study will be integrated into these guidelines to assist decision-making for women with severe endometriosis and infertility.

Additionally, in a consensus document from the European Society of Human Reproduction and Embryology (ESHRE) on endometriosis, it is noted that there is a dearth of randomized controlled trials regarding surgery prior to assisted reproductive technology.

The results of the study will most probably affect guidelines and care for women with severe endometriosis and infertility. It will possibly also lead to insights how to treat women with less severe endometriosis. Other aspects, such as centralization of advanced surgery as a concept will also be tested.

# ADMINISTRATIVE CONSIDERATIONS

FINANCING

This academic study is sponsored by Region Stockholm and Karolinska Institutet, Forte, Västra Götaland Region and the Swedish Medical Society.

The Obstetric and Gynecological department at Södersjukhuset, Stockholm, will serve as the coordinating entity for the study. All central administrative expenses associated with the trial, including statistics, monitoring, and questionnaires, are funded through research grants. The aim is to secure adequate grants to cover, either partially or fully, the study-specific costs for each participating site. Each participating center is at liberty to seek financing independently.

PUBLICATION POLICY

Prior to publication of the main fertility outcome, no other publications concerning fertility outcomes on segments of the cohort may be pursued. The Coordinating Investigator and principal investigator Stockholm (Malin Brunes and Anna Marklund) will serve as first and last authors of the primary outcome and fertility outcome publication. Additionally, members of the Trial Steering Committee must be listed as (co)-authors in all other publications. Each member of the sub-committees will be listed as authors for their respective sub-objectives. The chair of each sub-committee will be either the first or last author and will compile the initial manuscript draft.

The Coordinating Investigator and principal investigator Stockholm will contribute to the data interpretation and determine the authorship order for each publication from the sub-committees. One author (principal investigator) from each participating site, contingent upon data completeness and quality, will be listed as a co-author on any publication reporting the main findings of the Endo-SOFT trial, including reports on fertility outcomes and other significant publications. In cases where the number of authors is restricted by the scientific journal, contributing sites with the highest participant recruitment and completed follow-up will be selected.

All investigators must agree that, upon completion of data collection and analysis by the study statistician, if they do not concur with the outcome results, they reserve the right to abstain from authorship of the manuscript. However, the data collected from their site will be retained and analyzed as agreed upon at the study's inception, consistent with this agreement.

Sub-analysis other than primary and secondary outcomes from the Endo-SOFT trial

Additional sub-analyses or research projects beyond the primary and secondary outcomes outlined in the Endo-SOFT trial are permitted and encouraged. However, permission must be sought from the Trial Steering Committee through a written application. This application must include details such as the primary and secondary objectives, inclusion and exclusion criteria, the name of the individual responsible for manuscript preparation (including the first and last author), the intended journal for submission, and approval from the respective institutional review board. The final decision regarding authorship will be made by the Trial Steering Committee.

ADHERENCE TO PROTOCOL AND PROTOCOL AMENDMENT

Adherence to the study protocol is imperative, and any deviations must be meticulously documented. The Trial Steering Committee must be promptly notified of any such deviations. Changes or additions to the study protocol can only be deliberated upon and authorized by the coordinating investigator, study coordinator, trial steering committee, and statistician. Following approval by the appropriate Independent Ethics Committee or Institutional Review Board, the investigator is responsible for implementing these protocol modifications. However, in cases of urgent safety concerns, protocol modifications shall be implemented immediately.

# DATA MANAGEMENT AND QUALITY CONTROL

DATA COLLECTION

Data for the randomized controlled study will be collected from medical records. In case of missing information patients will be contacted by phone or through secure electronic data platforms (e.g “Alltid Öppet”) for additional information. Data from ultrasound examination, surgeries and IVF treatments will be documented either directly into the eCRF or in paper forms. The results from the blood samples (AMH and P-Progesteron) will be collected from the medical records. No blood samples will be stored for future use in this study.

For the sub-studies data will be collected from the following sources:

- Quality of Life: Questionnaires are sent from the eCRF RedCap and patients´ answers will be electronically collected directly into the eCRF.
- Health Economy: Cost per patient will be collected from each site’s economical system.
- Adenomyosis: Medical records and Data forms or paper forms as described above.
- P-Progesterone: Medical records and Data forms or paper forms as described above.
- Obstetrical Outcomes: Either from medical records or the MFR at the end of the study. If data is missing, patients can be contacted by phone or through secure electronic data platforms (e.g “Alltid Öppet”) for additional information.
- Prospective cohort study: Data will be collected in the same way as in the RCT.
- Long-term follow up: Data will be collected from Q-IVF, MFR, Gynop and the Swedish National Pregnancy Register. We will use the unique Swedish identification number to identify the patients from the registers.
- P-Progesterone and reproductive outcome: Data will be collected in the same way as in the RCT.

SOURCE DATA AND CASE REPORT FORM

Patient medical records will serve as the source data and will be stored in accordance with Good Clinical Practice (GCP) guidelines. Data for this study will be captured using the Research Electronic Data Capture (RedCap) system, utilizing an electronic Case Report Form (eCRF). The information will be transcribed from paper source documents onto the eCRF by the site. To ensure accurate and reliable data collection, the study monitor will conduct verification and cross-check of the eCRFs against the investigator’s records (source document verification). The study database is located in Sweden at Clinical Trials Office (CTO), Center for Clinical Cancer Studies, Karolinska University Hospital.

DATA RECORDING AND RECORD KEEPING

Data recording and data management will be overseen by Clinical Trials Office (CTO), Center for Clinical Cancer Studies, Karolinska University Hospital. The data will be securely stored for a minimum of 10 years after the trial's conclusion. Access to the data will be granted exclusively through the Principal Investigator or designated Investigators.

DATA PROTECTION

Recorded information is treated with confidentiality, and the database is privacy-protected, ensuring that no data can be linked back to individual patients in research reports, and unauthorized access to individual data is prohibited. The database will be retained until further notice, with a minimum duration of at least 10 years after the inclusion of the last patient, and all reporting will be conducted in compliance with the General Data Protection Regulation (GDPR). The authority responsible for overseeing the database in RedCap is Region Stockholm, Clinical Trials Office (CTO), Center for Clinical Cancer Studies, Karolinska University Hospital.

PARTICIPANT CONFIDENTIALITY

The investigator is responsible for ensuring the maintenance of patients' anonymity and protection of their identities from unauthorized access. When completing Case Report Forms (CRFs) or any other documents submitted to the Trial Steering Committee, patients should be identified solely by an identification code rather than by their names. Additionally, the investigator must maintain a patient enrollment log that records codes, names, and addresses.

STORAGE OF STUDY DOCUMENTS

In accordance with national and international guidelines, the patient identification list, patient records, and other study-related documents will be preserved for a minimum of 10 years following the conclusion of the trial. Access to this data will be restricted solely to the investigator(s) and personnel appointed by the investigator who are directly involved in the clinical trial.

QUALITY CONTROL AND MONITORING

The quality control for this trial in Sweden will be overseen by Clinical Trial Office (CTO), Karolinska Comprehensive Cancer Center. Regular monitoring of this trial will adhere to Good Clinical Practice (GCP) guidelines and local regulations. All data recorded in the electronic Case Report Forms (eCRFs) will be duly documented in the patient's file, unless stated otherwise. The investigator will allocate sufficient time for visits conducted by the monitor. Additionally, the investigator will ensure that the monitor has access to source documents corroborating the data entered into the eCRFs. Furthermore, the investigator guarantees direct access to source data for potential regulatory audits mandated by the trial.

# REFERENCES

1. Giudice LC, Kao LC. Endometriosis. Lancet (London, England). 2004;364(9447):1789-99.

2. Hamdan M, Omar SZ, Dunselman G, Cheong Y. Influence of endometriosis on assisted reproductive technology outcomes: a systematic review and meta-analysis. Obstetrics and gynecology. 2015;125(1):79-88.

3. Alson S, Jokubkiene L, Henic E, Sladkevicius P. Prevalence of endometrioma and deep infiltrating endometriosis at transvaginal ultrasound examination of subfertile women undergoing assisted reproductive treatment. Fertility and sterility. 2022;118(5):915-23.

4. Abrao MS, Andres MP, Miller CE, Gingold JA, Rius M, Neto JS, et al. AAGL 2021 Endometriosis Classification: An Anatomy-based Surgical Complexity Score. Journal of minimally invasive gynecology. 2021;28(11):1941-50.e1.

5. Abrao MS, Andres MP, Gingold JA, Rius M, Neto JS, Goncalves MO, et al. Preoperative Ultrasound Scoring of Endometriosis by AAGL 2021 Endometriosis Classification Is Concordant with Laparoscopic Surgical Findings and Distinguishes Early from Advanced Stages. Journal of minimally invasive gynecology. 2023;30(5):363-73.

6. Keckstein J, Saridogan E, Ulrich UA, Sillem M, Oppelt P, Schweppe KW, et al. The #Enzian classification: A comprehensive non-invasive and surgical description system for endometriosis. Acta obstetricia et gynecologica Scandinavica. 2021;100(7):1165-75.

7. Becker CM, Bokor A, Heikinheimo O, Horne A, Jansen F, Kiesel L, et al. ESHRE guideline: endometriosis. Hum Reprod Open. 2022;2022(2):hoac009.

8. Opøien HK, Fedorcsak P, Byholm T, Tanbo T. Complete surgical removal of minimal and mild endometriosis improves outcome of subsequent IVF/ICSI treatment. Reproductive biomedicine online. 2011;23(3):389-95.

9. Bafort C, Beebeejaun Y, Tomassetti C, Bosteels J, Duffy JM. Laparoscopic surgery for endometriosis. The Cochrane database of systematic reviews. 2020;10(10):Cd011031.

10. Dabi Y, Ebanga L, Favier A, Kolanska K, Puchar A, Jayot A, et al. Discoid excision for colorectal endometriosis associated infertility: A balance between fertility outcomes and complication rates. Journal of gynecology obstetrics and human reproduction. 2024;53(2):102723.

11. Bendifallah S, Puchar A, Vesale E, Moawad G, Daraï E, Roman H. Surgical Outcomes after Colorectal Surgery for Endometriosis: A Systematic Review and Meta-analysis. Journal of minimally invasive gynecology. 2021;28(3):453-66.

12. Horton J, Sterrenburg M, Lane S, Maheshwari A, Li TC, Cheong Y. Reproductive, obstetric, and perinatal outcomes of women with adenomyosis and endometriosis: a systematic review and meta-analysis. Human reproduction update. 2019;25(5):592-632.

13. Barnhart K, Dunsmoor-Su R, Coutifaris C. Effect of endometriosis on in vitro fertilization. Fertility and sterility. 2002;77(6):1148-55.

14. Muteshi CM, Ohuma EO, Child T, Becker CM. The effect of endometriosis on live birth rate and other reproductive outcomes in ART cycles: a cohort study. Hum Reprod Open. 2018;2018(4):hoy016.

15. Somigliana E, Benaglia L, Paffoni A, Busnelli A, Vigano P, Vercellini P. Risks of conservative management in women with ovarian endometriomas undergoing IVF. Human reproduction update. 2015;21(4):486-99.

16. Senapati S, Sammel MD, Morse C, Barnhart KT. Impact of endometriosis on in vitro fertilization outcomes: an evaluation of the Society for Assisted Reproductive Technologies Database. Fertility and sterility. 2016;106(1):164-71.e1.

17. Casals G, Carrera M, Domínguez JA, Abrão MS, Carmona F. Impact of Surgery for Deep Infiltrative Endometriosis before In Vitro Fertilization: A Systematic Review and Meta-analysis. Journal of minimally invasive gynecology. 2021;28(7):1303-12.e5.

18. Ferrier C, Hini JD, Gaillard T, Grynberg M, Kolanska K, Dabi Y, et al. First-line surgery vs first-line ART to manage infertility in women with deep endometriosis without bowel involvement: A multi-centric propensity-score matching comparison. European journal of obstetrics, gynecology, and reproductive biology. 2023;280:184-90.

19. Bendifallah S, Roman H, Mathieu d'Argent E, Touleimat S, Cohen J, Darai E, et al. Colorectal endometriosis-associated infertility: should surgery precede ART? Fertility and sterility. 2017;108(3):525-31.e4.

20. Cimadomo D, de Los Santos MJ, Griesinger G, Lainas G, Le Clef N, McLernon DJ, et al. ESHRE good practice recommendations on recurrent implantation failure. Hum Reprod Open. 2023;2023(3):hoad023.

21. Rosenthal R, Hoffmann H, Clavien PA, Bucher HC, Dell-Kuster S. Definition and Classification of Intraoperative Complications (CLASSIC): Delphi Study and Pilot Evaluation. World J Surg. 2015;39(7):1663-71.

22. Clavien PA, Barkun J, de Oliveira ML, Vauthey JN, Dindo D, Schulick RD, et al. The Clavien-Dindo classification of surgical complications: five-year experience. Annals of surgery. 2009;250(2):187-96.

23. Gardner DK, Lane M, Stevens J, Schlenker T, Schoolcraft WB. Blastocyst score affects implantation and pregnancy outcome: towards a single blastocyst transfer. Fertility and sterility. 2000;73(6):1155-8.

24. Ziebe S, Petersen K, Lindenberg S, Andersen AG, Gabrielsen A, Andersen AN. Embryo morphology or cleavage stage: how to select the best embryos for transfer after in-vitro fertilization. Human reproduction (Oxford, England). 1997;12(7):1545-9.

25. Guerriero S, Condous G, van den Bosch T, Valentin L, Leone FP, Van Schoubroeck D, et al. Systematic approach to sonographic evaluation of the pelvis in women with suspected endometriosis, including terms, definitions and measurements: a consensus opinion from the International Deep Endometriosis Analysis (IDEA) group. Ultrasound in obstetrics & gynecology : the official journal of the International Society of Ultrasound in Obstetrics and Gynecology. 2016;48(3):318-32.

26. Harmsen MJ, Van den Bosch T, de Leeuw RA, Dueholm M, Exacoustos C, Valentin L, et al. Consensus on revised definitions of Morphological Uterus Sonographic Assessment (MUSA) features of adenomyosis: results of modified Delphi procedure. Ultrasound in obstetrics & gynecology : the official journal of the International Society of Ultrasound in Obstetrics and Gynecology. 2022;60(1):118-31.

27. Van den Bosch T, Dueholm M, Leone FP, Valentin L, Rasmussen CK, Votino A, et al. Terms, definitions and measurements to describe sonographic features of myometrium and uterine masses: a consensus opinion from the Morphological Uterus Sonographic Assessment (MUSA) group. Ultrasound in obstetrics & gynecology : the official journal of the International Society of Ultrasound in Obstetrics and Gynecology. 2015;46(3):284-98.

28. kommuner SfSro. Meddelande från styrelsen - Rekommendation

assisterad befruktning – dubbeldonation och

embryodonation. 2022.

29. Donnez J, Taylor HS, Marcellin L, Dolmans MM. Uterine fibroid-related infertility: mechanisms and management. Fertility and sterility. 2024.

30. Grimbizis GF, Gordts S, Di Spiezio Sardo A, Brucker S, De Angelis C, Gergolet M, et al. The ESHRE/ESGE consensus on the classification of female genital tract congenital anomalies. Human reproduction (Oxford, England). 2013;28(8):2032-44.

31. Adamson GD, Pasta DJ. Endometriosis fertility index: the new, validated endometriosis staging system. Fertility and sterility. 2010;94(5):1609-15.

32. Saridogan E, Becker CM, Feki A, Grimbizis GF, Hummelshoj L, Keckstein J, et al. Recommendations for the Surgical Treatment of Endometriosis. Part 1: Ovarian Endometrioma. Hum Reprod Open. 2017;2017(4):hox016.

33. Keckstein J, Becker CM, Canis M, Feki A, Grimbizis GF, Hummelshoj L, et al. Recommendations for the surgical treatment of endometriosis. Part 2: deep endometriosis. Hum Reprod Open. 2020;2020(1):hoaa002.

34. Psaroudakis D, Hirsch M, Davis C. Review of the management of ovarian endometriosis: paradigm shift towards conservative approaches. Current opinion in obstetrics & gynecology. 2014;26(4):266-74.

35. Cohen A, Almog B, Tulandi T. Sclerotherapy in the management of ovarian endometrioma: systematic review and meta-analysis. Fertility and sterility. 2017;108(1):117-24.e5.

36. Roman H, Pura I, Tarta O, Mokdad C, Auber M, Bourdel N, et al. Vaporization of ovarian endometrioma using plasma energy: histologic findings of a pilot study. Fertility and sterility. 2011;95(5):1853-6.e1-4.

37. Candiani M, Ferrari S, Bartiromo L, Schimberni M, Tandoi I, Ottolina J. Fertility Outcome after CO(2) Laser Vaporization versus Cystectomy in Women with Ovarian Endometrioma: A Comparative Study. Journal of minimally invasive gynecology. 2021;28(1):34-41.

38. Socialstyrelsen. 2024 [Available from: <https://www.socialstyrelsen.se/kunskapsstod-och-regler/regler-och-riktlinjer/nationell-hogspecialiserad-vard/arlig-uppfoljning/tillstandsomrade/endometrioskirurgi/>.

39. (SKR) SfSkor. Rekommendation

assisterad befruktning – dubbeldonation och

embryodonation. <https://skr.se/download/18.1e10e3a11847c6a2a5a20b51/1668778314464/WEBB-KOMB-14-2022-Meddelande-Rekommendation%20assisterad%20befruktning.pdf>: Sveriges kommuner och regioner (SKR); 2022-11-18.

40. Vincent K, Kennedy S, Stratton P. Pain scoring in endometriosis: entry criteria and outcome measures for clinical trials. Report from the Art and Science of Endometriosis meeting. Fertil Steril. 2010;93(1):62-7.

41. Rindos NB, Fulcher IR, Donnellan NM. Pain and Quality of Life after Laparoscopic Excision of Endometriosis. Journal of minimally invasive gynecology. 2020;27(7):1610-7.e1.

42. Poordast T, Alborzi S, Askary E, Tavabe MS, Najib FS, Salehi A, et al. Comparing the quality of life of endometriotic patients' before and after treatment with normal and infertile patients based on the EHP30 questionnaire. . BMC Womens Health. 2022;22(1):553.

43. Tiringer D, Pedrini AS, Gstoettner M, Husslein H, Kuessel L, Perricos A, et al. Evaluation of quality of life in endometriosis patients before and after surgical treatment using the EHP30 questionnaire. BMC Womens Health. 2022;22(1):538.

44. Alsbjerg B, Kesmodel US, Humaidan P. Endometriosis patients benefit from high serum progesterone in hormone replacement therapy-frozen embryo transfer cycles: a cohort study. Reproductive biomedicine online. 2023;46(1):92-8.

45. Labarta E, Mariani G, Holtmann N, Celada P, Remohí J, Bosch E. Low serum progesterone on the day of embryo transfer is associated with a diminished ongoing pregnancy rate in oocyte donation cycles after artificial endometrial preparation: a prospective study. Human reproduction (Oxford, England). 2017;32(12):2437-42.

46. Sweden Q-I. Fertilitetsbehandlingar i Sverige Årsrapport 2023 (Fertility treatments in Sweden 2023 report) 2023 [Available from: <https://www.medscinet.com/QIVF/uploads/hemsida/%C3%85rsrapport%202023%20Final.pdf>.

47. Márki G, Vásárhelyi D, Rigó A, Kaló Z, Ács N, Bokor A. Challenges of and possible solutions for living with endometriosis: a qualitative study. BMC Womens Health. 2022;22(1):20.

48. Matsuzaki S, Canis M, Pouly JL, Rabischong B, Botchorishvili R, Mage G. Relationship between delay of surgical diagnosis and severity of disease in patients with symptomatic deep infiltrating endometriosis. Fertility and sterility. 2006;86(5):1314-6; discussion 7.

49. Söderman L, Edlund M, Marions L. Prevalence and impact of dysmenorrhea in Swedish adolescents. Acta obstetricia et gynecologica Scandinavica. 2019;98(2):215-21.

# APPENDIX

Intra- and postoperative complications

Clavien Dindo (22)


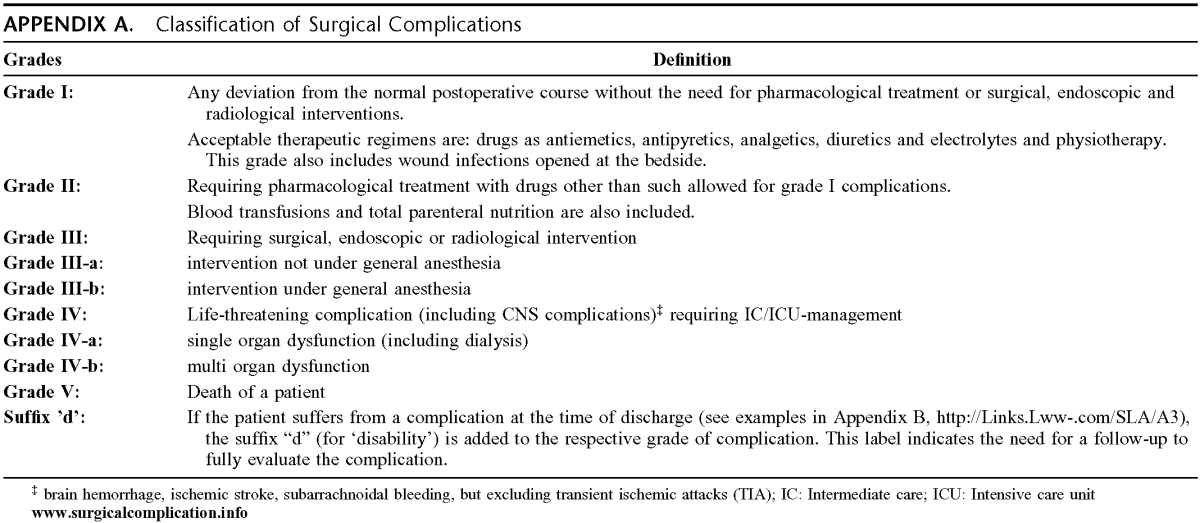


Classic (21)


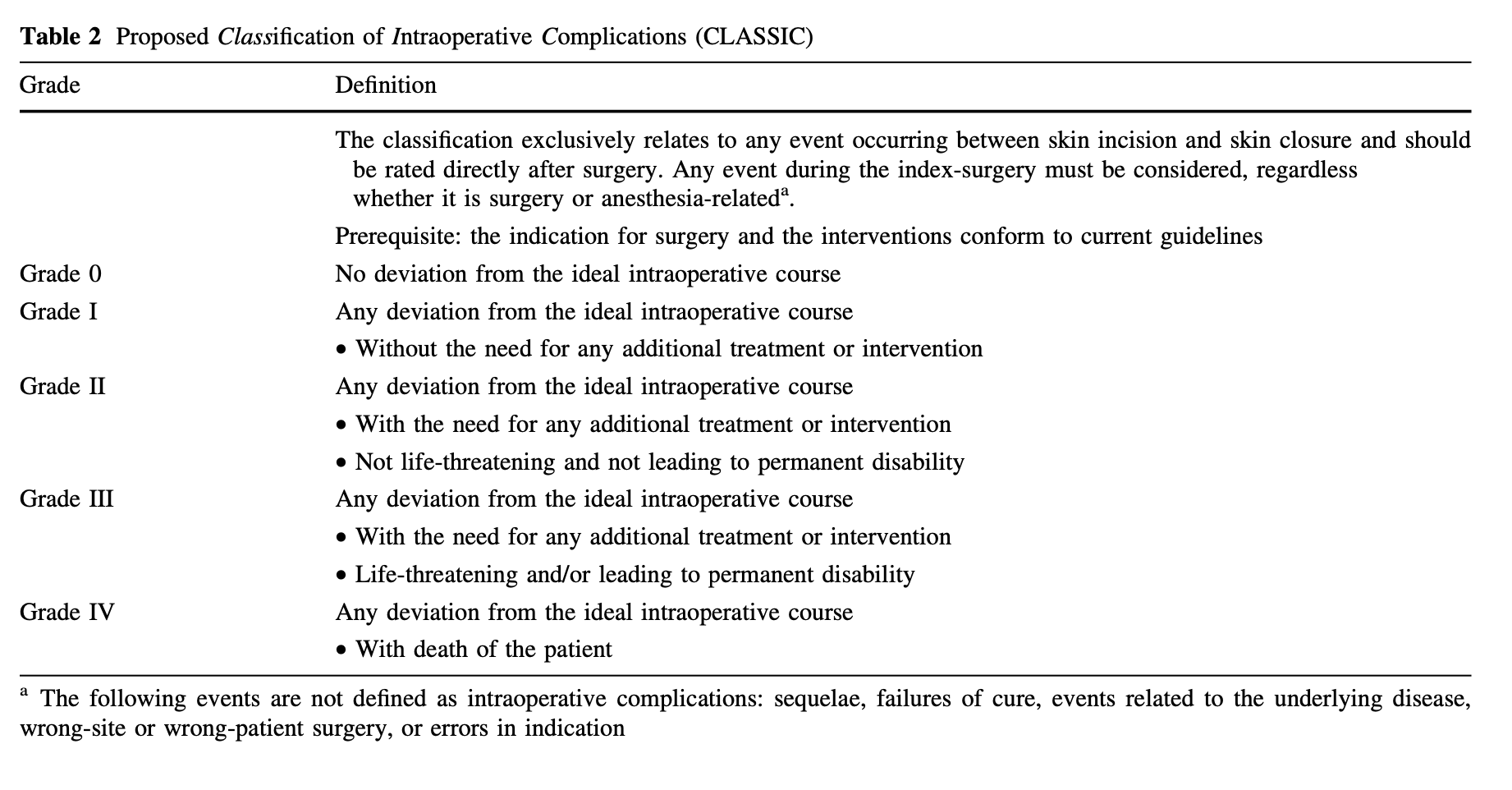


Endometriosis Health Profile EHP-30

ENDOMETRIOS HÄLSOPROFILFORMULÄR
(EHP-30)

DEL 1: HUVUDFORMULÄR

Hur ofta under de senaste 4 veckorna
har du på grund av din endometrios…

|  | | | Aldrig | Sällan | Ibland | Ofta | Alltid |
| --- | --- | --- | --- | --- | --- | --- | --- |
| 1. | Inte kunnat vara med på sociala evenemang på grund av smärtan? |  |  |  |  |  |  |
| 2. | Inte kunnat utföra sysslor  i hemmet på grund av smärtan? |  |  |  |  |  |  |
| 3. | Haft svårt att stå på grund av smärtan? |  |  |  |  |  |  |
| 4. | Haft svårt att sitta på grund av smärtan? |  |  |  |  |  |  |
| 5. | Haft svårt att gå på grund av smärtan? |  |  |  |  |  |  |
| 6. | Haft svårt att motionera eller  utöva de fritidsaktiviteter du velat göra på grund av smärtan? |  |  |  |  |  |  |
| 7. | Tappat aptiten och/eller inte kunnat äta på grund av smärtan? |  |  |  |  |  |  |

Var vänlig kontrollera att du har ***kryssat i en ruta för varje fråga***innan du går vidare till nästa sida.

Hur ofta under de senaste 4 veckorna
har du på grund av din endometrios…

|  | | | Aldrig | Sällan | Ibland | Ofta | Alltid |
| --- | --- | --- | --- | --- | --- | --- | --- |
| 8. | Inte kunnat sova ordentligt på grund av smärtan? |  |  |  |  |  |  |
| 9. | Varit tvungen att gå till sängs/ligga ner på grund av smärtan? |  |  |  |  |  |  |
| 10. | Inte kunnat göra de saker du velat göra på grund av smärtan? |  |  |  |  |  |  |
| 11. | Känt att du inte kunnat hantera smärtan? |  |  |  |  |  |  |
| 12. | Känt dig allmänt krasslig? |  |  |  |  |  |  |
| 13. | Känt dig frustrerad över att dina symtom inte blir bättre? |  |  |  |  |  |  |
| 14. | Känt dig frustrerad över att du inte kan kontrollera dina symtom? |  |  |  |  |  |  |

Var vänlig kontrollera att du har ***kryssat i en ruta för varje fråga***innan du går vidare till nästa sida.

Hur ofta under de senaste 4 veckorna
har du på grund av din endometrios…

|  | | | Aldrig | Sällan | Ibland | Ofta | Alltid |
| --- | --- | --- | --- | --- | --- | --- | --- |
| 15. | Känt att du inte kan glömma dina symtom? |  |  |  |  |  |  |
| 16. | Känt det som om dina symtom  styr ditt liv? |  |  |  |  |  |  |
| 17. | Känt det som om dina symtom  tar ifrån dig ditt liv? |  |  |  |  |  |  |
| 18. | Känt dig nedstämd? |  |  |  |  |  |  |
| 19. | Känt dig gråtmild/tårögd? |  |  |  |  |  |  |
| 20. | Känt dig eländig? |  |  |  |  |  |  |
| 21. | Haft humörsvängningar? |  |  |  |  |  |  |
| 22. | Känt dig på dåligt humör eller lättretad? |  |  |  |  |  |  |

Var vänlig kontrollera att du har ***kryssat i en ruta för varje fråga***innan du går vidare till nästa sida.

Hur ofta under de senaste 4 veckorna
har du på grund av din endometrios…

|  | | | Aldrig | Sällan | Ibland | Ofta | Alltid |
| --- | --- | --- | --- | --- | --- | --- | --- |
| 23. | Känt dig våldsam eller aggressiv? |  |  |  |  |  |  |
| 24. | Känt att du inte kunnat tala om  för andra människor hur du mår? |  |  |  |  |  |  |
| 25. | Känt att andra människor inte förstår vad du går igenom? |  |  |  |  |  |  |
| 26. | Känt det som att andra människor tycker att du gnäller? |  |  |  |  |  |  |
| 27. | Känt dig ensam? |  |  |  |  |  |  |
| 28. | Känt dig frustrerad över att du  inte alltid kan använda de kläder du önskar? |  |  |  |  |  |  |
| 29. | Känt att ditt utseende påverkats? |  |  |  |  |  |  |
| 30. | Saknat självförtroende? |  |  |  |  |  |  |

Var vänlig kontrollera att du har ***kryssat i en ruta för varje fråga***

innan du går vidare till nästa sida.

DEL 2: FRÅGEFORMULÄR I FLERA AVSNITT

**Avsnitt A:**Dessa frågor avser den inverkan endometriosen har haft på ditt arbete **under de senaste 4 veckorna.** Om du inte har haft något betalt arbete eller volontärarbete under de senaste 4 veckorna, kryssa i här  och gå vidare till avsnitt B.

Hur ofta under de senaste 4 veckorna
har du på grund av din endometrios...

|  | | | Aldrig | Sällan | Ibland | Ofta | Alltid |
| --- | --- | --- | --- | --- | --- | --- | --- |
| 1. | Behövt vara borta från jobbet på grund av smärtan? |  |  |  |  |  |  |
| 2. | Inte kunnat utföra vissa arbetsuppgifter på grund av smärtan? |  |  |  |  |  |  |
| 3. | Känt dig generad över dina symtom på jobbet? |  |  |  |  |  |  |
| 4. | Haft dåligt samvete för att du varit borta från jobbet? |  |  |  |  |  |  |
| 5. | Känt oro för att inte klara av ditt arbete? |  |  |  |  |  |  |

Var vänlig kontrollera att du har ***kryssat i*** ***en ruta vid varje fråga***

innan du går vidare till nästa sida.

**Avsnitt B:**Dessa frågor avser den inverkan endometriosen har haft på förhållandet till ditt/dina barn **under de senaste 4 veckorna.** Om du inte har några barn, kryssa i här
och gå vidare till avsnitt C.

Hur ofta under de senaste 4 veckorna
har du på grund av din endometrios...

|  | | | Aldrig | Sällan | Ibland | Ofta | Alltid |
| --- | --- | --- | --- | --- | --- | --- | --- |
| 1. | Tyckt att du hade svårt att ta hand om ditt/dina barn? |  |  |  |  |  |  |
| 2. | Inte kunnat leka med ditt/dina barn? |  |  |  |  |  |  |

Var vänlig kontrollera att du har ***kryssat i*** ***en ruta vid varje fråga***

innan du går vidare till nästa sida.

**Avsnitt C:**Dessa frågor avser den inverkan endometriosen har haft på dina sexuella relationer **under de senaste 4 veckorna**.

Hur ofta under de senaste 4 veckorna
har du på grund av din endometrios...

|  | | | Aldrig | Sällan | Ibland | Ofta | Alltid |
| --- | --- | --- | --- | --- | --- | --- | --- |
| 1. | Känt smärta under eller efter samlag? ***Om ej tillämpligt***  ***kryssa i här*** |  |  |  |  |  |  |
| 2. | Känt oro över att ha samlag på grund av smärtan? ***Om ej tillämpligt***  ***kryssa i här*** |  |  |  |  |  |  |
| 3. | Undvikit samlag på grund av smärtan? ***Om ej tillämpligt***  ***kryssa i här*** |  |  |  |  |  |  |
| 4. | Haft dåligt samvete för att du inte har velat ha samlag? ***Om ej tillämpligt***  ***kryssa i här*** |  |  |  |  |  |  |
| 5. | Känt dig frustrerad över att du inte kan njuta av samlag? ***Om ej tillämpligt***  ***kryssa i här*** |  |  |  |  |  |  |

Var vänlig kontrollera att du har ***kryssat i*** ***en ruta vid varje fråga***

innan du går vidare till nästa sida.

**Avsnitt D:**Dessa frågor avser hur du har känt **under de senaste 4 veckorna** när det gäller läkarna**.**
Om detta avsnitt inte är tillämpligt, kryssa i här  och gå vidare till avsnitt E.

Hur ofta under de senaste 4 veckorna
har du på grund av din endometrios...

|  | | | Aldrig | Sällan | Ibland | Ofta | Alltid |
| --- | --- | --- | --- | --- | --- | --- | --- |
| 1. | Känt att den eller de läkare som du har träffat inte hjälper dig på något sätt? |  |  |  |  |  |  |
| 2. | Känt att läkaren eller läkarna anser att du bara inbillar dig? |  |  |  |  |  |  |
| 3. | Känt dig frustrerad över läkarens eller läkarnas bristande kunskap om endometrios? |  |  |  |  |  |  |
| 4. | Känt dig som om du i onödan upptar läkarens eller läkarnas tid? |  |  |  |  |  |  |

Var vänlig kontrollera att du har ***kryssat i*** ***en ruta vid varje fråga***

innan du går vidare till nästa sida.

**Avsnitt E:**Dessa frågor avser hur du har känt **under de senaste 4 veckorna** när det gäller din behandling mot endometrios. Med behandling menar vi eventuella kirurgiska ingrepp eller **receptbelagda** läkemedel mot din endometrios.
Om detta avsnitt inte är tillämpligt, kryssa i här  och gå vidare till avsnitt F.

Hur ofta under de senaste 4 veckorna
har du på grund av din endometrios...

|  | | | Aldrig | Sällan | Ibland | Ofta | Alltid |
| --- | --- | --- | --- | --- | --- | --- | --- |
| 1. | Känt dig frustrerad över att behandlingen inte fungerar? |  |  |  |  |  |  |
| 2. | Tyckt att det har varit svårt att hantera biverkningarna av behandlingen? |  |  |  |  |  |  |
| 3. | Känt dig irriterad över hur mycket behandling du har behövt genomgå? |  |  |  |  |  |  |

Var vänlig kontrollera att du har ***kryssat i*** ***en ruta vid varje fråga***

innan du går vidare till nästa sida.

**Avsnitt F:**Dessa frågor avser dina problem med att bli gravid **under de senaste 4 veckorna.**
Om detta avsnitt inte är tillämpligt, kryssa i här .

Hur ofta under de senaste 4 veckorna
har du på grund av din endometrios...

|  | | | Aldrig | Sällan | Ibland | Ofta | Alltid |
| --- | --- | --- | --- | --- | --- | --- | --- |
| 1. | Känt dig orolig av att du möjligen inte ska kunna få barn/fler barn? |  |  |  |  |  |  |
| 2. | Känt dig otillräcklig därför att du kanske inte kan få/inte har kunnat få barn/fler barn. |  |  |  |  |  |  |
| 3. | Känt dig deprimerad av att du möjligen inte ska kunna få barn/fler barn? |  |  |  |  |  |  |
| 4. | Känt att att du möjligen inte kan bli gravid har skapat spänningar i ditt förhållande? |  |  |  |  |  |  |

Var vänlig kontrollera att du har ***kryssat i en ruta vid varje fråga.***

EQ5D

#ENZIAN and AAGL endometriosis staging


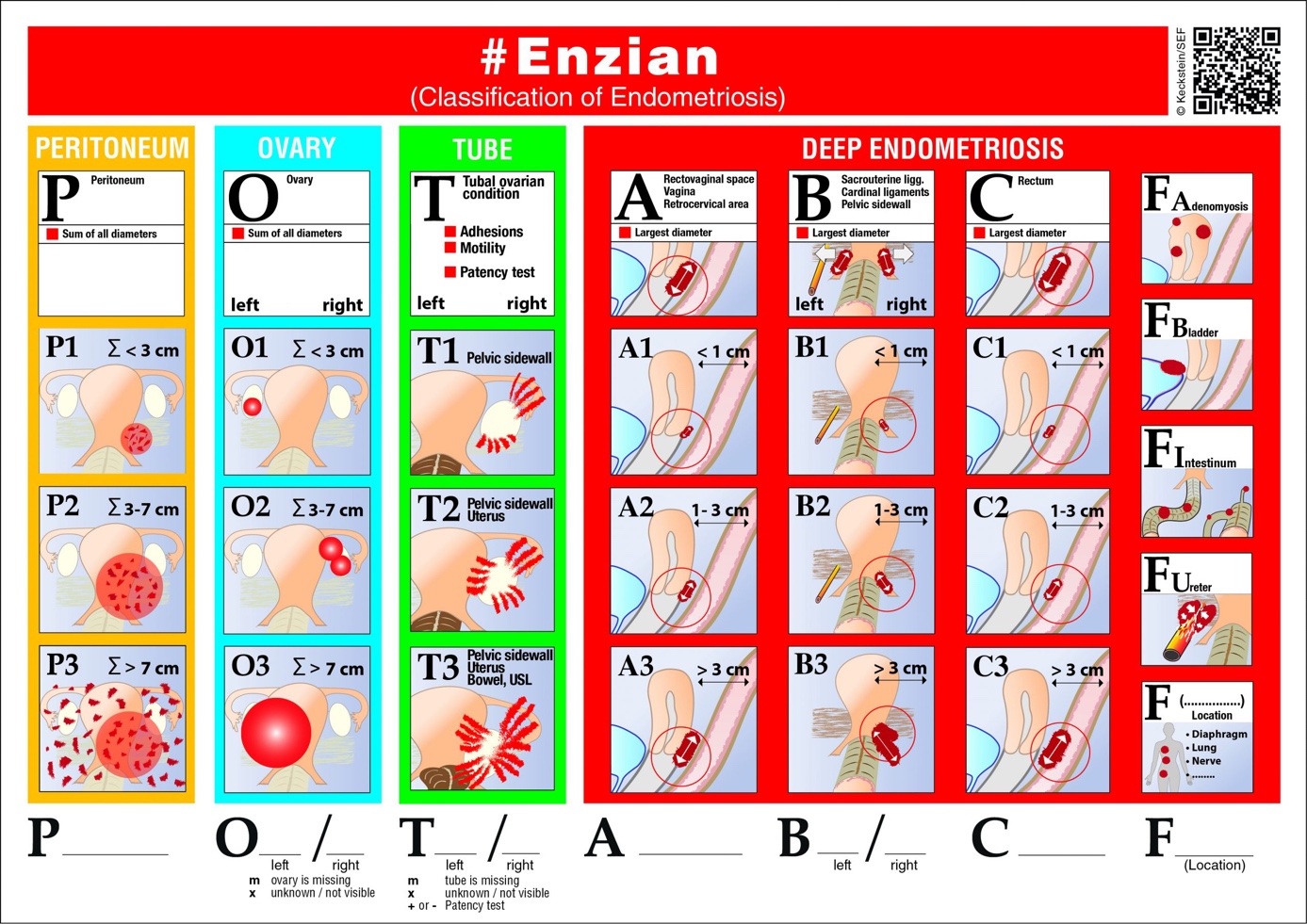


Can be used with a mobile application


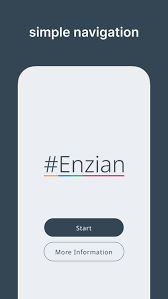

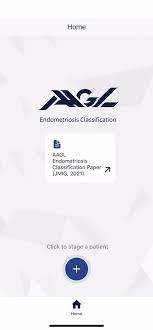


FIGO leiomyoma classification system


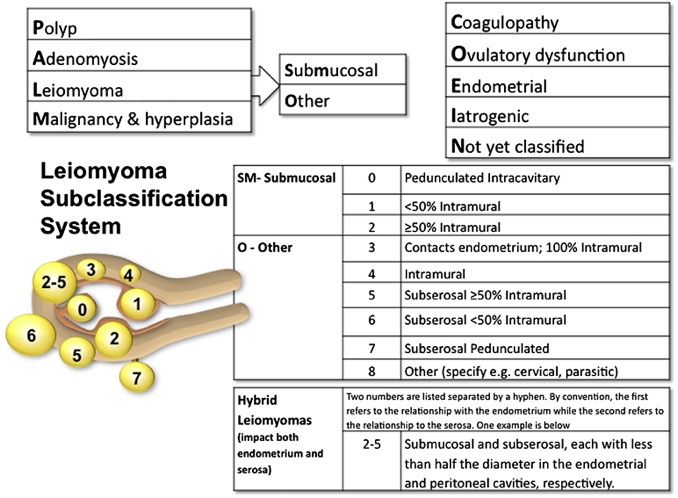


KVÅ codes

**Posterior compartment**

JEA00 Appendektomi

JEA01 Laparoskopisk appendektomi

JFA70 Enterorafi

JFA71 Laparoskopisk enterorafi

JFA73 Exstirpation av lokal förändring i tunntarm

JFA74 Laparoskopisk exstirpation av lokal förändring i tunntarm

JFA80 Kolorafi

JFA81 Laparoskopisk kolorafi

JFA83 Exstirpation av lokal förändring i kolon

JFA84 Laparoskopisk exstirpation av lokal förändring i kolon

JFB 50 Annan kolonresektion

JFB51 Annan laparoskopisk kolonresektion

JGB 96 Annan resektion av rektum

JGB97 Annan laparoskopisk resektion av rektum

JFB00 Tunntarmsresektion

JFB01 Laparoskopisk tunntarmsresektion

JFB20 Ileocekalresektion

JFB21 Laparoskopisk ileocekalresektion

JFB46 Resektion av colon sigmoideum

JFB47 Laparoskopisk resektion av colon sigmoideum

JFB53 Resektion av colon sigmoideum med samtidig resektion av del av rektum

JFB54 Laparoskopisk resektion av colon sigmoideum med samtidig resektion av del av rektum

LEW97 Annan laparoskopisk operation av vagina

LEB10 Exstirpation av förändring i vagina

LDB00 Exstirpation av förändring i portio eller cervix uteri

LCG30 Resektion/extirpation av sakrouterin ligament

LCG31 Laparoskopisk resektion/extirpation av sakrouterin ligament

**Anterior compartment**

KCA00 Exploration av urinblåsa

KCD10 Öppen resektion av urinblåsa

KCD96 Annan resektion eller destruktion i urinblåsa

KCH00 Sutur av urinblåsa

KBH00 Sutur av uretär

KBA00 Exploration av uretär

KBH50 Lösning av fixerad uretär

KBH 20 Ureterocystostomi

KBD00 Resektion av uretär

KBH10 Ureteroureterostomi, kontralateral

LCF96 Annan exstirpation av förändring i parametrium

LCF97 Annan laparoskopisk exstirpation av förändring i parametrium

**Adnexal surgery**

LBE00 Salpingektomi

LBE01 Laparoskopisk salpingektomi

LBD00 Tubarresektion

LBD01 Laparoskopisk tubarresektion

LAG00 Ovariolys

LAG01 Laparoskopisk ovariolys

LAF00 Unilateral salpingo-ooforektomi

LAF01 Laparoskopisk unilateral salpingo-ooforektomi

LAF10 Bilateral salpingo-ooforektomi

LAF11 Laparoskopisk bilateral salpingo-ooforektomi

LAE10 Unilateral ooforektomi

LAE11 Laparoskopisk unilateral ooforektomi

LAE20 Bilateral ooforektomi

LAE21 Laparoskopisk bilateral ooforektomi

LAD00 Resektion av ovarium

LAD01 Laparoskopisk resektion av ovarium

LAC00 Enukleation av ovarialcysta

LAC01 Laparoskopisk enukleation av ovarialcysta

LAC10 Fenestrering av ovarialcysta

LAC11 Laparoskopisk fenestrering av ovarialcysta

LAC20 Destruktion av förändring i ovarium Inkl. koagulation av endometrios,

LAC21 Laparoskopisk destruktion av förändring i ovarium

| **Medicinska Födelseregistret** |  |
| --- | --- |
| **Variabel** | **Klartext** |
| APGAR1 | Apgarbedömning vid 1 minut |
| APGAR10 | Apgarbedömning vid 10 minuter |
| APGAR5 | Apgarbedömning vid 5 minuter |
| AR | År |
| ARBETE | Arbete |
| BFODDAT | Barnets födelsedatum (Endast år-mån) |
| BLANGDF2 | Barnets längd |
| BORDF2 | Börd |
| BORDNRF2 | Bördnummer och antal vid flerbörd |
| BPSMDAT | Beräknad förlossning utifrån första dagen i senaste menstruationen |
| BPULDAT | Beräknad förlossning enl. ultraljudsundersökning |
| BVIKT | Barnets födelsevikt |
| CIVIL | Moderns civilstånd |
| DDAGAR | Dödsålder i dagar, endast nyföddhetsperioden |
| DODFOD | Dödfödd enl. FV2 |
| ELEKAKUT | Elektiv eller akut sectio |
| FAMSIT | Familjesituation |
| FBARN | Friskt barn |
| FLINDUKT | Förlossningen startar med induktion |
| FLSPONT | Förlossningen startar spontant |
| GDIAG1-GDIAG4 | Diagnos/åtgärd 1-4 under graviditet |
| GRDBS | Graviditetslängd bästa skattning, dagar |
| GRMETOD | Graviditetslängd, metod för skattning |
| GRVBS | Graviditetslängd bästa skattning, veckor |
| GRVFV | Graviditetslängd, fullb. veckor |
| KON | Barnets kön |
| LKF LK | Moderns folkbokföringsort (Endast län och kommun) |
| MALDER | Moderns ålder |
| MDIAG1-MDIAG12 | Moderns diagnos 1-12 |
| MFODDAT | Moderns födelsedatum (Endast år-mån) |
| MISSB | Finns missbildningsdiagnos |
| MLANGD | Moderns längd (cm) |
| MLGA | Tung för tiden, LGA |
| MSGA | Lätt för tiden, SGA |
| MVIKT | Vikt (kg) vid inskrivning till MHV |
| MVIKTFV | Modern vikt (kg) vid förlossning |
| NJURSJUK | Kronisk njursjukdom |
| OFRIABEF | Åtgärd vid ofri. barnlöshet: assisterad befruktning |
| OFRIANN | Åtgärd vid ofri. barnlöshet: annan |
| OFRIBARN | Ofrivillig barnlöshet (år) |
| OFRIIATG | Åtgärd vid ofri. barnlöshet: ingen |
| OFRIICSI | Åtgärd vid ofri. barnlöshet: ICSI |
| OFRIKIRU | Åtgärd vid ofri. barnlöshet: kirurgi |
| OFRISTIM | Åtgärd vid ofri. barnlöshet: ovulation stimulering |
| PARITET | Paritet, barnets ordningsnummer |
| PARITET_F | Paritet, ordningsnummer för förlossning |
| PSTOPDAT | Upphört med p-piller |
| ROK0-ROK2 | Rökning 3 månader före / vid inskrivning till MHV / vecka 30-32 |
| SECAVSL | Förlossningen avslutas med kejsarsnitt |
| SECFORE | Förlossningen startas med kejsarsnitt |
| SECMARK | Kejsarsnitt, markering |
| SECTIO | Sectio- elektivt eller ej elektivt |
| SFINKTER | Bristningar - sfinkter |
| SMDAT | Senaste menstruation |
| SNUS0-SNUS2 | Snusning 3 månader före / vid inskrivning till MHV / vecka 30-32 |
| SPUTDAT | Spiral uttagen |
| SUGKLOCK | Förlossningen avslutas med sugklocka |
| TANG | Förlossningen avslutas med tång |
| TID7DOD | Antal döda barn inom 0-6 dygn |
| TIDDODF | Antal dödfödda barn |
| TIDLEVF | Antal levande födda barn |
| TIDSDOD | Antal senare döda barn |
| TIDSPOAB | Antal tidigare spontan abort |
| TIDXGRAV | Antal X-graviditeter |
| TSECAR | Tidigare sectio, år |
| TSECTIO | Tidigare sectio |
| VAGINAL | Förlossningen avslutas vaginalt |


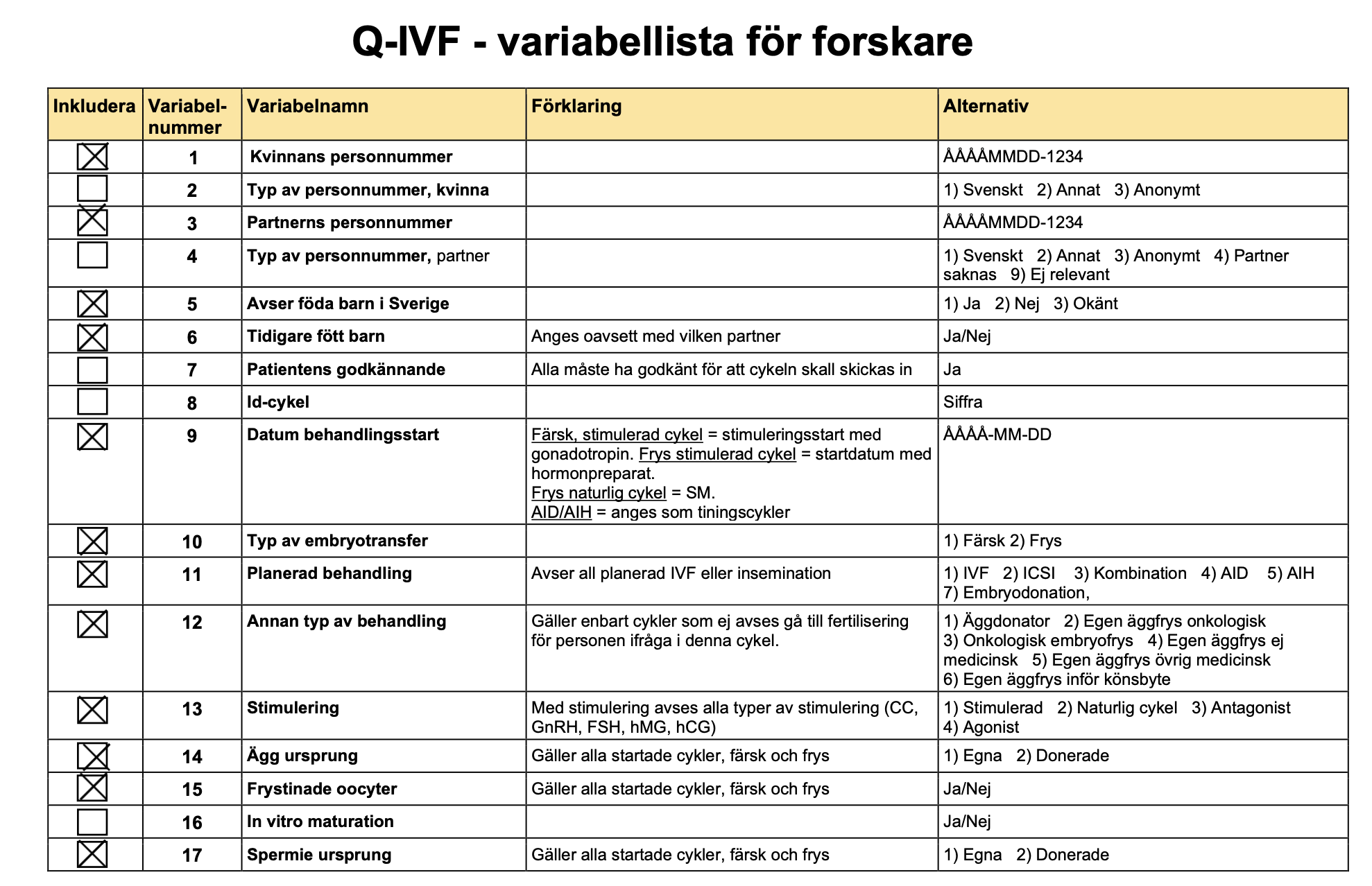


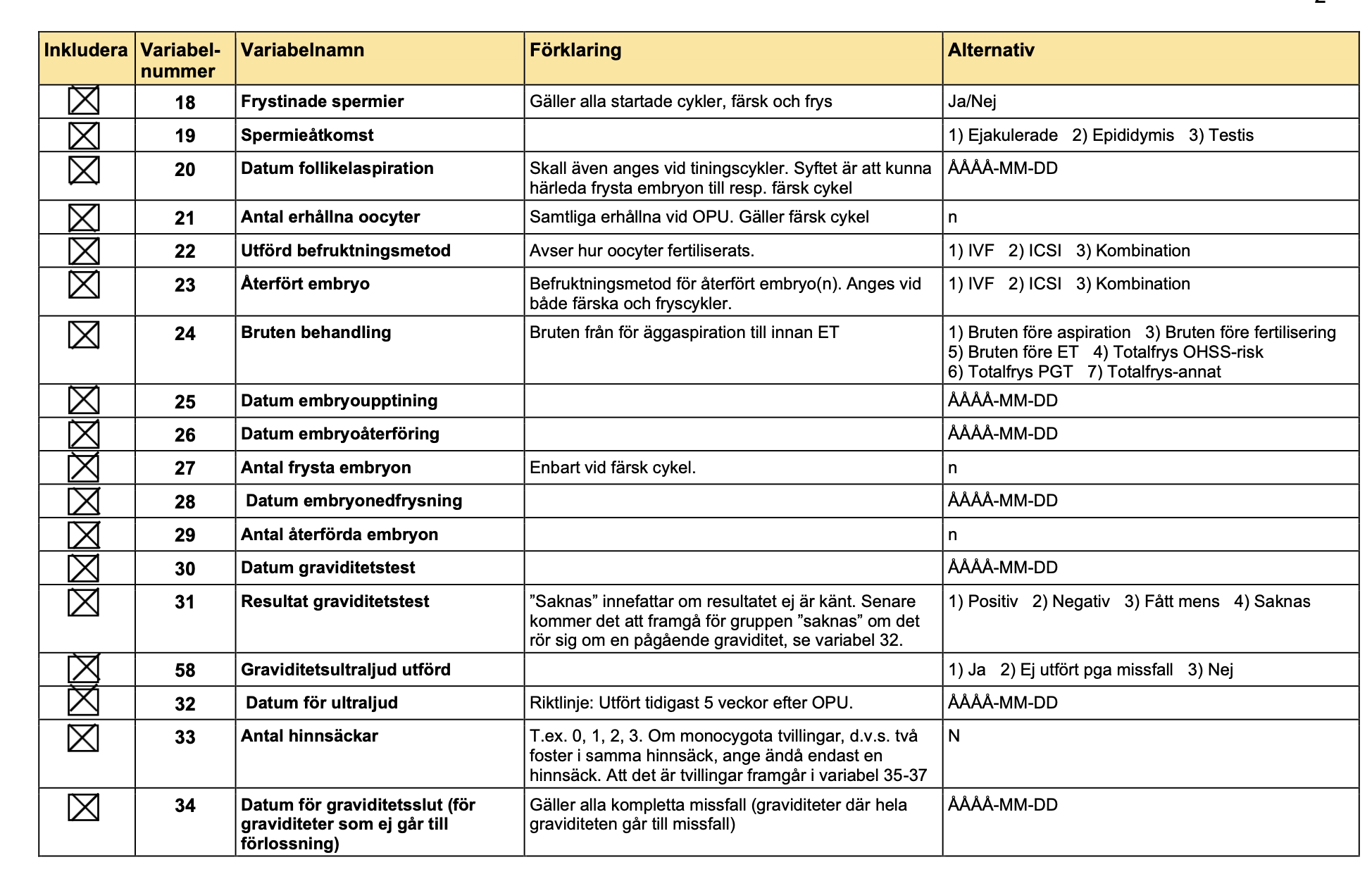


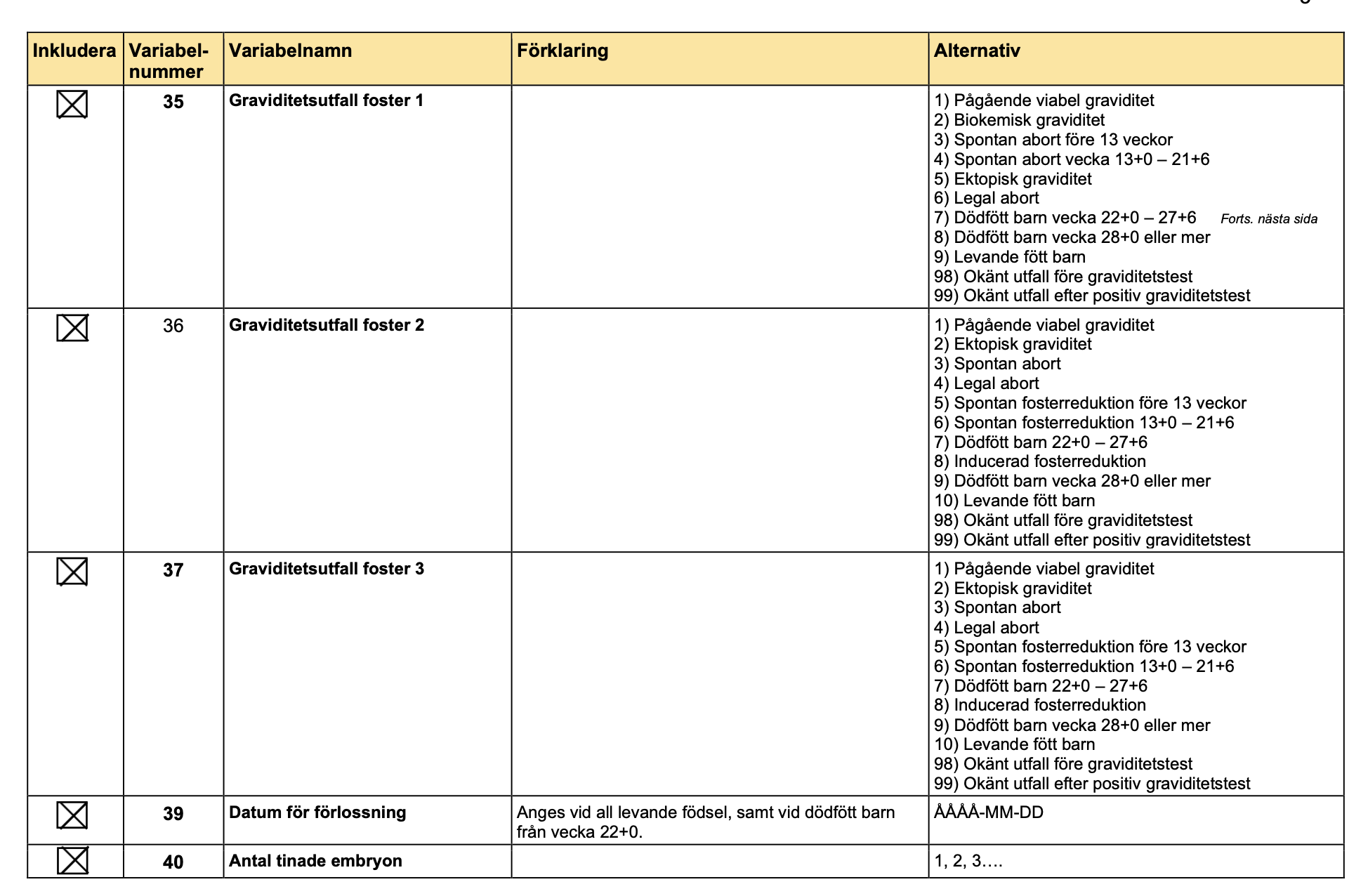


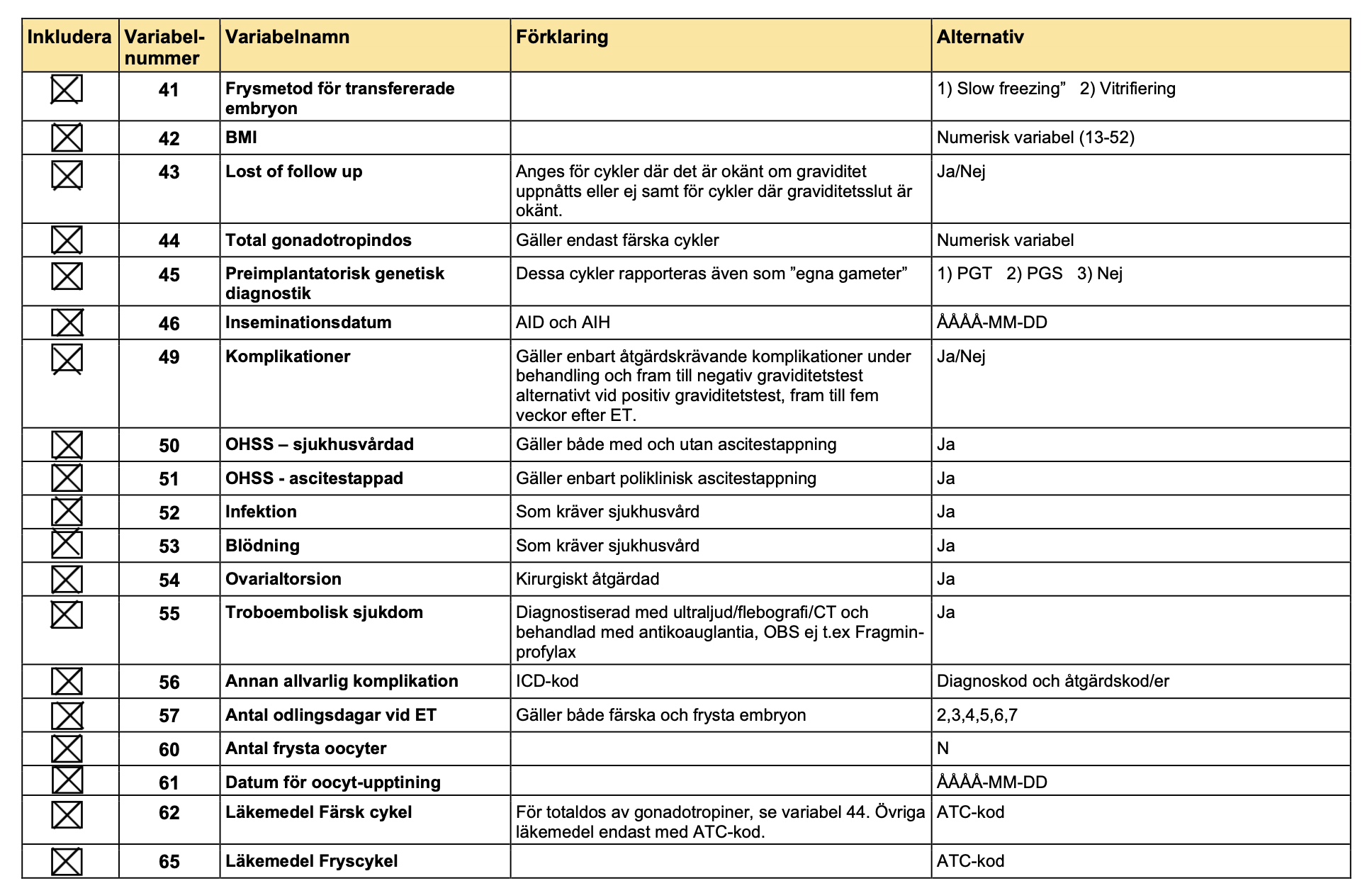


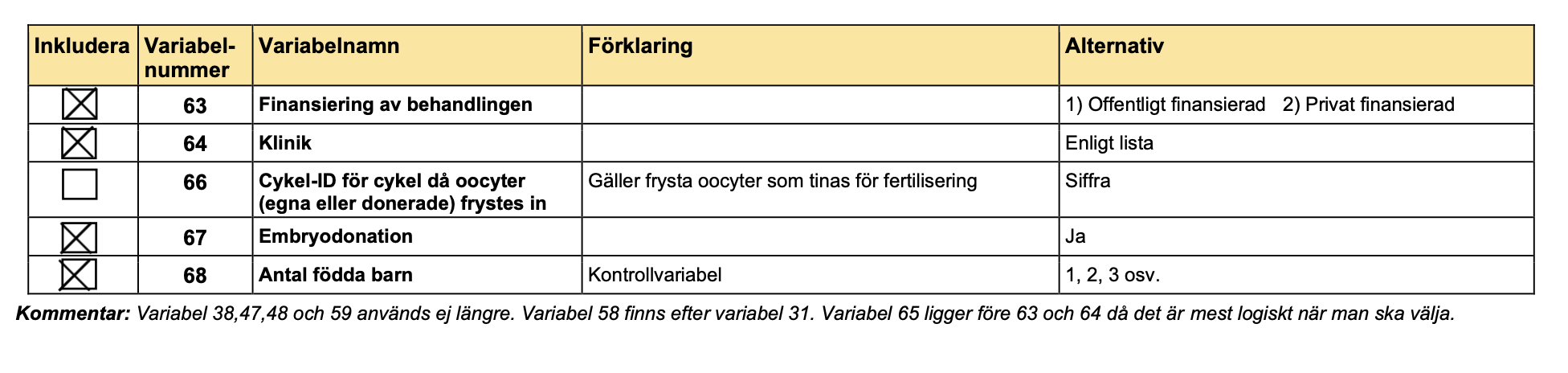


**Variabler från Gynop registret:**


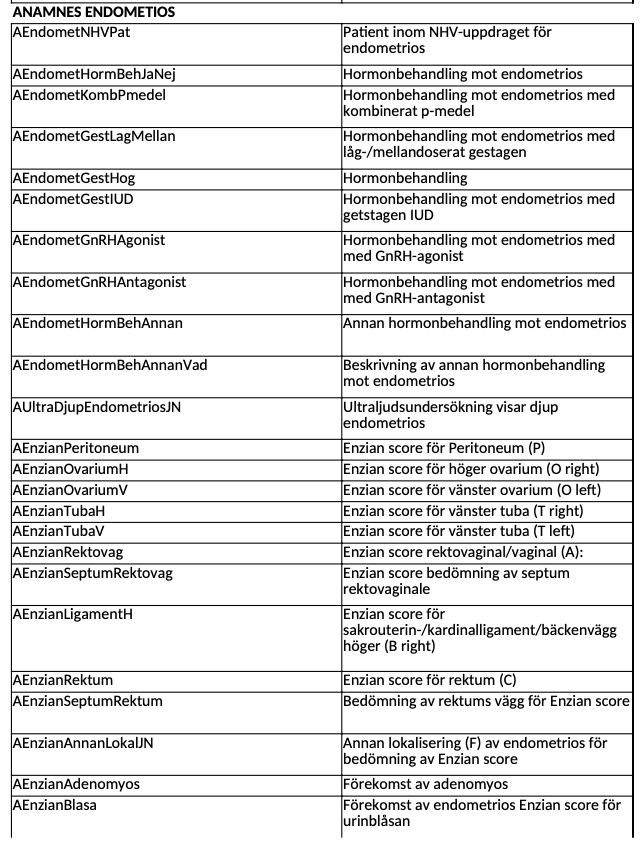


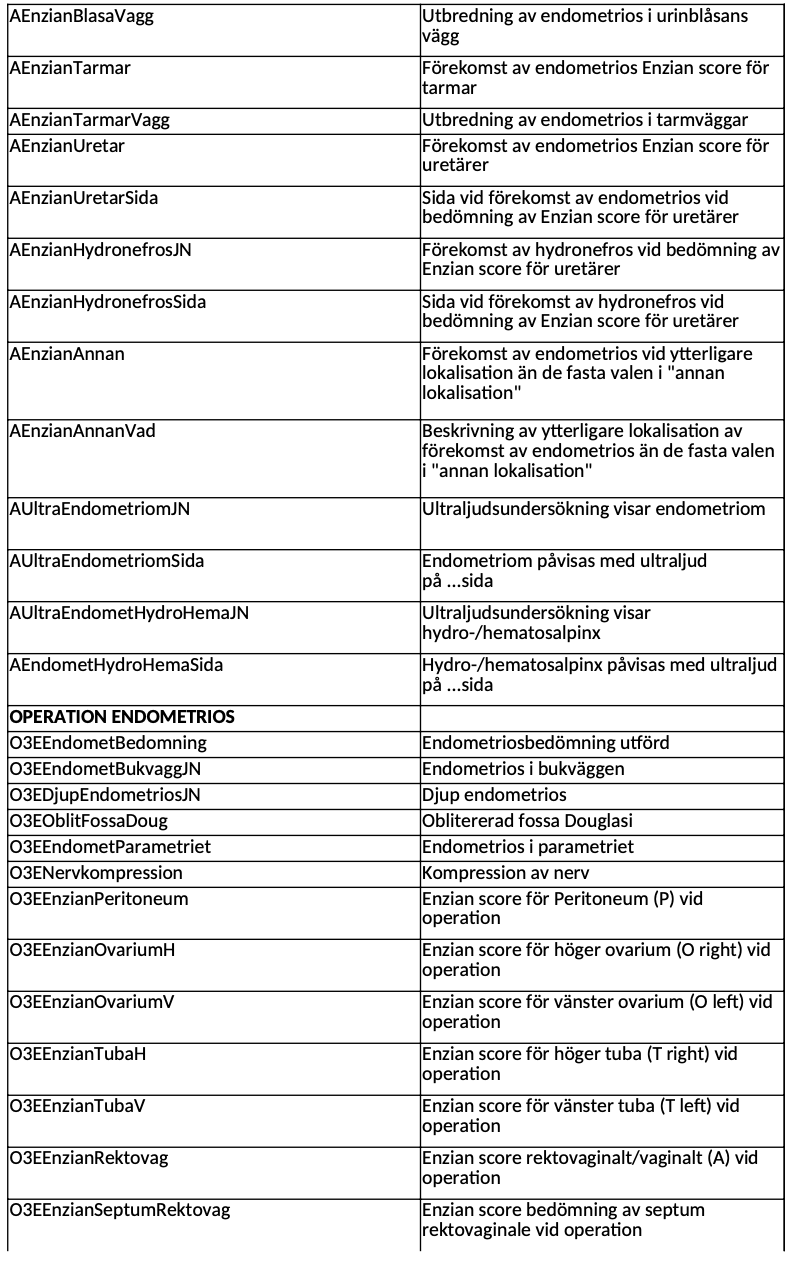


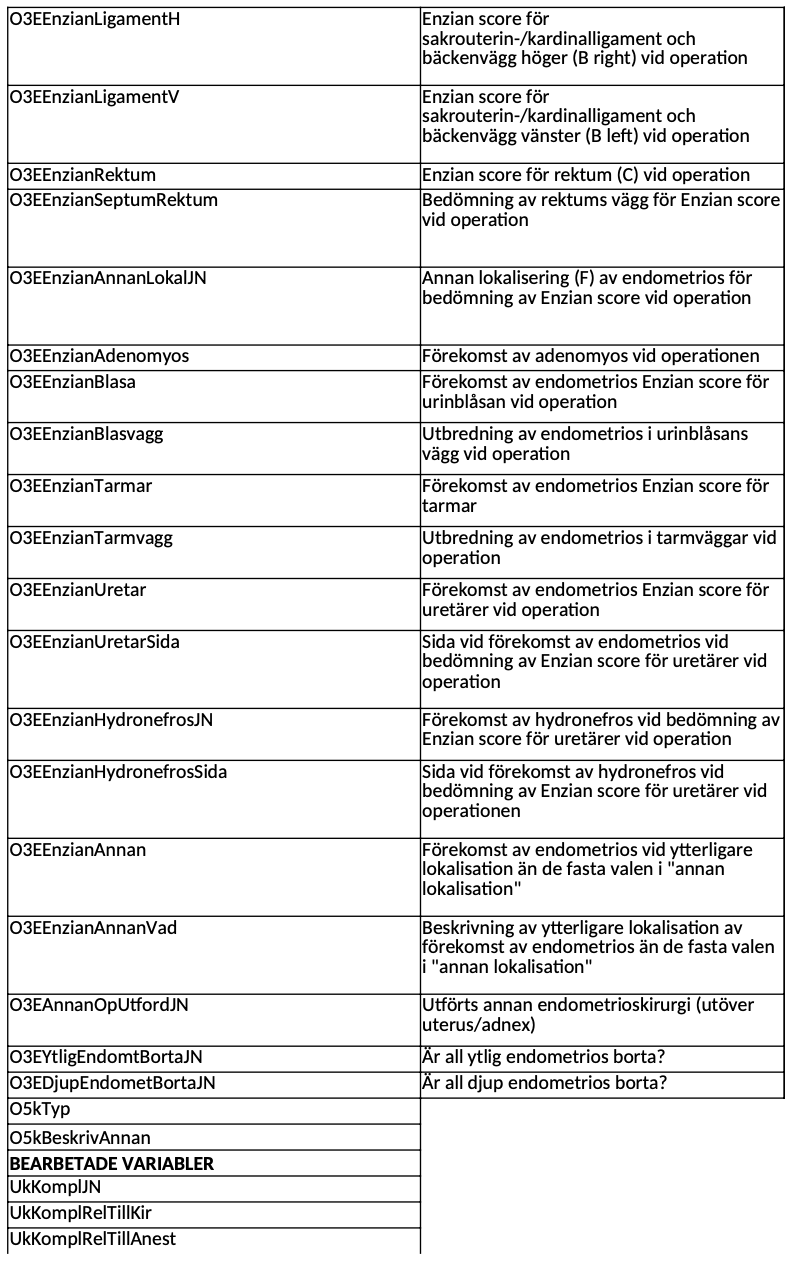


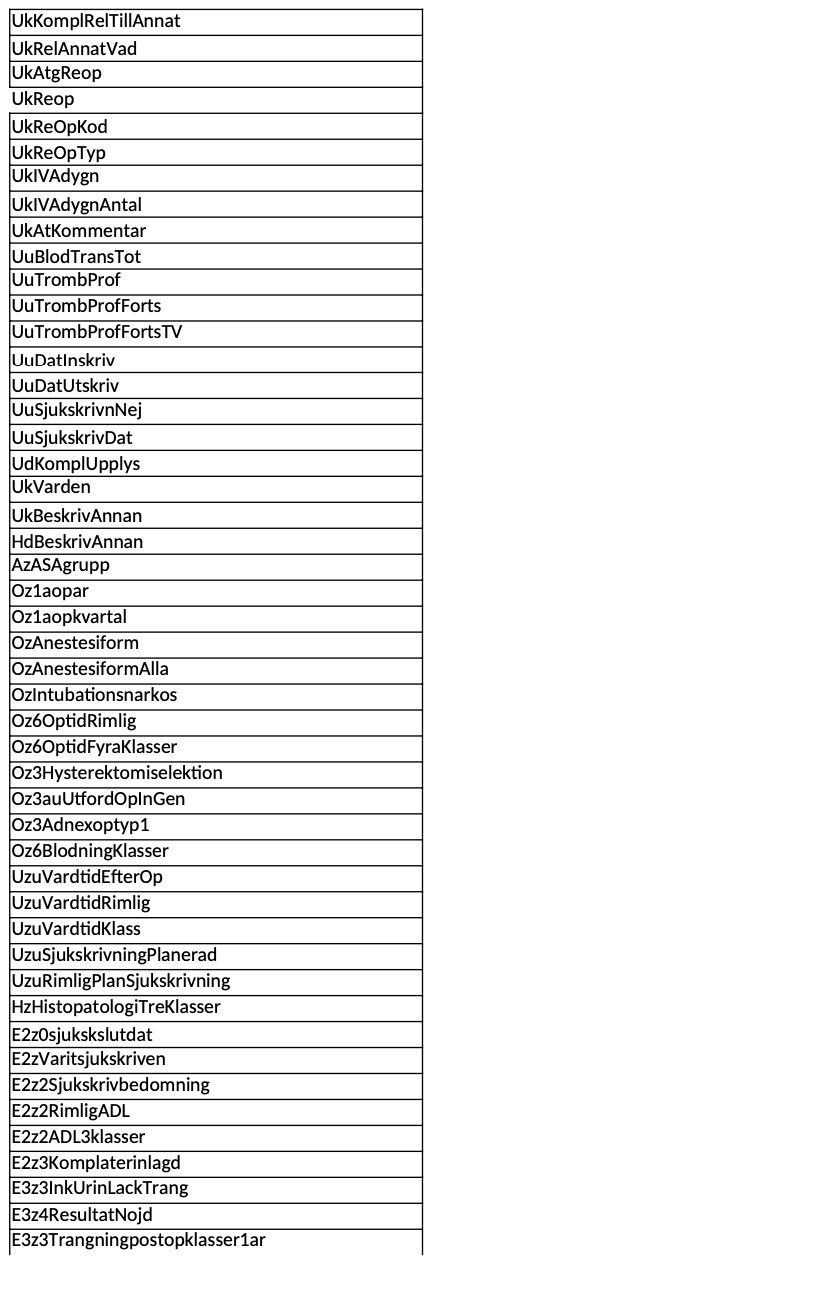


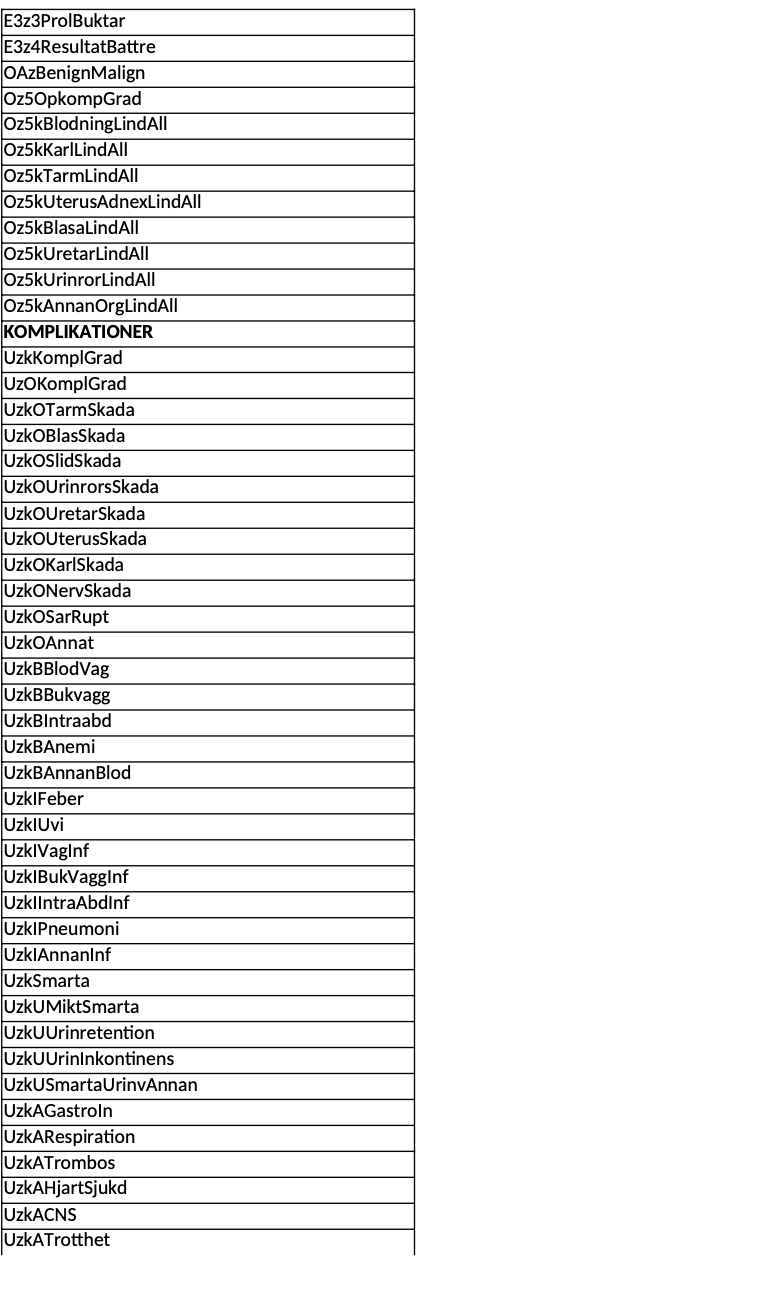


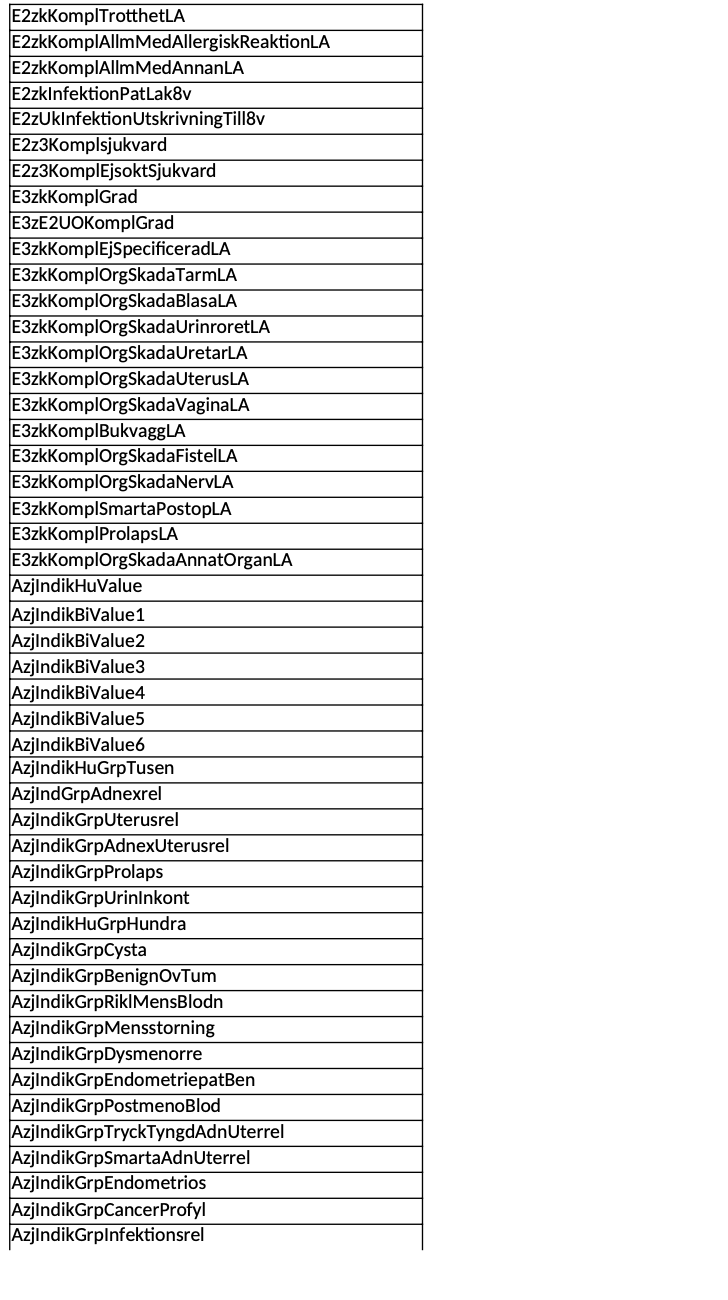


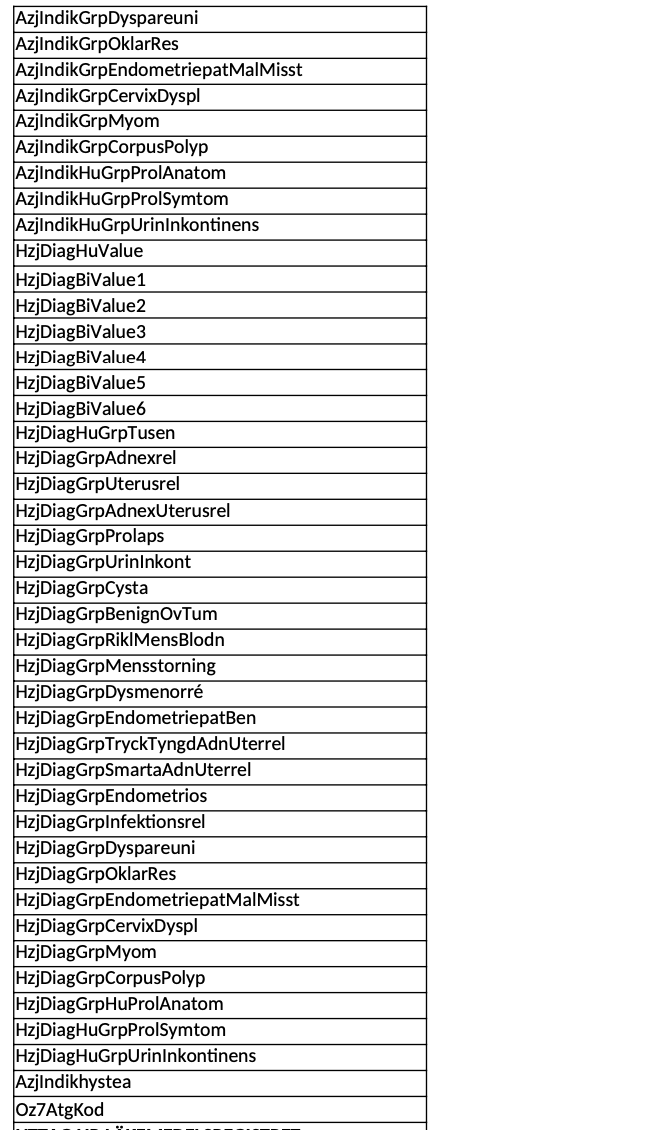


VARIABEL LISTA GRAVIDITETSREGISTRET


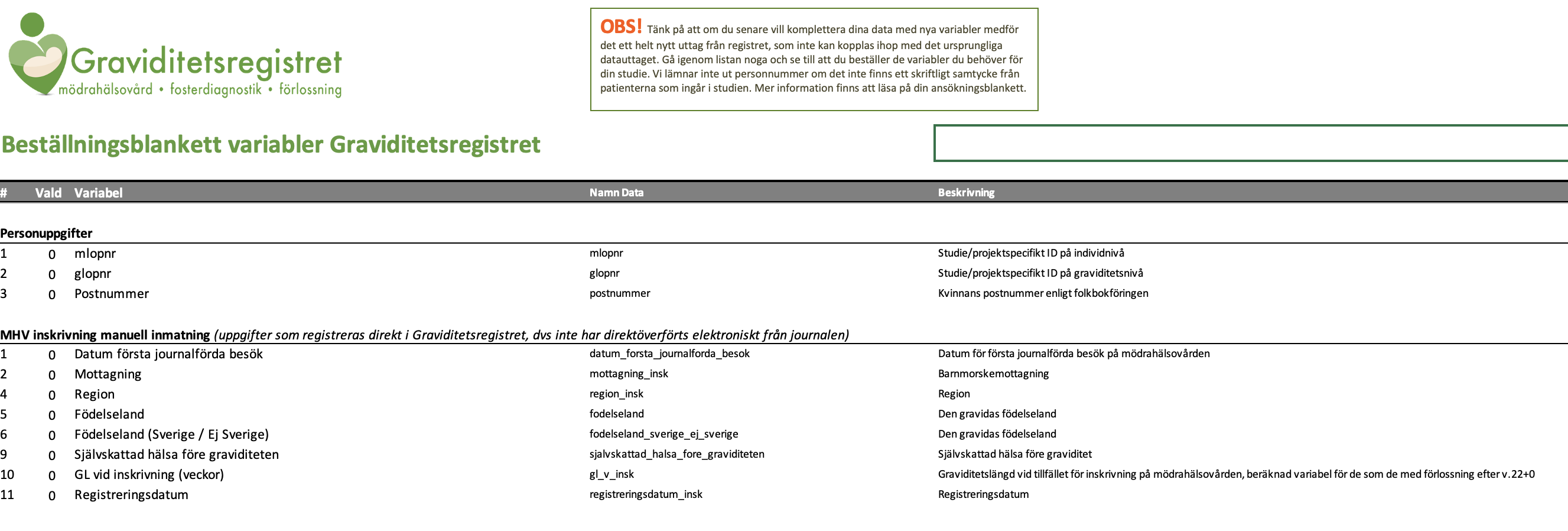


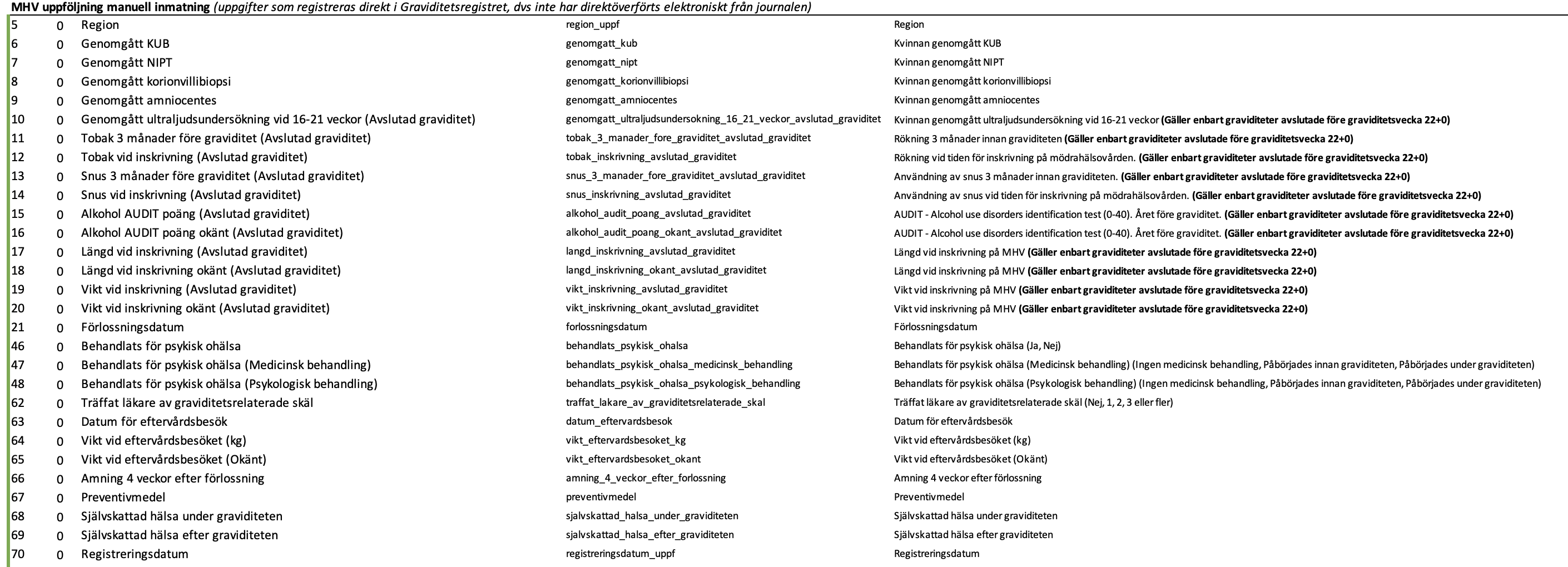


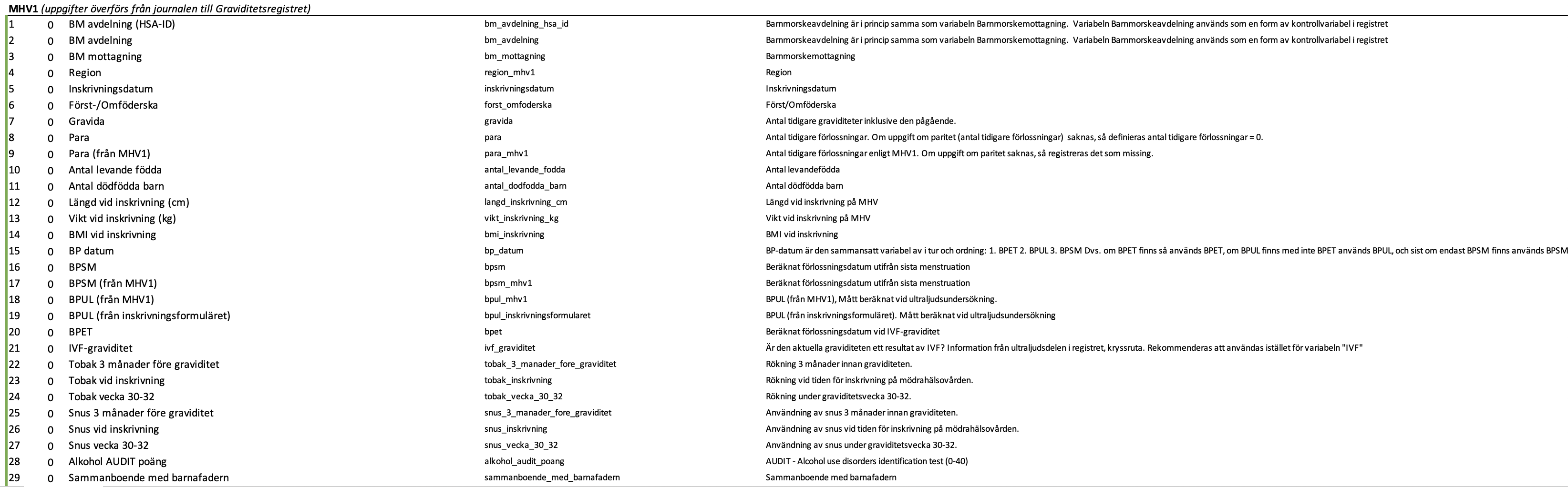


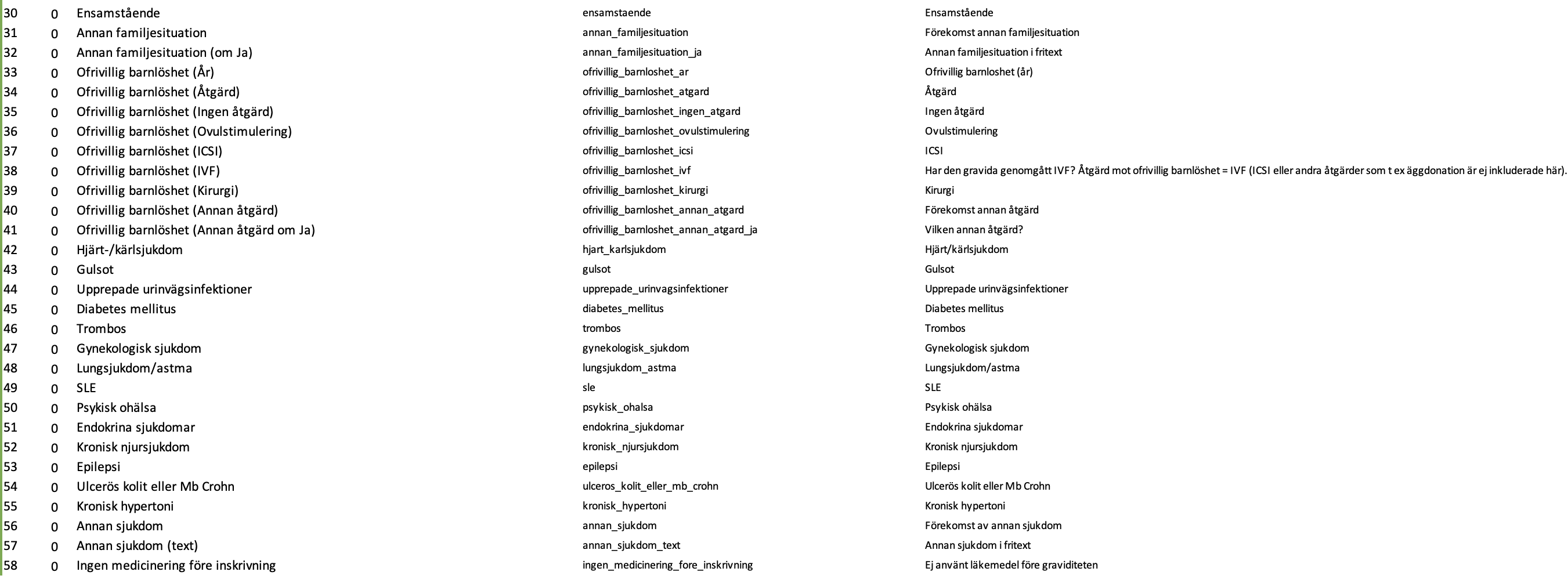


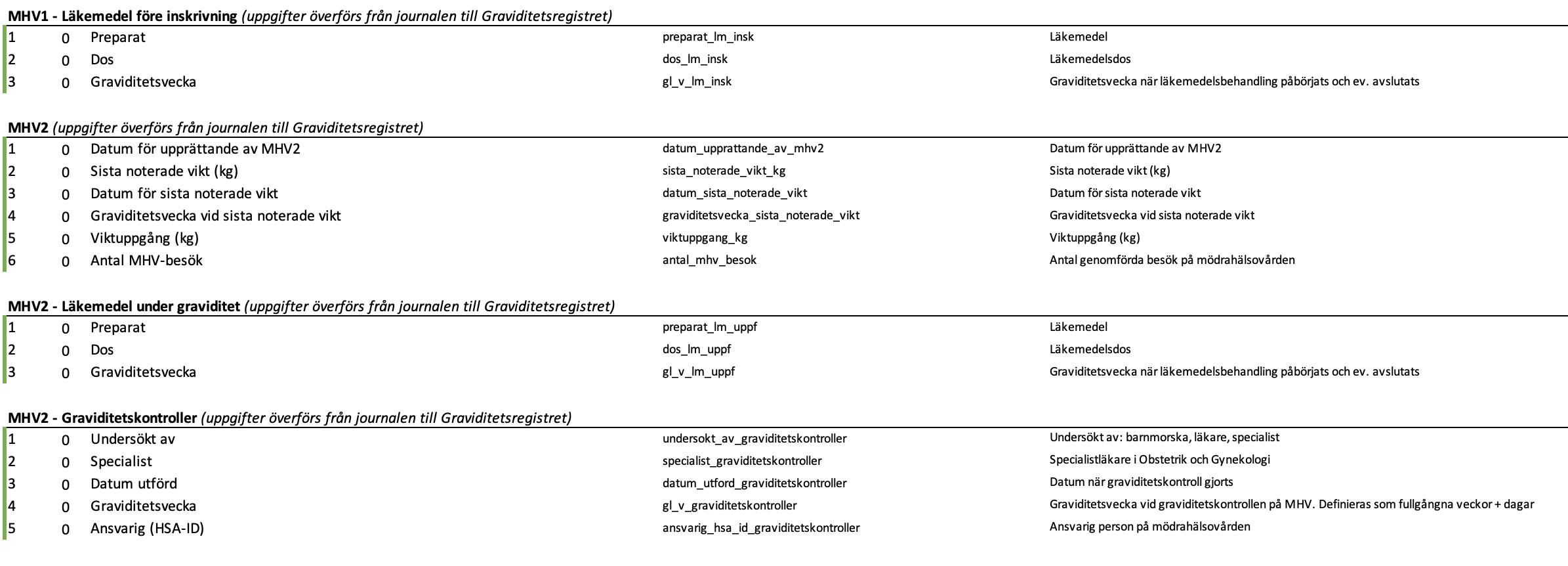


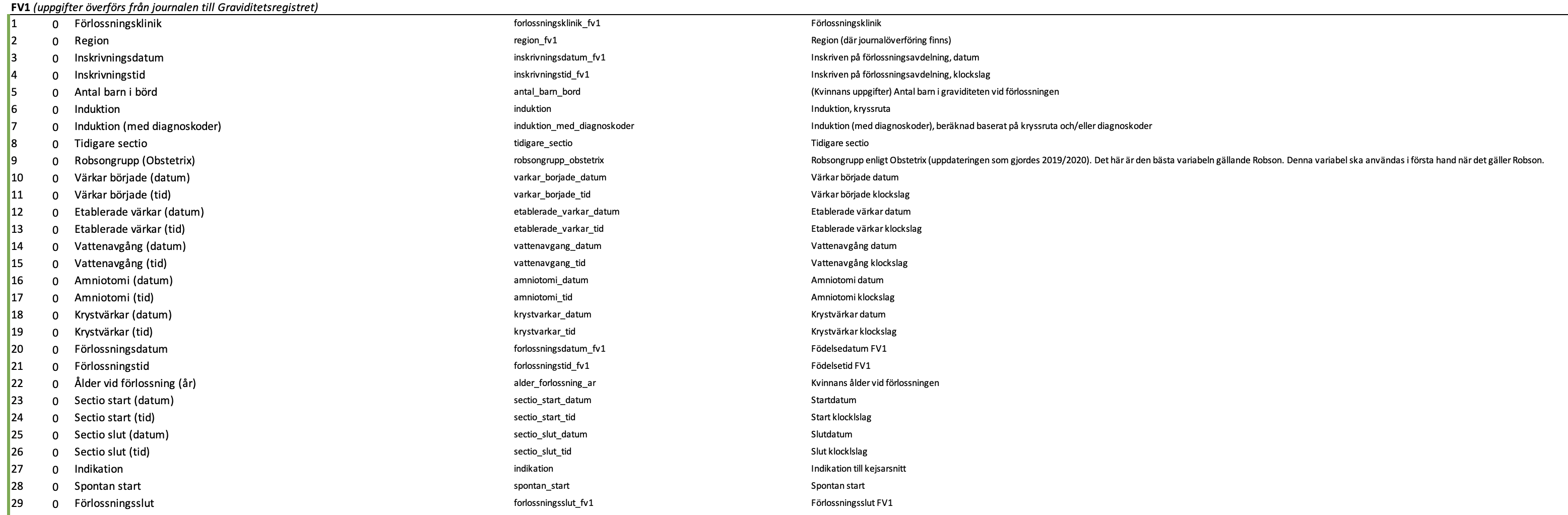


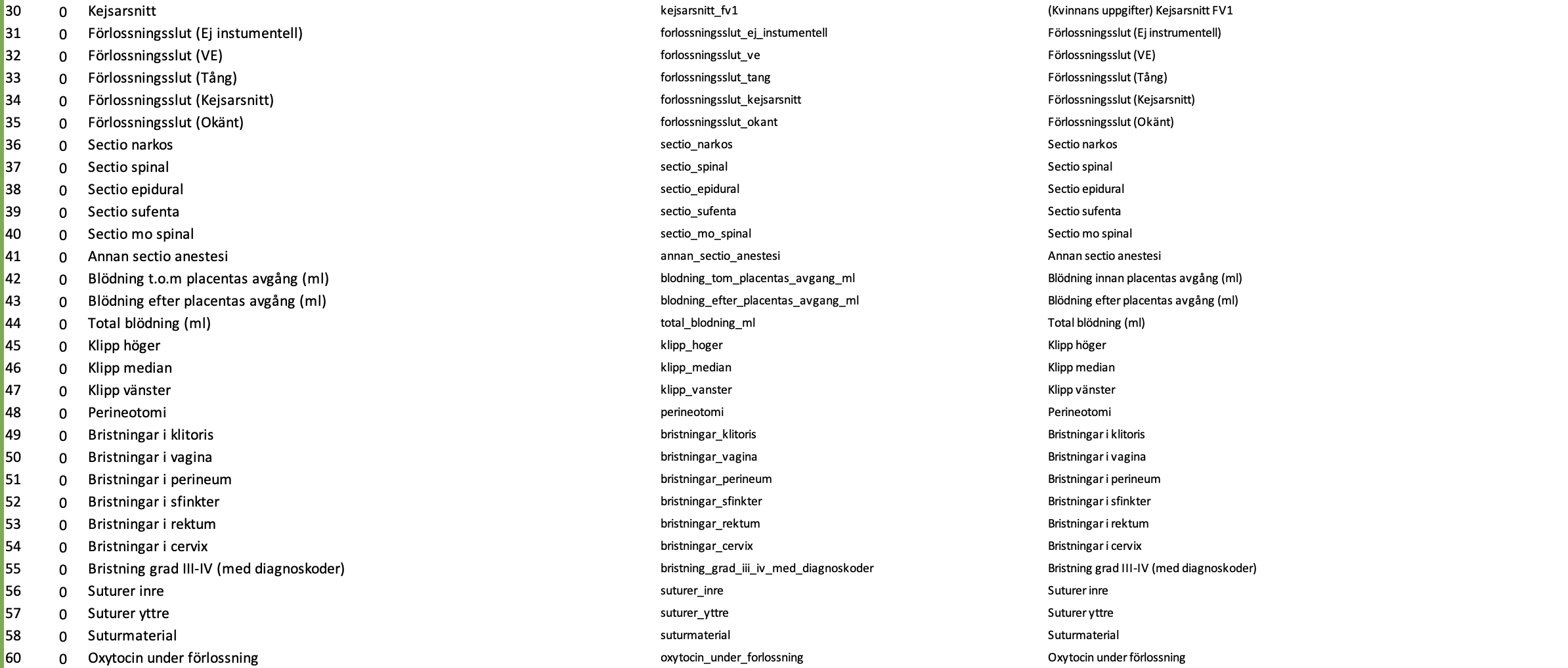

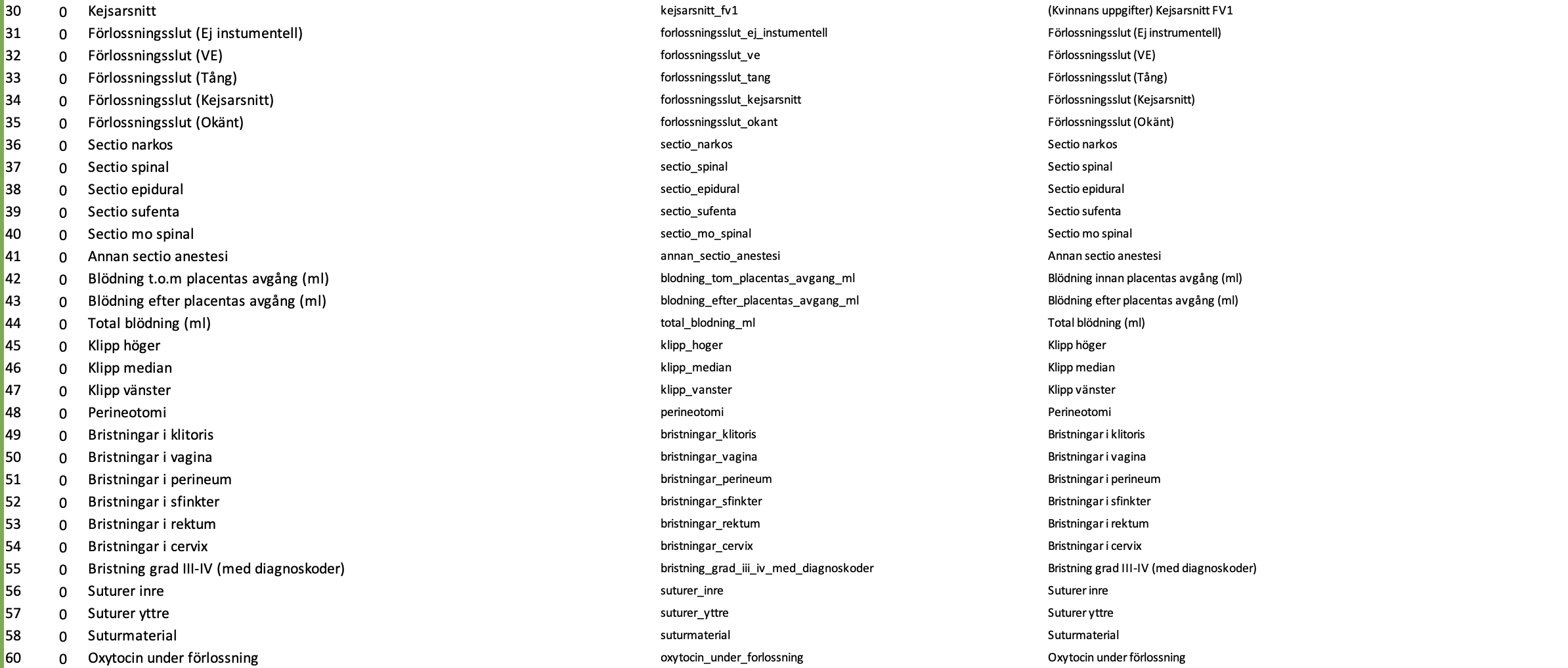


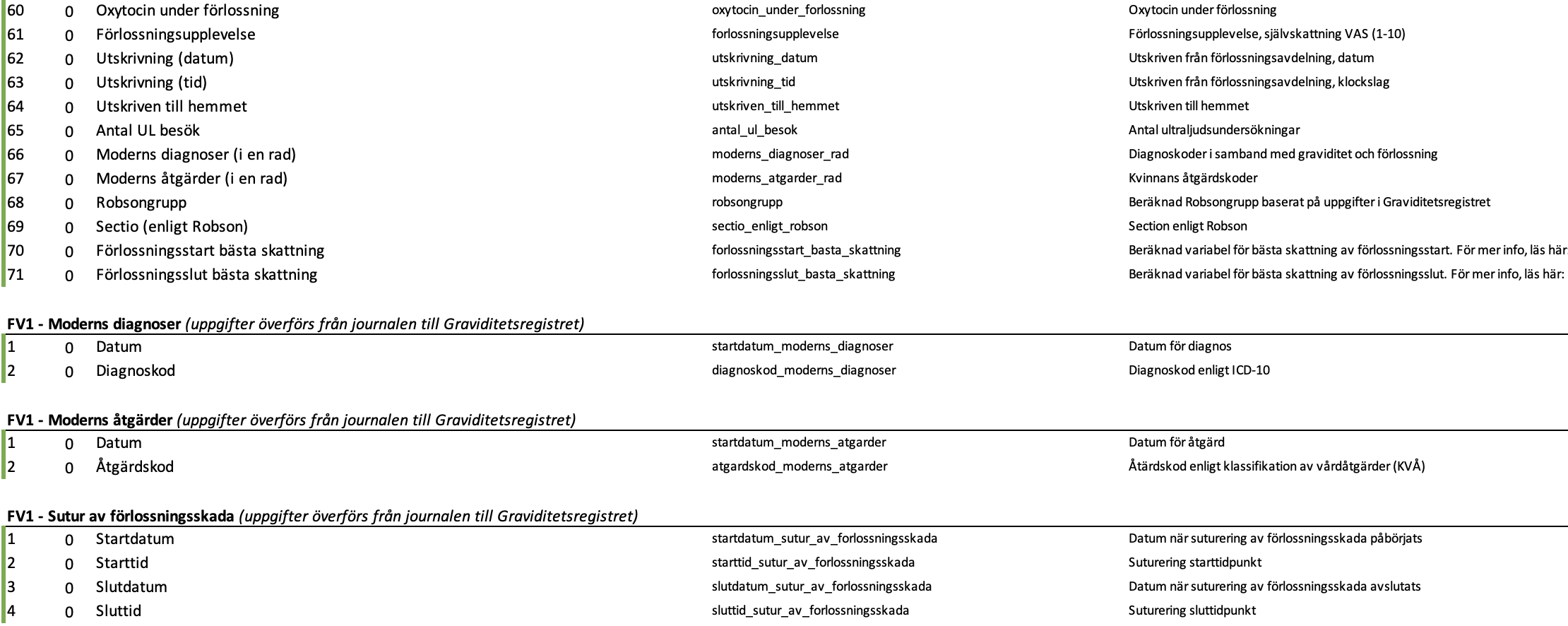


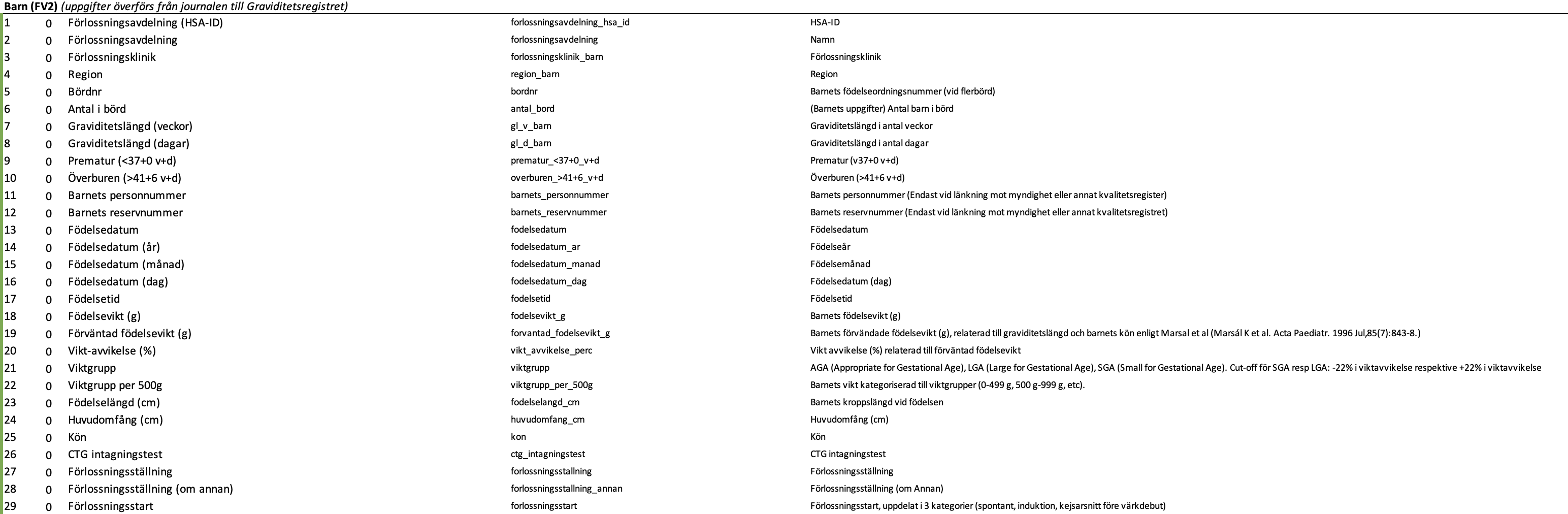


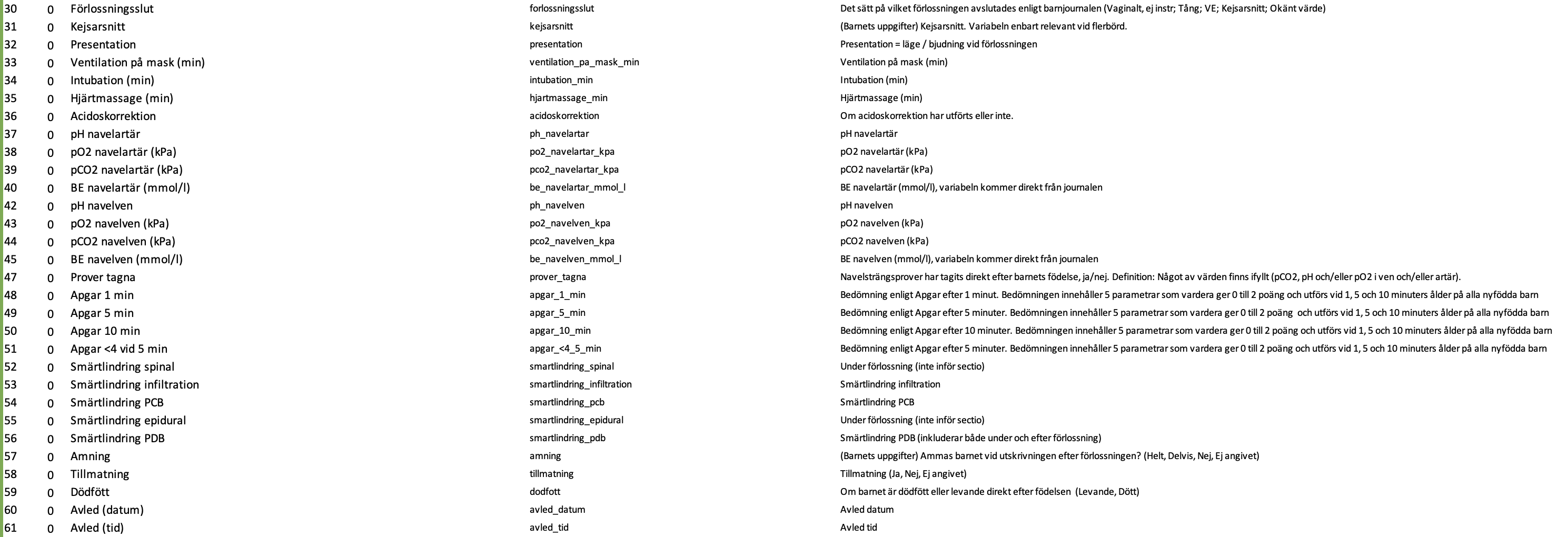


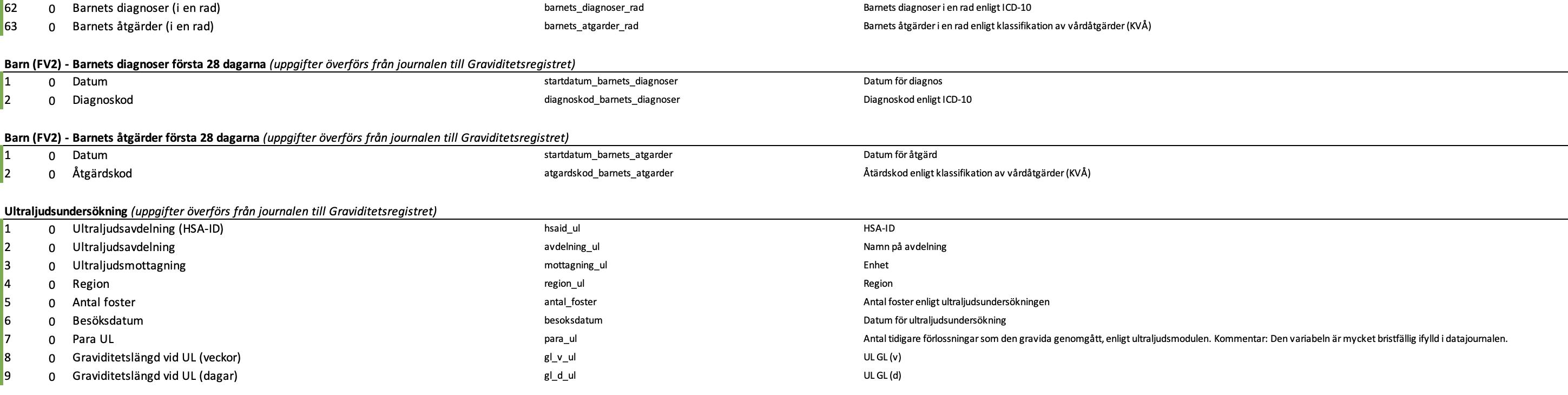


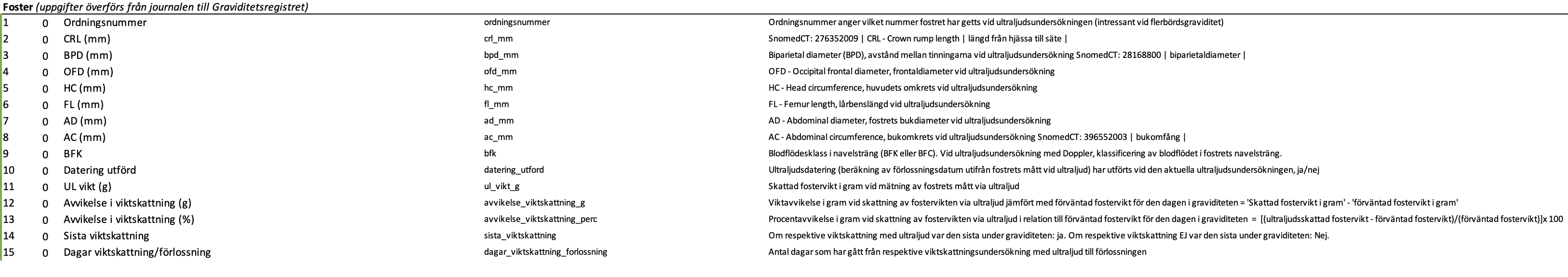


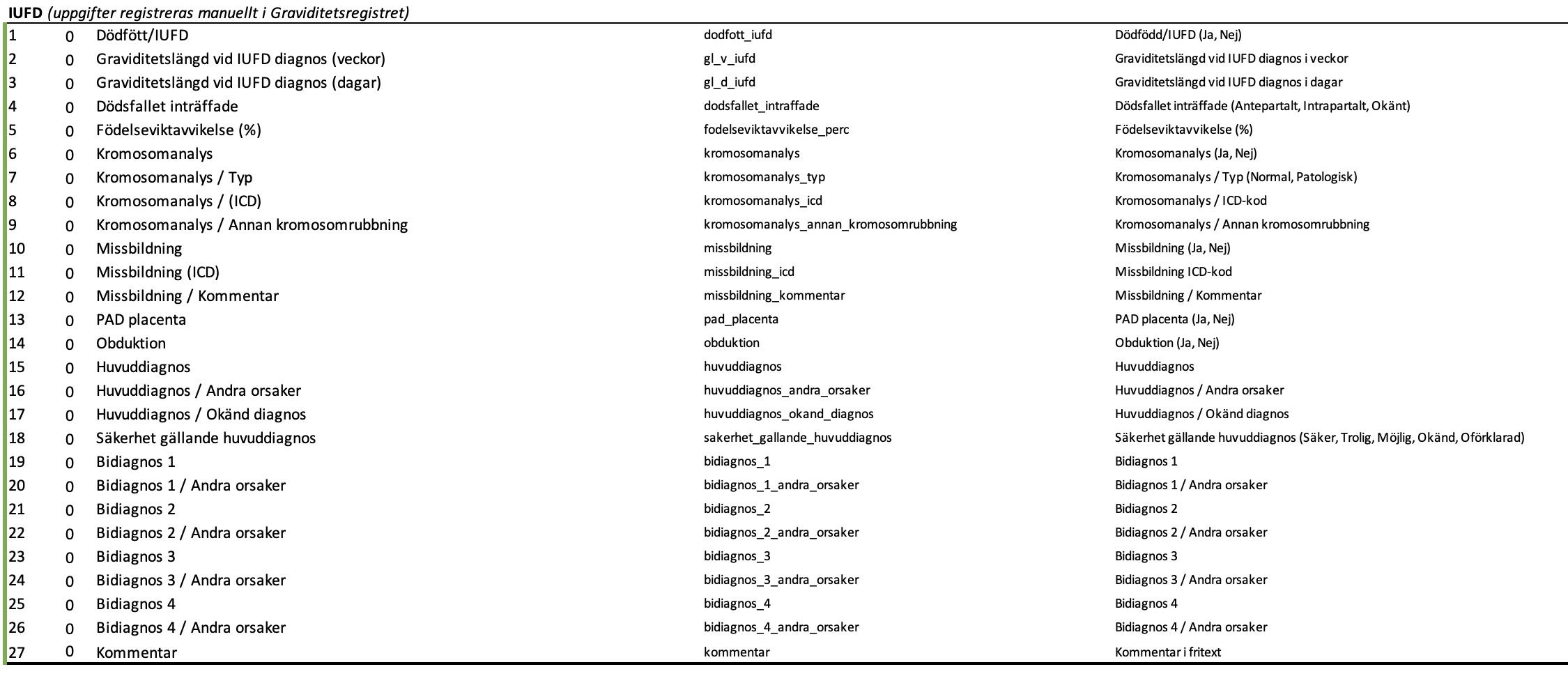


Variables from the Swedish National Patientregister

| Variabelnamn | Definition |
| --- | --- |
| ALDER | Ålder vid utskrivningsdatum |
| ALDER_S | Ålder vid årets slut. |
| ANE1 | Anestesikod 1 |
| ANE2 | Anestesikod 2 |
| AR | Utskrivningsår |
| ATC | ATC-kod vid förgiftning. Läkemedel enligt FASS. Max 30 koder |
| ATCO | ATC-kod för läkemedelstillförsel. Läkemedel enligt FASS. Max 30 koder |
| AVTAL | Vårdersättningen regleras enligt avtal |
| CIVIL | Patientens civilstånd |
| DIA_ANT | Antal inrapporterade diagnoser, kan vara mer än 30 |
| DIAGNOS | Diagnoser enligt ICD. Max 30 diagnoser |
| DISTRIKT | Patientens hemdistrikt, uppgift från SCB |
| DRG | Diagnosrelaterad gruppering, kan anta ca 550 värden. |
| EKOD1 | Ytttre orsakskod 1. Se ICD-kodlista |
| EKOD2 | Yttre orsakskod 2. Se ICD-kodlista |
| EKOD3 | Yttre orsakskod 3. Se ICD-kodlista |
| EKOD4 | Yttre orsakskod 4. Se ICD-kodlista |
| EKOD5 | Yttre orsakskod 5. Se ICD-kodlista |
| EKOD | Yttre orsakskod 1-5 |
| FLAND | Patientens födelseland |
| FODAR | Patientens födelseår |
| FODDAT | Patientens födelsedatum |
| FODDATN | Patientens födelsedatum |
| HDIA | Angiven huvuddiagnos |
| IDNR | Ett unikt löpnummer för varje vtf. Används bl.a vid DRG-gruppering. Skapas av Socialstyrelsen. |
| INDATUM | Det datum då patienten skrivs in. Antal dagar från år 1960-01-01 fram t.o.m. INDATUM |
| INDATUMA | Det datum då patienten skrivs in |
| INSATT | På vilket sätt patienten skrivits in |
| KON | Patientens kön |
| KONTROLL_DATUM | Syftar till det datum då automatisk leveranskontroll (LOK) gjorts, vilket sker i anslutning till att uppgiftslämnare skickar in data. För varje vårdkontakt är det alltså möjligt att se när denna senast skickades in till Socialstyrelsen. Variabeln är främst användbar vid interna kvalitetskontroller. |
| LK | Patientens folkbokföringsort uppgift från SCB |
| LKF | Patientens folkbokföringsort efter kontroll mot SCB |
| LKF_IN | Patientens folkbokföringsort enligt uppgiftslämnare. |
| LT_IN | Vem som skickat in data - Privat vårdgivare eller region. |
| LT_KLIN | Klinik som patienten skrevs ut från |
| MDC | Vårdtillfällen grupperat på kapitel-nivå |
| MVO | Medicinskt verksamhetsområde som patienten skrevs ut från. |
| NATION | Patientens medborgarskapsland |
| OP | Åtgärdskoder max 30 åtgärder |
| OP_ANT | Antal inrapporterade åtgärder, kan vara mer än 30 |
| OPD1 | Datum då åtgärd 1 utfördes |
| OPD10 | Datum då åtgärd 10 utfördes |
| OPD11 | Datum då åtgärd11 utfördes |
| OPD12 | Datum då åtgärd 12 utfördes |
| OPD13 | Datum då åtgärd 13 utfördes |
| OPD14 | Datum då åtgärd 14 utfördes |
| OPD15 | Datum då åtgärd 15 utfördes |
| OPD16 | Datum då åtgärd 16 utfördes |
| OPD17 | Datum då åtgärd 17 utfördes |
| OPD18 | Datum då åtgärd 18 utfördes |
| OPD19 | Datum då åtgärd 19 utfördes |
| OPD2 | Datum då åtgärd 2 utfördes |
| OPD20 | Datum då åtgärd 20 utfördes |
| OPD21 | Datum då åtgärd 21 utfördes |
| OPD22 | Datum då åtgärd 22 utfördes |
| OPD23 | Datum då åtgärd 23 utfördes |
| OPD24 | Datum då åtgärd 24 utfördes |
| OPD25 | Datum då åtgärd 25 utfördes |
| OPD26 | Datum då åtgärd 26 utfördes |
| OPD27 | Datum då åtgärd 27 utfördes |
| OPD28 | Datum då åtgärd 28 utfördes |
| OPD29 | Datum då åtgärd 29 utfördes |
| OPD3 | Datum då åtgärd 3 utfördes |
| OPD30 | Datum då åtgärd 30 utfördes |
| OPD4 | Datum då åtgärd 4 utfördes |
| OPD5 | Datum då åtgärd 5 utfördes |
| OPD6 | Datum då åtgärd 6 utfördes |
| OPD7 | Datum då åtgärd 7 utfördes |
| OPD8 | Datum då åtgärd 8 utfördes |
| OPD9 | Datum då åtgärd 9 utfördes |
| PSEUDO | Pseudonymiserat personummer. Skapas av Socialstyrelsen. |
| PNR | Patientens personnummer |
| PNRQ | Variabel som visar kvaliteten på ett personnummer (PNR) enligt vissa förutbestämda regler. Variabeln är skapad med hjälp av standardmacrot checkpnr. |
| PSVARD | Psykiatrisk vårdform |
| PVARD | Anger om vårdtillfället varit planerat eller ej |
| RNR | Anges om patienten ej har något personnummer |
| RTC | En fellista som listar registrets felaktigheter efter procentuell fördelning |
| SENINV | Senaste invandring |
| SENUTV | Senaste utvandring |
| SJUKHUS | Sjukhus vid vilken patienten skrivits ut från. |
| SKAPAD | Datum då registret lagts upp |
| SLUT | Slutdatum för psykiatrisk vårdform |
| Slutrapporterad | Markerar om en vårdkontakt rapporterats in för sista gången i enlighet med föreskriften. |
| START | Startdatum för psykiatrisk vårdform |
| UTDATUM | Det datum då patienten skrivs ut |
| UTDATUMA | Det datum då patienten skrivs ut |
| UTSATT | Utskrivningssätt |
| VTF_ID | Löpnummer. Vårdkontaktens unika identifikation som skapas av uppgiftslämnaren. |
| VTID | Utdatum - indatum, antal dagar |

**DATA SAFETY AND MONITORING CHARTER**

| 1. INTRODUCTION | |
| --- | --- |
| Name of trial | Endo-SOFT trial  **First line surgery versus first line fertility treatment using assisted reproductive technologies in patients with advanced endometriosis: A national multicenter randomized-controlled trial.** |
| Objectives of trial, including | The Endo-SOFT trial is a national multicenter randomized study**.** |
| Interventions being investigated | Interventions being investigated comparing outcomes of ART and surgery prior to ART in patients with advanced endometriosis and infertility.  The study will include a quality assessment phase before randomization to ensure required competency level of participating centers and surgeons. During the trial the clinical data will be reviewed centrally to ensure uniform quality. The primary endpoint of the Endo-SOFT trial is cumulative live birth rate 3 years after first treatment (surgery or ART). Secondary endpoints include Cumulative Pregnancy Rate, time to pregnancy and live birth, spontaneous pregnancy rate, miscarriage rate and/or extrauterine pregnancies, reproductive outcomes per IVF cycle, rate of recurrent implantation failure (RIF), infections after oocyte pick-up, quality of life and pain, peri- and two months postoperative complications, health care costs and quality of life and obstetrical outcomes. |
| Outline of scope of charter | The purpose of this document is to describe the roles and responsibilities of the independent Data Safety and Monitoring Board (DSMB) for the Endo-SOFT trial, including the timing of meetings, methods of providing information to and from the DSMB, frequency and format of meetings, statistical issues  and relationships with other committees |
| 2. ROLES AND RESPONSIBILITIES | |
| A broad statement of the aims  of the committee | To safeguard the interests of trial participants, assess participants safety, and monitor accrual and complications during the trial |
| Terms of reference | The DSMB will receive and review the safety data of this trial. The DSMB should inform the Trial Steering Committee (TSC) if, in their view:  o Recruitment the time of Interim Analysis is insufficient to achieve the planned sample size within the 3-year inclusion period.  o If complication rates are markedly higher than expected in either arm, or if they vary substantially between centers.  The DSMB will be supplied with all relevant data at the above mentioned time points to evaluate the inclusion rate, complications and any unexpected differences between study arms as well as potential conflicts with new insights and/or developments within the field of advance endometriosis and fertility. |
| Specific roles of DSMB | It is at the discretion of the DSMB to meet early in the course of the trial to discuss the protocol including the interim analysis plan, and to have the opportunity to clarify any aspects with the Coordinating investigator/Sponsor |
| 3. COMPOSITION | |
| Membership and size of the  DSMB | DSMB members register their assent by confirming (1) that they agree to be on the DSMB and (2) that they agree with the contents of this Charter. The members are independent of the Trial and have no competing interests that could impact on the Trial.  The members of the DSMB for this trial are:  (1)  *Associate Professor, Sophia Brismar Wendel*  (2) *Biostatistician, George Dimakopopulos*  (3)  *Associate Professor (to be decided)*  The Trial Coordinating Investigator may be asked and will be available to attend open sessions of the DSMB meeting. The other TSC members will not usually be expected to attend but can attend when necessary |
| 4. RELATIONSHIPS | |
| Clarification of DSMB role  Competing interests | The independent biostatistician will receive financial compensation for the statistical analyses performed as part of the DSMC responsibilities. The other DSMC members participate on a voluntary basis and will not receive financial compensation.  Competing interests of DSMB members – financial matters, involvement in other trials or intellectual investment should be disclosed. DSMB members should not use interim results to inform trading in pharmaceutical shares, and careful consideration should be given to trading in stock of companies with competing products. |
| 5. ORGANIZATION OF DSMB MEETINGS | |
| Expected frequency of DSMB  meetings | The DSMB will meet at least once in the first year after the start of participant inclusion. The DSMB will perform interim analyses as mentioned above (2. Roles and responsibilities, Terms of reference).  The meetings of the DSMB can be by conference call, as long as full discussion with all members can be guaranteed. All sessions are in principle open, although the DSMB can decide otherwise |
| 6. TRIAL DOCUMENTATION AND PROCEDURES TO ENSURE CONFIDENTIALITY AND PROPER COMMUNICATION | |
| Intended content of material  to be available in open sessions  Who will see the accumulating data and interim analysis  External evidence  To whom the DSMB will communicate the decisions/recommendations that are reached | Accumulated information relating to the trial’s safety data will be presented. Other outcome measures (e.g. accrual rate) may be presented, at the discretion of the DSMB.  The DSMB will discuss the results of the interim analysis with the TSC. DSMB members do not have the right to share confidential information with anyone outside the DSMB, other than the TSC.  The Coordinating Investigator/Sponsor will identify and circulate external evidence that can influence the trial.  The DSMB reports its recommendations in writing to  the TSC.  The DSMB members should store the documents safely after each meeting so they may check the next report against them. After the trial is reported, the DSMB members should destroy all interim reports. |
| 7. DECISION MAKING | |
| Decisions/recommendations  open to the DSMB  Decisions or recommendations  within the DSMB | Possible recommendations:  • No action needed; trial continues as planned  • Temporarily stop accrual, awaiting further inquiry and observation  • Early stopping due, for example, to clear harm of any of the allocated treatment arms  • Recommend protocol modifications if complication rates are higher than expected.  Every effort should be made for the DSMB to reach a  unanimous decision. If the DSMB cannot achieve this, a vote may be taken, although details of the vote should not be routinely included in the report to the TSC as these may inappropriately convey information about the state of the trial data.  It is important that the implications (e.g.  ethical, statistical, practical, and financial) for the trial be considered before any recommendation is made.  Effort should be made for all members to attend DSMB meetings. Chair will try to ensure that a date is chosen to enable this.  If a member does not attend a meeting, it should be  ensured that the member is available for the next  meeting. If a member does not attend a second  meeting, they should be asked if they wish to remain  part of the DSMB. If a member does not attend a third meeting, they should be replaced and the Trial  Coordinating Investigator be notified. |
| 8. REPORTING | |
| Recommendations/decisions of  the DSMB  Disagreement between the  DSMB and TSC | The DSMB will report their recommendations/decisions in a letter to the TSC, within 4 weeks after the meeting  If the DSMB has serious problems or concerns with the TSC decision based on the report, a meeting of these groups should be held. The information to be shown would depend upon the action proposed and the DSMB’s concerns.  Depending on the reason for the disagreement confidential data will have to be revealed to all those  attending such a meeting. The meeting will be chaired by an external expert who is not directly involved with the trial. |
| 9. AFTER THE TRIAL | |
| Publication of results | If requested by the DSMB, a meeting at the end of the trial will be held to allow the DSMB to discuss the final data with the principal trial investigators and give advice about data interpretation.  The DSMB will be given the opportunity to read and  comment on any publication before submission, especially with respect to reporting of any DSMB recommendation regarding termination of a trial.  The DSMB may discuss issues from their involvement in the trial when permission is agreed with the overseeing committee. |
