# Supplementary material for: Endo-SOFT: study protocol for a national multicentre randomised controlled trial – first-line surgery versus first-line assisted reproductive technologies in patients with advanced endometriosis
Source: BMJ Open. 2026 Mar 25;16(3):e111928. doi: 10.1136/bmjopen-2025-111928 (PMC13034312; doi:10.1136/bmjopen-2025-111928)
Supplement: online supplemental file 2 [file bmjopen-16-3-s002.docx]

Table S1. Schedules of events table

| Procedures | **Baseline** | **Enrolment** | **1 month prior to surgery or start of first ART cykle^1^** | **Surgery^2^** | **2 months postoperative visit^2^** | **1:st oocyte retrieval** | **2 months after 1:st oocyte retrieval** | **2:nd oocyte retrieval** | **2 months after 2:nd oocyte retrieval** | **3:rd oocyte retrieval** | **2 months after 3:rd oocyte retrieval** | **Visit, phone call or questionnaire 3 years after first treatment (surgery or first ART cykle)** |
| --- | --- | --- | --- | --- | --- | --- | --- | --- | --- | --- | --- | --- |
| **Informed consent oral and written** | x |  |  |  |  |  |  |  |  |  |  |  |
| **Demographics** |  |  | x |  |  |  |  |  |  |  |  |  |
| **Record ultrasound and/or MRI staging according to AAGL and #ENZIAN staging system** | x |  |  |  |  |  |  |  |  |  |  |  |
| **Randomization** |  | x |  |  |  |  |  |  |  |  |  |  |
| **Record surgical procedure according to AAGL and #ENZIAN staging system** |  |  |  | x |  |  |  |  |  |  |  |  |
| **Record intraoperative complications^3^** |  |  |  | x |  |  |  |  |  |  |  |  |
| **Hospital stay** |  |  |  | x |  |  |  |  |  |  |  |  |
| **Record 8 weeks postoperative complications^4^** |  |  |  |  | x |  |  |  |  |  |  |  |
| **EHP-30, EQ5D and NRS scale^5^** |  |  | x |  | x | x | x | x | x | x | x | x |
| **Primary outcome (CLBR)** |  |  |  |  |  |  |  |  |  |  |  | x |
| **Secondary outcome/IVF cycle^6^** |  |  |  |  |  |  | x | x | x | x | x | x |

^1^ start of FSH injections ^2^only first line surgery group ^3^According to Classic ^4^According to Clavien Dindo ^5^Endometriosis Health Profile and Numeric Rating Scale ^6^LBR, PR, number of oocytes retrieved, fertilization grade, number of blastocysts
