# Supplementary material for: Endo-SOFT: study protocol for a national multicentre randomised controlled trial – first-line surgery versus first-line assisted reproductive technologies in patients with advanced endometriosis
Source: BMJ Open. 2026 Mar 25;16(3):e111928. doi: 10.1136/bmjopen-2025-111928 (PMC13034312; doi:10.1136/bmjopen-2025-111928)
Supplement: Supplementary file 3 [file bmjopen-2025-111928.reviewer_comments.pdf]

PEER REVIEW HISTORY

BMJ Open publishes all reviews undertaken for accepted manuscripts. Reviewers are asked to complete a checklist review form and are provided with free text boxes to elaborate on their assessment. These free text comments are reproduced below.

ARTICLE DETAILS

Title (Provisional)

Endo-SOFT: Study Protocol for a National Multicenter Randomized Controlled Trial: First-Line Surgery vs. First-Line Assisted Reproductive Technologies in Patients with Advanced Endometriosis

Authors

Marklund, Anna; Wängberg Nordborg, Julia; Asciutto, Christine; Elenis, Evangelia; Francis, Jynfiaf; Jokubkiene, Ligita; Zuk, Kristin W; Åmark, Hanna; Rodriguez-Wallberg, Kenny A; Sundfeldt, Karin; Forslund, Maria; Brunes, Malin

---

VERSION 1 - REVIEW

**Reviewer**	**1**
**Name**	**Seyer-Hansen, Mikkel**
**Affiliation**	**Aarhus University, Department of Clinical Medicine**
**Date**	**24-Dec-2025**
**COI**	**None**

This is a well planned and well described randomised multicenter study on a very important subject regarding advanced endometriosis and fertillity treatment. The potential positive influence of operation prior to IVF-treatment has been discussed for several years and a good study is much in demand. I have only two considerations:

1. How about the spontaneous pregnancies. Women with very low AMH-level have poor chance of IVF pregnancy. Are these women excluded from the study. Otherwise a low pregnancy rate in these women could interfere with the outcome.

Do all women in the study accept to avoid obtaining spontaneous pregnancy after operation? Will they be excluded in case of a spontaneous pregnancy?

2. Is there any specific quality indicators for the level of operative experience at the participating departments? Allthough all operations are performed in one of the four centers of specialized treatment of endometriosis there might be different levels of operative volume and thereby routine. Could this interfere with the results of the study.

VERSION 1 - AUTHOR RESPONSE

Reviewer: 1
Dr. Mikkel Seyer-Hansen, Aarhus University
Comments to the Author:
This is a well planned and well described randomised multicenter study on a very important subject regarding advanced endometriosis and fertillity treatment. The potential positive influence of operation prior to IVF-treatment has been discussed for several years and a good study is much in demand. I have only two considerations:

1. How about the spontaneous pregnancies. Women with very low AMH-level have poor chance of IVF pregnancy. Are these women excluded from the study. Otherwise a low pregnancy rate in these women could interfere with the outcome.

We thank the reviewer for raising this important point. Women with low AMH levels are not excluded from the study, as the trial aims to reflect a real-world population of women with advanced endometriosis undergoing fertility treatment.

In addition, ovarian reserve is recorded for all participants and we have prespecified subgroup analyses by AMH (<0.5; 0.5–3.5; >3.5 ng/mL) to explore whether treatment effects differ in women with low ovarian reserve. These subgroup analyses are exploratory and will be interpreted cautiously.

This has been described in statistics in line 337-340

"Subgroups of special interest will include type of ART (IVF or ICSI), age (<35 vs ≥35 years), ovarian reserve (AMH <0.5; 0.5–3.5; >3.5 ng/mL), adnexal surgery (yes/no), and surgery for deep endometriosis in the anterior (yes/no) and posterior (yes/no) compartment, defined using Swedish procedure classification codes."

Do all women in the study accept to avoid obtaining spontaneous pregnancy after operation? Will they be excluded in case of a spontaneous pregnancy?

Spontaneous pregnancies are handled as follows: live births resulting from spontaneous conception after initiation of the allocated fertility treatment strategy are included in the primary outcome (cumulative live birth rate). Participants are not required to avoid spontaneous conception after surgery or treatment initiation.

However, participants who achieve a spontaneous pregnancy before initiation of the allocated treatment are not included in the primary analysis and will be replaced, in order to preserve the integrity of the randomised comparison. We have clarified this definition and handling of spontaneous pregnancies in the revised manuscript. Line 273-280

”The primary outcome is the cumulative live birth rate (CLBR) after up to three complete ART cycles, defined as the proportion of participants achieving at least one delivery of a live-born infant beyond 22 weeks of gestation within three years following the initiation of the allocated fertility treatment strategy. Live births resulting from spontaneous conception occurring after treatment initiation are included in the CLBR.
Participants who achieve a spontaneous pregnancy before initiation of the allocated treatment are not included in the primary analysis and will be replaced to preserve the integrity of the randomised comparison.”

2. Is there any specific quality indicators for the level of operative experience at the participating departments? Allthough all operations are performed in one of the four centers of specialized treatment of endometriosis there might be different levels of operative volume and thereby routine. Could this interfere with the results of the study.

We thank the reviewer for this important comment. All participating centres are nationally designated highly specialised endometriosis centres, selected by the Swedish National Board of Health and Welfare through a formal application and quality assessment process, including institutional experience, annual surgical volume, infrastructure, and standardised staging of endometriosis.

In addition, all participating surgeons are individually approved prior to trial initiation and are required to have documented experience of at least 20 advanced endometriosis surgeries. Surgeon experience and annual caseload are reviewed, and only approved surgeons may act as lead surgeons.

To further minimise potential centre-related effects, randomisation is stratified by centre. Surgical quality and complication rates are continuously monitored, and centres or individual surgeons may be suspended from recruitment if quality concerns arise, following consultation with the Data Safety Monitoring Board. We have clarified these aspects in the revised manuscript.

A full list of study procedures and interventions are available in the full protocol (online supplemental material 1).

The manusrcipt has been revised as follows Line 254-269:

” **Quality assurance of surgery and fertility treatment**

All participating centres are nationally designated highly specialised endometriosis centres, approved by the Swedish National Board of Health and Welfare following a formal application and quality assessment process. This includes documentation of institutional experience, annual surgical volume, infrastructure, and the ability to perform standardised endometriosis staging.

All participating surgeons are approved by the coordinating investigators and steering committee and must have prior experience of at least 20 advanced endometriosis surgeries. Surgeon experience and annual caseload are documented prior to trial initiation, and only approved surgeons are permitted to act as lead surgeons. For each surgical procedure, data on surgeon volume and centre volume are prospectively collected.

Randomisation is stratified by centre to account for potential centre-related differences in operative volume and routine. Surgical and fertility outcomes are continuously monitored, and centres or individual surgeons may be temporarily or permanently suspended from further recruitment in case of quality concerns, following consultation with the Data Safety Monitoring Board."

VERSION 2 - REVIEW

**Reviewer**	**1**
**Name**	**Seyer-Hansen, Mikkel**
**Affiliation**	**Aarhus University, Department of Clinical Medicine**
**Date**	**05-Feb-2026**
**COI**	

I think the authors have responded very well to the limited comments I had for the first version of the protocol. I believe the manuscript should be ready for acceptance.
